# An encounter with death: a comparative thematic and content analysis of naturalistic DMT experiences and the near-death experience

**DOI:** 10.3389/fpsyg.2025.1532937

**Published:** 2025-03-05

**Authors:** Pascal Michael, David Luke, Oliver Robinson

**Affiliations:** ^1^School of Human Sciences, Old Royal Naval College, Centre for Mental Health, University of Greenwich, Greenwich, United Kingdom; ^2^Department of Brain Sciences, Faculty of Medicine, Imperial College, Centre for Psychedelic Research, London, United Kingdom

**Keywords:** DMT, near-death experience, naturalistic, thematic analysis, psychedelic, Dimethyltryptamine, NDE

## Abstract

**Introduction:**

Classical near-death experiences (NDEs) refer to states of disconnected consciousness characterised by a range of features occurring in the context of being close to death. Various psychedelic substances, such as *N,N-*dimethyltryptamine (DMT), consistently replicate NDE features and may be considered ‘near-death-*like* experiences.’ However, a systematic qualitative analysis comparing the specifics of content with the broader themes of both psychedelic and NDEs has yet to be conducted.

**Methods:**

We report the third thematic and content analysis of the DMT experience from a naturalistic field study, focusing on themes related to death and dying. Based on 36 semi-structured interviews, this analysis is then directly compared, qualitatively and in terms of content frequency, with a novel extension of a previous thematic analysis of 34 written NDE narratives.

**Results:**

The ‘canonical NDE themes’ identified across the DMT experiences included Translocation, Bright Light(s), Sense of Dying, The Void, Disembodiment, Tunnel-like Structures, Light Being-esque Entities, Deceased Family, Life Review-like, and Hyper-empathic Experiences. A total of 95% of participants reported at least one of these. Twelve ‘less typical NDE motifs’ were also noted. Five classical NDE features were entirely absent from DMT, while DMT exhibited an even broader array of experience features that were absent from NDEs. DMT clearly shares a more basic phenomenological structure with NDEs but shows differences in the prevalence of certain features. Furthermore, DMT did not present any immediately recognisable linear sequencing of themes. Overall, DMT is distinctly unique in its qualitative content, characterised by its more prodigious and stereotypical nature, which includes kaleidoscopic, extraterrestrial, transcultural, fluctuating, and overwhelming elements.

**Discussion:**

When examining the comparability between DMT and NDEs at a fundamentally more nuanced level of qualitative content (as opposed to broad themes or questionnaire items), the two experiences clearly diverge. However, a minority of NDEs, which are themselves unique, do share significant content with DMT. Taken together, DMT could be considered an ‘NDE-mimetic.’ The weaker comparability is likely due not only to differences in context but also to the complex neural processes occurring near death, in which endogenous DMT may only play a small role. In light of this level of parallelism with NDEs, some potential clinical applications of DMT are also discussed.

## Introduction

*“…like being fired out the muzzle of an atomic canon with neon byzantine barrelling.”—Alan Watts, on describing his first DMT experience, mirroring the NDE ‘tunnel.’ Quoted in Leary (1966).*

### What is DMT, and what is the NDE?

*N,N-*dimethyltryptamine (hereafter DMT) is a short-acting classical psychedelic that primarily exerts its effects via the 5-HT2A receptor. It is also an agonist at the sigma-1 (S1) receptor and is endogenous to humans. Several studies have provided detailed reviews of its pharmacology and physiology (Carbonaro and Gatch, 2016; Rodrigues et al., 2019; Barker, 2018; Barker, 2022). At ‘breakthrough’ doses, DMT consistently leads to a restructuring of one’s internal world model (Gallimore, 2019, 2013, 2020). The near-death experience (NDE) is a syndrome of experiential features that typically occur under conditions of proximity to death, after the (reversible) loss of vital signs, or during life-threatening situations. Comparable phenomenology, in contrast to content (reliable features vs. their variable expressions, delineated further below under *Rationale for study*), can be induced through various methods such as meditation, hypnosis, or drug use—referred to as near-death-*like* experiences (Charland-Verville et al., 2014), which may also include only the anticipation of death.

### DMT and NDE phenomenology

Michael et al. (2021, 2023a) detailed the phenomenological structure and specific content of the groundbreaking DMT experience itself, including a thorough comparison with existing literature on DMT phenomenology. The “near-death experience” was popularised, and its experiential landscape was charted by Moody (1975), who identified 15 ‘core features.’ Questionnaires were designed to quantitatively measure and standardise the phenomenon, including Ring’s (1980) idea of a continuum of five progressive stages and Greyson’s (1983) more widely used 16-item Near-Death Experience Scale (NDES). More recently, Charland-Verville et al. (2014) have reliably illuminated the most common features of the NDE across the NDE scale, which include peace or joy, bodily separation, seeing a bright light, encountering deceased individuals or other beings, and time distortion. Martial et al. (2017) found no reliable temporal sequence to NDEs; however, some approximate order of appearance for the NDES items (similar to the aforementioned common features) suggested an initiation with peace, followed by light, then spirits or people, and finally, border and return. An improved measure, the NDE-Content (NDE-C) scale, now incorporates several features not listed in the NDES: unusual sensory experiences, void or non-existence, entering a gateway, a sense of ineffability, decisions or coercion to return, and feelings of being dead.

### DMT phenomenology relevant to NDEs

**Figure 1:**
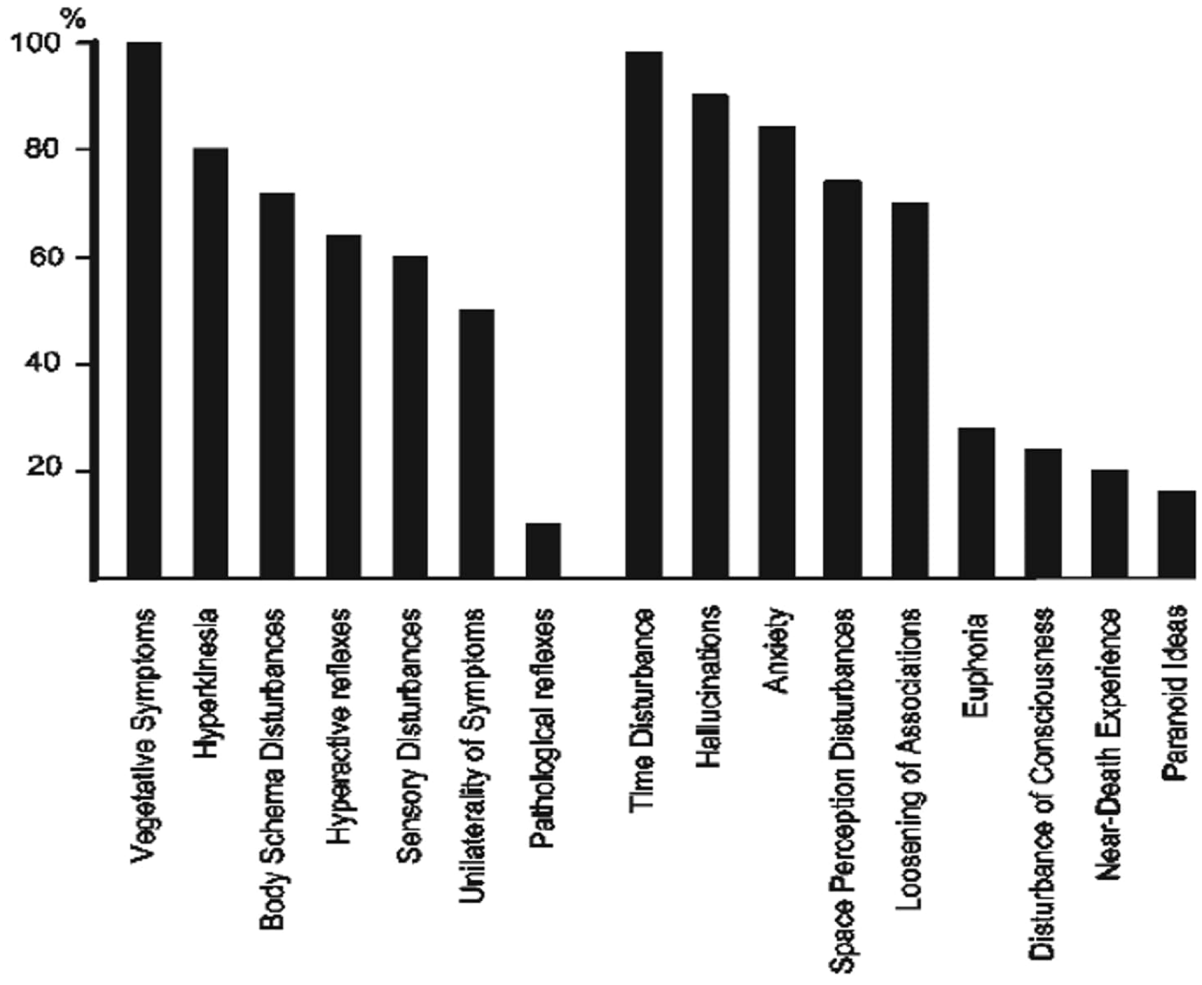
Illustration of the first human DMT study by Sai-Halasz et al. (1958), showing ‘near-death experiences’ in 6 out of 30 participants.

Sai-Halasz et al. (1958), in their pioneering human intramuscular DMT study in 1958, note at the outset that “several subjects initially report experiences akin to the end of the world, accompanied by a profound fear of death.” Notably, the phrase “near-death experience” is included in their classification of participant experience types (see Figure 1) nearly 20 years before Moody’s popularisation of the term. Remarkably, Sai-Halasz et al.’s (1958) NDE category shows the lowest occurrence (6/30), yet still represents a notable 20%. Particularly relevant in this initial DMT study is the following passage from one participant, Dr. E. C. H., which contains evident NDE-related themes such as noise at onset, feelings of dying, travel to another place, benign, god-like beings of light, emerging from darkness into light, reluctance to return, and, more broadly, mystically relevant motifs of boundlessness and noetic insight. This evidence challenges the idea that the DMT-NDE association is merely a meme-like construct resulting from its later popularisation.

“I hear whistling. I am *en route* somewhere… This is death… In front of me are two quiet, sunlit Gods… they are welcoming me into this new world… these are the sons of the Sun, and I am finally at home…in that real and beautiful world… Their sunburned faces are radiant, and their movements are free and graceful… I see through the black iron lattice into the bright temple… This is the true colour and shape of things. Dangerous game; it would be so easy not to return… I have become a different person…more free. I now understand much more” (Sai-Halasz et al., 1958, p. 7).

Meyer and Lyttle’s (1992) mapping of the DMT space culminates in the ‘white light.’ Based on ayahuasca phenomenology, Shanon (2002) concludes his 14 stages with “the primordial point of light…from which all has been created…all of creation, of life, of intelligence…this point of light has been called God.” Shanon’s inventory of ayahuasca entities includes humanoid, winged beings composed of light, religious deities, demons, and beings of death. In Cott and Rock’s (2008) retrospective thematic analysis of DMT from 19 respondents, no excerpts are provided in which experiences or fears of death or dying occurred. Despite this, they note that the entities themselves often fulfilled the ‘positive performative function’ of imparting information about themselves and the universe, reminiscent of the roles enacted by NDE beings, typically deceased persons (Kelly, 2001; Lange et al., 2004). Their theme of “Spirituality” is stated to be consistent with NDE phenomenology, given its religious dimensions and transformative nature (Lange et al., 2004; Greyson, 2006)—though the similarities mainly lie in both being highly mystical (e.g., ego and time–space transcendence, unitive experience), rather than sharing the features or even content of classical NDEs (Athappilly et al., 2006).

Furthermore, the study conducted by Davis et al. (2020) regarding entities encountered during DMT experiences indicated that at least 7% of these encounters involved communications from beings asserting that the ‘DMT state is indicative of the post-death state’ and that ‘death represents only the beginning.’ A total of 69% of the participants reported having received a message or assignment, wherein ‘valediction’ was characterised as “being told farewell or indicating unpreparedness for the experience,’ which parallels the experiences of individuals who have undergone near-death experiences (NDEs) and have often been sent back, typically by deceased loved ones. Furthermore, 19% of respondents noted experiencing a ‘prediction’ concerning the future; in NDEs, the ‘life preview,’ a form of personal premonitory or globally prophetic vision, is occasionally presented (Ring, 1992). It is noteworthy that the *least* frequently reported types of DMT entities included deceased family members (2%), deceased acquaintances (2%), and deceased friends (1%). Additionally, religious figures/angels and elves/faeries were also amongst the least common entities reported (<16%). This observation is particularly intriguing, considering these entities are directly associated with spiritual and/or folkloric typologies, with both categories being comparable, as elves/faeries are traditionally believed to *represent* the spirits of the deceased (Evans-Wentz, 1911). In contrast, angelic or religious entities, as well as the deceased, are the quintessential entities encountered in NDEs (Charland-Verville et al., 2014; Cassol et al., 2018). The *most* prevalent yet generic descriptors utilised by Davis et al. for guides/helpers or spirits (34–43%) remain consistent with those encountered in NDEs, with the exception of the specific term ‘alien’ (*39%*), which underscores DMT’s modeling of abduction phenomena, potentially to a greater extent than what is typically observed in NDEs (e.g., Michael et al., 2021).

### Simulation of the NDE by DMT or other psychedelics

A series of studies have rigorously examined the potential of DMT to emulate the phenomenon in question. The most notably significant study indicates that various narratives encompassing alternate realms and entities have led to speculation that DMT, produced by the pineal gland, indeed contributes physiologically to the near-death experience (NDE) (Strassman, 2001). This assertion is tempered by the observation that only one of over 60 participants reported experiences markedly reminiscent of an NDE and expressed an explicit intention to evoke such a phenomenon (Strassman, *personal communication,* April 22, 2022). Potts and Does (2012) conducted a comparative analysis of the NDE scale (NDES) against a curated compilation of themes derived from Strassman’s studies, arriving at the conclusion that the comparability is insufficient to substantiate the DMT hypothesis related to NDEs. Similarly, Liester (2013) juxtaposed the principal characteristics delineated by Moody with accounts from prominent NDE experiencers against Shanon’s (2002) documentation of ayahuasca. They noted parallels and potential shared mechanisms; however, they also acknowledged superficial distinctions, such as the occurrence of autoscopic out-of-body experiences (OBEs) in NDEs contrasted with geometric patterns associated with DMT.

More rigorously quantitative approaches include a comparison of scores on all NDE scale items between classical NDEs and the so-called “NDE-like” experiences, which encompass temporal lobe epilepsy, sleep states, and drug intoxication. They found insignificant differences, concluding that NDEs and experiences in non-life-threatening conditions are phenomenologically indistinguishable (Charland-Verville et al., 2014). Similarly, Timmermann et al. (2018) prospectively compared NDE scale results between participants in a laboratory DMT study and classical NDE experiencers, concluding that all DMT subjects qualify as having an NDE and are equally likely to report each scale item. In a similar vein, Corazza (2008) retrospectively compared the NDE scale between ketamine and near-death experiences, primarily identifying equivalence, except for bright light, and, consistent with our findings below, encounters with deceased individuals/deities and a boundary of no return being more common in NDEs. Given this, single NDEs were compared to NDE-resembling ketamine trips amongst users’ potential thousands, likely inflating the observed similarities (Luke, 2009). All the aforementioned studies are based on gross structure (phenomenology), confirming that DMT reliably reproduces the NDE, at least on this level, yet they do not address its ability to subtly simulate the nuanced content of the NDE (specific quality). Martial et al. (2019) identified primarily ketamine, followed by *Salvia divinorum,* alongside other serotonergic substances—including DMT—and monoaminergic or dissociative psychedelics in online trip reports that most significantly mirror the NDE in terms of their semantic properties. However, this was qualitatively limited by relying on indices such as the type and frequency of word usage through natural language processing.

Crucially, this endeavour to confirm or disconfirm the overlap of the experiential repertoire between DMT and NDEs has a broader precedent in the question of whether drugs possess religious significance (Smith, 1964; Braden, 1969), given the overtly spiritual manifestations of NDEs. This has been more rigorously explored through Griffiths et al.’s (2019) survey of encounters with ‘God’ or ‘Ultimate reality’ compared between naturally occurring and serotonergic psychedelic experiences. The authors concluded that the two forms are phenomenologically nearly indistinguishable and mainly differ in terminology, which supports the legitimacy of drug-induced mystical experiences. However, contemporary efforts to compare natural versus pharmacological mystical experiences and their implications for ‘genuineness’ are in stark contrast to the extraordinarily rich cultural history of the use of such ‘entheogens,’ which have influenced entire religious cosmologies amongst shamanic societies.

Martial et al. (2024) recently conducted a quantitative comparison of various classical psychedelics and narratives of classical near-death experiences (NDEs) from a survey of individuals reporting both. They found that reports of time perception, peace, love, ineffability, ‘floating’, immersion in a novel reality, and ‘mystical effects’ were similar between the two experiences. Nevertheless, a preliminary qualitative analysis of the same data (Michael et al., in preparation) suggests that OBE-like floating and total immersion in a novel reality are unique to NDEs or certain psychedelics at high doses. The mystical similarities have been indicated previously by the well-documented, reliable induction of mystical experiences by psychedelics (Kangaslampi, 2023), along with very few items on the NDE scale distinguishing NDEs from mystical experiences (Greyson, 2014). The identified differences included that psychedelics tend to involve more visual hallucinations and, regarding enduring effects, foster a greater connection to people, nature, and the cosmos. In contrast, NDEs involve ‘leaving the body’ more often and tend to alleviate the fear of death more significantly. This finding contrasts with a recent study on NDEs (alongside other ‘non-ordinary states’) that suggests comparable reductions in death anxiety for both NDEs and psychedelics (Carbonaro and Gatch, 2016), though it aligns with the observation of NDEs having a more profound impact on enduring effects in a within-subjects comparison (Michael et al., in preparation). The intense, visual, elemental hallucinatory qualities of psychedelics (specifically ayahuasca) and the OBE-like disembodiment associated with NDEs were highlighted by Liester (2013). Therefore, the most promising findings may lie in the differences observed in their enduring effects.

### Physiological contribution to the NDE by DMT

There is some indirect, supportive evidence that DMT may physiologically contribute to near-death states. Firstly, one enzyme necessary for its synthesis from tryptamine, INMT, has been identified in the human brain (Saavedra et al., 1973; Saavedra and Axelrod, 1972; Dean et al., 2019), and DMT is present in human urine, blood, and cerebrospinal fluid (CSF) (Barker et al., 2012). Dean et al. (2019) demonstrate a high co-localisation of INMT and AADC, the initial requisite enzyme converting tryptophan to tryptamine, in the rodent cortex, which was significantly lower in peripheral tissues. Crucially, Dean et al. also provide evidence that DMT itself is present in cortical tissue, though again only in rodents, and at levels comparable to canonical neurotransmitters. Importantly, DMT was shown to increase an average of 6-fold during the induction of cardiac arrest in rodents, and while it has also been identified in the rat pineal gland (Barker et al., 2013), this increase occurred even after pineal resection, albeit to a lesser extent (although this may have been influenced by baseline levels).

The idea that DMT may be released during severe hypoxic states, such as near-death experiences, to mitigate neuronal death is supported by the growing evidence of psychedelics as ‘psychoplastogens,’ which can promote neuritogenic, synaptogenic, and even neurogenic changes in the nervous system. This suggests a potential treatment for neurodegenerative disorders or brain injuries (Saeger and Olson, 2022; Kozlowska et al., 2022; Vann Jones and O’Kelly, 2020; Khan et al., 2021). DMT itself has been shown to have neurogenic effects *in vivo* (Morales-Garcia et al., 2020), and ayahuasca has been found to increase brain-derived neurotrophic factor (BDNF) in humans (de Almeida et al., 2019). Furthermore, potent neuroprotective and anti-inflammatory properties have also been documented (Szabo et al., 2014, 2016; Szabo and Frecska, 2016).

### Rationale for the current study

The primary rationale for this study can be understood through a triangulation of approaches or levels of analysis regarding the psychopharmacology of NDEs—neurobiological, quantitative, and qualitative. While the most direct approach would be to seek endogenous DMT changes in individuals who are near death, this presents practical and ethical challenges. However, from a neurobiological perspective, brain DMT levels have been shown to increase during near-death events in rodent models (Dean et al., 2019). The application of questionnaires and other quantitative methods has illuminated the fundamental phenomenological structure and semantic properties of NDEs, as they might be simulated by DMT (Timmermann et al., 2018) and other substances (Charland-Verville et al., 2014; Martial et al., 2019), providing insights into the frequency of various features. Nevertheless, the more nuanced nature of how these structures manifest in both experiences—the qualitative content, sensitive to individual and group subjective differences, as opposed to the structure, which may have more objectively predicated origins (Varela and Shear, 1999; Petitmengin, 2006)—is clarified only through detailed qualitative content analyses. This dichotomy can be likened to *texture* (content) versus *template* (structure), and it is within the content dimension that disparities between the two experiences can be addressed. Once crystallised, the picture will be clearer regarding the implications of the level and type of experiential overlap—which could extend beyond DMT’s potential role in NDEs to include clinical applications and even personal (legal) insights for understanding death, including grief. Thus, this paper aims to conduct such an analysis of the DMT experience, focusing on themes related to near-death experiences, death, and rebirth, which will then be systematically compared to narratives of classic NDEs derived from the raw data upon which a recent thematic analysis of NDEs was based (Cassol et al., 2018).

## Methods

For a complete description of the *participants and recruitment, measures and materials, procedure and anonymity,* and *analyses,* please refer to the *Methods* section of the original thematic analysis of a naturalistic field study of DMT use conducted by the present authors (Michael et al., 2021), which this third report is based on. A sufficient version of the methods is provided below, including those specific to the current report.

### Participants

**Figure 2:**
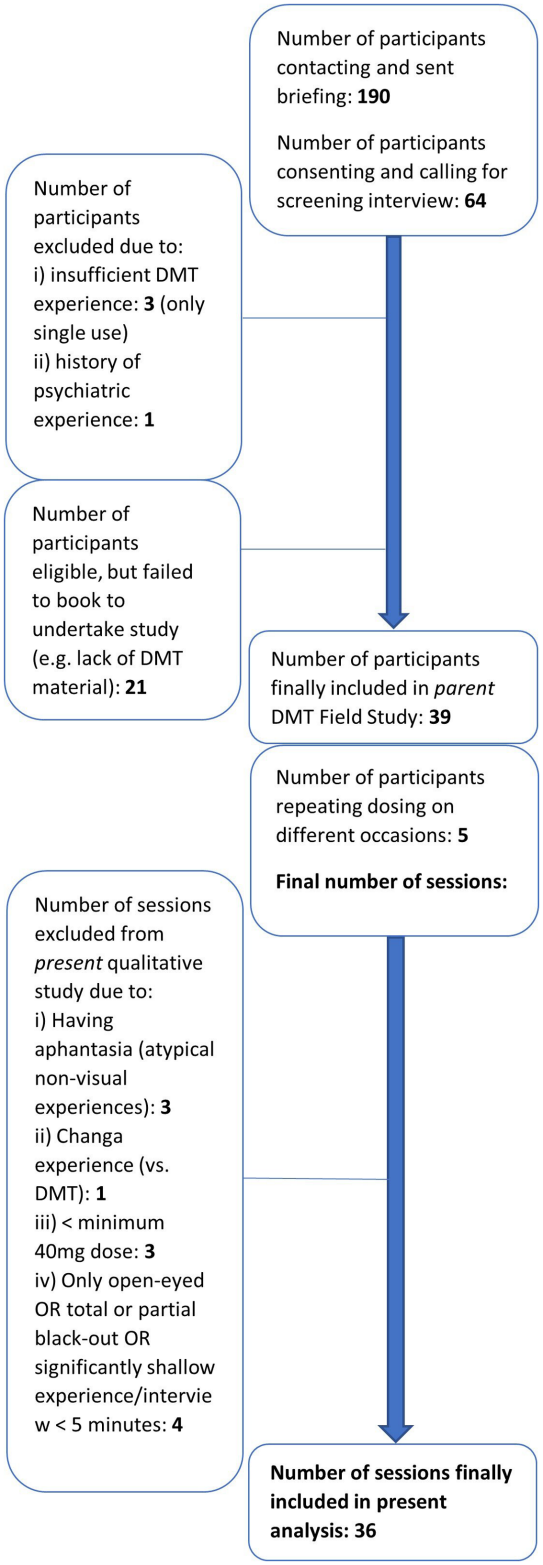
Flowchart of DMT participant recruitment—inclusion, exclusion, and final sample size for overall and current analysis.

Volunteers were selected through either convenience or snowball sampling. Inclusion criteria required participants to have experienced at least one significant *N,N-*DMT experience, along with other substantial *N,N-*DMT or analogue experiences (see Table 1 for further demographic data), and to provide their own supply of DMT. The exclusion criteria included previous psychedelic experiences associated with lasting difficulties or findings from the administered SCID-CT (First et al., 2007), which indicated current or recent psychiatric health conditions or challenges (Johnson et al., 2008). A total of 47 DMT sessions were conducted in the parent field study, with some participants attending multiple sessions; 36 sessions served as the basis for the present analysis (see Figure 2 for further details). The first participant was recruited on July 26, 2018, and the last on December 6, 2019.

**Table 1:** DMT participant demographics and DMT experience.

Participant number	Pseudonym	Age (range)	Sex	Nationality	First time DMT used	Last time DMT used	Overall times DMT used	% breakthrough DMT experiences
1	MP	45–49	M	White British	2011	11/2016	20	33
2	TM (3 Doses)	30–34	M	White Romanian	2015	11/2016	5–6	100
3	BB	35–39	M	White British	2013	02/2018	15–20	25
5	JM	35–39	M	White British (Scottish)	2015	03/2017	12	66
6	RV	40–44	M	White British	2015	08/2018	1 (+4 AYA)	75
7	TC	25–29	M	White German	2014	06/2018	10–15	100
8	HV	35–39	F	Black British (Ghanaian-Egyptian descent)	2016	02/2018	80	<100
10	GR	25–29	M	White Romanian	2015	2015	2 (+ 4-ACO-DMT)	Once
11	SP (2 Doses)	35–39	M	White British	2003	06/2017	10–15	>50
12	RH (3 Doses)	55–59	M	Asian British (Indian descent)	2013	08/2018	Hundreds	75
14	AZ	25–29	M	Isreali	2013	02/2018	7	>40
15	ZD	30–34	F	White British	2017	03/2018	20	90
16	RS	25–29	M	Black British (African descent)	2016	05/2018	40	50
17	LR	25–29	M	Chinese-Italian (Dual)	2010	2011	25	40
23	AF	40–44	F	White Italian	2018	05/2019	2 (+ 8 AYA, 10 changa)	>75
24	LG	30–34	M	Mixed British (Sri Lankan-German descent)	2011	07/2019	20	20
25	AN	25–29	F	White British	2018	07/2019	7	>40
26	EM	20–24	F	White Romanian	2017	05/2019	10	90
27	AB	35–39	M	White British	N/A	N/A	10	100
30	SH	30–34	F	White British	2007	2008	6	50
32	OR (2 Doses)	25–29	M	Brazilian	2012	2018	3 (+ Hundreds AYA)	Once
34	FF	45–49	M	White British	N/A	N/A	10	80
35	JB	40–44	M	White British	N/A	N/A	8	75
36	BW	45–49	M	White British	2000	07/2019	3 (+ 4 changa)	Once
40	JA	35–39	M	White British	2014	10/2019	70	>70
41	AV	45–49	F	Brazilian	2003	06/2019	20	100
42	MS	55–59	F	Mixed British (Iraqi-Italian descent)	2013	2017	5	100
43	DD	40–44	M	White British	N/A	N/A	Over a hundred	>40
44	DS	45–49	M	White British	N/A	N/A	Hundred	50
47	ST	35–39	M	Nigerian	N/A	N/A	3	66

Average age, 37.4 (Range: 23–58), 8/30 women, 83% Caucasian; AYA, Ayahuasca; N/A, Unprovided (due to being free to withhold information due to admission of illicit activity).

Regarding the current study’s comparison with the NDE, a summary of demographic information for the 34 patients who provided freely expressed NDE narratives (data shared by the Coma Science Group) includes the following: 11 women and 23 men; the mean age at NDE is 48.2 years; the range of ages at NDE is from 15 to 72; the average duration from NDE to narrative submission is 10.2 years; the range of years from NDE to narrative submission is from 1 to 51; the average score on the Greyson NDE Scale is 13.4 out of 32; the range of scores on the Greyson NDE Scale is from 7 to 22 out of 32; three of the 34 narratives included negatively valenced episodes. All etiologies of near-death events were coma-induced due to anoxic conditions.

All NDE narratives were originally shared in French, with the translation into English occurring in a four-step process: (i) Utilising the latest Microsoft Word translation tool, (ii) accuracy assessed by one author who is semi-proficient in French (*PM*), (iii) potential errors corrected with Google Translate, and (iv) final accuracy maximised through a representative sample translated by an independent fluent French speaker (*FD*), with accuracy calculated at 97.5% (the remaining 2.5% was adjusted for this sample).

### Procedure

A minimum vaporised dose of 40 mg (maximum 75 mg), with a mean of 54.5 mg (*SD* 9.8), was administered, ensuring a ‘breakthrough’ experience characterised by entry into an immersive space and a high subjective intensity rating (i.e., > 7), with a mean rating of 9.5 on a scale of 1 to 10. A semi-structured interview (SSI) was conducted face-to-face immediately following the experience by PM or DL, as detailed in the Supplementary material (*see* SM 8). This included questions about onset, sensory, bodily, emotional, and psychological experiences, encountered phenomena, and visionary landscapes. The interview typically lasted at least 30 min, with an approximate range of 12 to 75 min. The SSI utilised ‘bracketing’ inspired by the micro-phenomenological technique (Petitmengin, 2006), which minimised biases by discouraging the subject from relying on preconceived categorisations to explain their experience. Instead, participants articulated each component in their own words, with this instruction provided before the interview began. A full transcript of a sample interview for *TM 1* is included in the Supplementary material (SM 14).

### Analysis

Interviews were audio recorded, fully transcribed, and coded using *NVivo* v. 12, facilitating the calculation of frequency per content theme. The transcripts underwent thematic analysis according to the guidelines established by Braun and Clarke (2006), along with comprehensive content analysis. This process was entirely inductive, with *superordinate themes* and *subthemes* emerging solely from the interview data. The current analysis concludes the qualitative investigation of the DMT experience from the naturalistic field study, thematically focusing on experiences of death, near-death, and mystical experiences. However, the characteristics of the final superordinate *Canonical NDE themes* resonate with those on the NDE-Content Scale (NDE-C). Therefore, the analysis of these specific themes was not directly shaped by prior conceptions (deductive); rather, the inductive codes were naturally organised into subthemes that align with this NDE-C scale as a reliable phenomenological reference. This approach ensured a justified distinction between the canonical and the *less typical NDE motifs* that were also identified, allowing for a more systematic comparison with the raw data from the NDE narratives shared by Cassol et al. (2018), whose final themes closely reflect NDE content. Refer to the *Results* (*Canonical NDE themes*) for more details. The validity and reliability of the thematic analysis were strengthened by DL conducting several post-DMT interviews and possessing a deep understanding of the entire DMT qualitative data, while DL and OR reviewed the coding process multiple times and the various iterations of this manuscript, particularly concerning the subsequent comparison with the NDE narratives. This collaboration resulted in a high degree of agreement with PM’s final themes and comparisons.

The collection of content items related to subthemes in the current report, along with those from the previous two reports of the DMT field study (Michael et al., 2021), was systematically compared and contrasted in terms of presence, frequency, and qualitative content with the raw data provided by the authors of a previously published thematic analysis of the NDE (Cassol et al., 2018). This report represents a significant extension of the published analysis of the NDE, particularly through the inclusion of new items, which were added using a similar method of thematic analysis employed in the DMT analyses. This extension is justified due to the detailed nature of the DMT analyses, enabling the most direct and accurate comparison. Direct excerpts are included from both the raw transcripts of the DMT study and the NDE narratives to provide a level of qualitative detail that goes beyond merely relying on thematic items, facilitating a nuanced comparison focused on qualitative content instead of solely structural phenomenology [the former addresses variant components while the latter addresses invariant components (e.g., Varela and Shear, 1999)]. Importantly, this shared data from NDE narrative reports, intended for qualitative comparison with the DMT experience, originates from the same laboratory (though *not* the same participants; Coma Science Group) that provided the data on the NDE patients included in the *quantitative* comparative study of DMT and NDEs (Timmermann et al., 2018).

To serve as a quantitative measure, the NDES (Greyson, 1983) was administered to participants in the current DMT field study 24 h after the SSI. The results were then compared with the previously mentioned, published comparison of scores on the NDE scale from an earlier laboratory DMT study and classic NDEs (Timmermann et al., 2018). Refer to the Supplementary material (SM 2) for complete results and a discussion of this analysis. Comparing themes—especially their frequency—between the DMT and NDE states is particularly valuable, as it allows for a unique type of quantitative analysis that reveals features not captured by the NDES (Greyson, 1983). The comparison of themes and frequencies across these states also aids in determining whether there is congruence with the comparative prevalence indicated by the NDES scores of NDE and DMT experiences (to which only the DMT field study scores were explicitly compared). The differences in the specific nature of the content for each theme are often clear from their names, which will be elaborated upon in the *Thematic analysis of the DMT experience: “Near-death Experience, Death & Birth”* in the *Results section* below.

### Ethics

For the DMT field study, after reviewing the online briefing, participants had the option to indicate their consent, with verbal consent requested during the screening call and written consent obtained on the study day. For the shared NDE narratives, participants reached out to the original authors and expressed consent by signing a written consent form. The DMT field study and analysis received approval from the University of Greenwich Research Ethics Committee (Ref. 17.3.5.15). Naturalistic field research involving psychoactive substances has been successfully conducted in the past (Kuypers et al., 2016), as have psychometric and neural measures of DMT (Pallavicini et al., 2021). The collection of NDE data and its comparison with NDEs was approved by the University of Greenwich Research Ethics Committee (Ref. 18.5.5.17). The sharing of data from NDE participants for the purpose of this report was approved by the ethics committee of the Faculty of Medicine at the University of Liège.

## Results

### DMT and NDE content and prevalence comparison

#### Features illustrated in Table 2

**Table 2:** Analysis of themes and prevalence from both DMT thematic analysis (including the current analysis; Michael et al., 2021, 2023a) and NDE thematic analysis (from Cassol et al., 2018) with an analysis extension.

DMT thematic analysis (including present analysis; and Michael et al., 2021, 2023a)		NDE thematic analysis (from Cassol et al., 2018)—inc. *extension* of this analysis	
Equivalent themes/subthemes	Frequency / 36 (%)	Themes and subthemes	Frequency / 34 (%)
Onset	36 (100)	Entrance	6 (18)
	0	Soft	3 (9)
	0	Dark night	2 (6)
Bright light(s)	9 (25)	*Light*	25 (74)
	n/a	Attractive	10 (29)
	n/a	Enveloping	2 (6)
	n/a	Intense	16 (47)
	n/a	White	15 (44)
	n/a	Soft	3 (9)
	n/a	Not dazzling	3 (9)
	n/a	Yellow	1 (3)
	n/a	Diffuse/unknown source	8 (24)
	n/a	Alive	1 (3)
	0	Tunnel (Corridor = 1) – Leading to Light	9 (27)
	0	*Dark* tunnel leading to light	3 (9)
Tunnel-like structures (onset)	7 (19)	Tunnels at onset	9 (27)
Tunnel-like structures (during)	10 (28)		0
		*Time alteration*	16 (47)
Time–space transcendence	8 (22)	Total time loss	8 (24)
Time dilation (during)	5 (14)	Time dilation	6 (18)
Time dilation (onset)	6 (17)		
	0	‘Integrated’ time	1 (3)
*Encountering other beings*	34 (94)	*Entity encounter*	15 (44)
Presences (no imagery)	6 (17)	Sensed presence	2 (6)
Omnipresence	5 (14)		
Omnipresence	5 (14)	Universal Intelligence	2 (6)
Hyper-intelligent	7 (19)	“God”	1 (3)
	0	“Holy Spirit”	1 (3)
*‘Otherly’ creatures*	26 (72)	‘Imaginary’ entities	9 (27)
	0	Ferrymen	1 (3)
Silhouettes/featureless	8 (22)	Silhouettes / Faceless	3 (9)
Hooded figures	1 (3)		
Light being-esque	3 (8)	Beings of Light-like (e.g., Preternatural light, sentient, benevolent, hyperintelligent, powerful)	5 (15)
Fearsome	3 (8)	Headless/torturing	1 (3)
Succubus/unevolved souls	1 (3)	Lost/tortured souls	3 (9)
Hideous	1 (3)	Snake-like	1 (3)
Serpentine entities	3 (8)		
Trickster	3 (8)	Trickster	1 (3)
Mischievous or jestful	5 (14)		
Face(s) + hands only	2 (6)	Just face and hand	1 (3)
Doctor / scientist	1 (3)	Doctors	3 (9)
‘Psychic surgery’	1 (3)	‘Psychic surgery’	1 (3)
*Human*	6 (17)	*Humans/Human-like*	15 (44)
Relatives	2 (6)	Relatives	10 (29)
	0	Friends	1 (3)
	0	Unknown family	1 (3)
Unknown people	4 (11)	Unknown people	4 (12)
Deceased relatives	2 (6)	*Deceased*	9 (27)
		Relatives/friends	8 (24)
		Unknown dead	1 (3)
	0	Alive	3 (9)
Communication	14 (39)	Communication	7 (21)
	0	Returning to life	7 (21)
	0	Sense/Message of Mission	4 (12)
Intuition or telepathy	13 (36)	Telepathy	4 (12)
	0	Verbal	n/a
	n/a	Dialogue	3 (9)
	n/a	Unilateral	4 (12)
*Lucidity / ego preservation*	5 (14)	*Hyper-lucidity*	14 (42)
Ego death	11 (32)	Ego death	1 (3)
Clear mind	1 (3)	Clear and quick wit	2 (6)
Intuition	1 (3)		
Unitive	9 (25)	Universal unity	3 (9)
Noetic	8 (22)	Omniscience	5 (15)
*Exploring other worlds*	36 (100)	*Scene description*	14 (42)
Natural landscapes (e.g., river)	4 (11)	Natural scene	4 (12)
E.g. “Babylonian… Hyperdimensional garden”			
Plants and Flowers	10 (28)		
Infinite/Vast	n/a	Infinite	5 (15)
	0	Waiting queue	1 (3)
	0	Office	1 (3)
		Landscape	1 (3)
	0	*River*	3 (9)
		With lost souls	2 (6)
	0	City	1 (3)
“Spirit world”	1 (3)	“Heaven”	2 (6)
	0	“Hell” / Hellish realm	2 (6)
Laboratory	1 (3)	Operating clinic	1 (3)
	0	Cord of light	1 (3)
Molecular or subatomic	3 (8)	Atoms in walls	1 (3)
		*Darkness – Empty and Inescapable*	13 (38)
The Void / ‘Limbo’	4 (11)	Waiting room (no walls)	1 (3)
Dark space / void-like	2 (6)		
	0	Movement through (the darkness)	2 (6)
Sound (onset)	3 (8)	*Sound (associated with Movement)*	1 (3)
	0	Music	2 (6)
	0	“Shining black” (Dark–light simultaneity)	1 (3)
*Disembodiment*	19 (53) +	*OBE – Perception of body*	12 (35)
	0	Witnessing emergency situation [potential veridicality]	9 (27)
		Possibly corroborated	3 (9)
	0	Elevated perspective	6 (18)
	0	Sensed detachment	3 (9)
	0	Communicating in vain	2 (6)
Repulsed by body	1 (3)	Rejection of body	1 (3)
Etheric body	2 (6)	Etheric body	3 (9)
	0	Re-incorporation into body	2 (6)
		Incorporation into anothers body (attempt)	3 (9)
Sense of dying (onset)	6 (17)	Awareness of death	9 (27)
Sense of dying (during)	8 (22)		
Metaphysical beliefs (Inc. hyper-reality)	18 (53)	Real / Hyper-real	9 (27)
*Life review*	2 (6)	*Life Events*	8 (24)
Reviewing	2 (6)	Reviewing	5 (15)
	0	Reliving	2 (6)
Past	2 (6)	Past	6 (18)
	0	Future	1 (3)
Imposed	2 (6)	Imposed	2 (6)
	0	Selected/Free scrolling	1 (3)
	0	*Judgement*	
		Self-judgement	2 (6)
		By ‘Council’ / other beings	2 (6)
	0	Life as a ‘model’	1 (3)
		Cinema	1 (3)
		‘Over-brain’	1 (3)
Difficulty expressing / recourse to metaphor	19 (53)	Ineffability	4 (12)
	0	*Threshold/border*	3 (9)
		Doors	2 (6)
		Black screen	1 (3)
Loving or connected	10 (28)	Love	3 (9)
Benevolent/loving entities	10 (28)		
Co-creation/lucid dream-like	10 (28)	Co-creation / Lucid dream-like	3 (9)
*Deus Ex Machina*	3 (9)	‘*Deus Ex Machina*’ (From primordial/void-like to heavenly space)	2 (6)
Geometry or patterns (onset)	6 (17)	*Colourfulness*	
Geometry (during)	16 (44)	Patterned	1 (3)
Flux	13 (36)	Fluctuating	2 (6)
	0	*Return*	19 (56)
		Dark night	1 (3)
		*Told to go/sent back*	5 (15)
		Made decision	2 (6)
		Given choice	1 (3)
		Ejected	4 (12)
		Guided to body	2 (6)
		Brutal	3 (9)
		Sleep	2 (6)
		Confusion	2 (6)
		Disappointment to return to illness/life	12 (35)

The themes are ordered by highest frequency in NDE thematic analysis (excluding ‘Entry’ and ‘Return,’ which represent the beginning and ending).

Green highlight under NDE: Added to the original after the extension of the analysis; Green highlight under DMT: Added to the original analysis to match the NDE theme.

*Italic*: Not included in the “Near-Death Experience, Death & Birth” category of the current DMT analysis in the *Results* section below, yet aligned with themes in NDE analysis (Cassol et al., 2018). Therefore, these represent additional themes directly relevant to the near-death syndrome, although coded in previous reports (namely Michael et al., 2021, 2023a, 2024).

n/a: Uncoded or uncounted (not necessarily implying a lack of presence in the report).

0 (frequency): Not present in either DMT or NDE analysis.

+: Proportion only explicitly reporting theme may be higher (to be more consistent with NDE).

Uncoded, and thus excluded, yet not necessarily absent: ‘Emotional’ and ‘bodily’ experiences of NDE, and Entity ‘roles’ and ‘demeanours’ of NDE (due to their being uncoded in original analysis).

“” denote respondents’ own terminology.

Table 2 compares the themes and subthemes found in the analysis of DMT experiences (across both the current and previous reports; Michael et al., 2021, 2023a) with those identified in the extended analysis of NDE narratives (from Cassol et al., 2018) conducted in the current study using shared raw data.

Regarding the feature of a specific form of ‘light,’ while it remains consistent on the Near-Death Experience (NDE) scale (refer to the *Methods* for an explanation of this measure’s application and Supplementary material for the corresponding results), this theme appeared three times more frequently in NDEs compared to reports from DMT. Notably, the predominant inclination towards white light was completely absent in the DMT accounts. Therefore, the idea of being enveloped by a diffuse, non-dazzling brightness (of internal origin) resembles the content found in DMT experiences (though characterised by colorful and geometric elements; further *distinctions* in content are elaborated upon in the *Thematic Analysis of the DMT experience section* below). Additionally, relevant to this discussion is the ‘tunnel’ phenomenon. Although it is not included as an item on the NDE scale, narratives related to NDEs depicted (often dark) tunnels in 27% of instances, all featuring a light at the end, typically occurring near the start of the experience. This contrasts with DMT reports, where 19% of individuals noted encountering ‘tunnel-like structures’ at the onset, while 28% indicated these structures persisted throughout the experience. Despite the relatively similar prevalence rates, the resemblance in content to conventional ‘tunnels’ and their timing highlights significant differences when compared to NDEs.

Considering the ‘encounter phenomenon,’ the NDE scale scores indicate that ‘mystical beings’ were significantly more frequent in the DMT field study results than in NDEs—where only 44% of near-death narratives reported some encounter, compared to 100% of DMT participants. This discrepancy is further evident in the NDE’s theme of ‘imaginary’ beings, appearing in 27% of cases, while the equivalent ‘otherworldly creatures’ in the DMT report accounted for a much higher 72%. The idea of a ‘Being of light’ (benevolent, hyperintelligent, and so on) was similarly frequent (8% in DMT, 15% in NDE); however, the identified content varied significantly. Hostile entities (e.g., fearsome, demonic-like) were also minimally present (3–8% in DMT reports across various subtypes, 3–9% in NDE reports, including different subtypes)—both may underestimate this prevalence, suggesting that DMT subjects’ experiential contexts and home settings could influence their accounts, while distressing NDEs are likely underreported, estimated at around 14% (Cassol et al., 2019). The ‘trickster’ figure, embodying ambivalent and deceptive traits, was notably more common in DMT reports (8–14%) compared to NDEs (3%); however, the description of the trickster entity in NDEs closely resembled those found in DMT (as discussed in *Expressly NDE-like Cases*, below). Briefly, one DMT participant (*DD*) described encountering a god-like yet childlike being who lured him into another realm, simultaneously toying with him while appearing ‘maternal or nurturing.’ Similarly, a single NDE case featured a radiant black face urging the NDEr to join it in a realm of eternal suffering while also performing ‘psychic surgery’ benevolently (this was also reported by another DMT subject, *RV,* who described mantis-like beings ‘working on his jaw’ to alleviate trauma), with the experiencer referring to them as the ‘Holy Spirit.’ Thus, these two accounts reflect each other in content. The notable absence of ‘ferrymen’ in the DMT reports is striking, as the concept of such psychopomps may not be relevant in a context that is not genuinely near death.

The NDE scale item regarding meetings with the deceased received one of the lowest scores amongst DMT participants, while NDErs scored significantly higher. The thematic analyses reveal that the vast majority of NDE encounters involved human or human-like figures, most of whom were family or friends, with a notable portion being deceased (27%). This is similar to the DMT interviews, where, although a minority of encounters involved humans and only a few were known individuals, those known were also deceased (6%). The small fraction of known living persons appearing in NDEs (none in DMT) suggests that the argument regarding the ‘peak in Darien’ phenomenon—seeing individuals not known to be dead—being merely a matter of statistical chance is unlikely. Communication from the deceased consistently revolves around the theme of ‘returning’ to life (21%), highlighting their role in ‘sending back’ the experiencer, which is entirely absent from the DMT reports.

The concept of ‘ego dissolution’ is also absent from the NDES, which reflects its presence in only one NDE narrative—compared to possibly underreported occurrences in 32% of DMT trips. ‘Unity’, closely linked to this, is an NDES item that displays minor differences between the states; however, the theme of unitivity is nearly three times more prevalent in DMT (25%) compared to NDE (9%). On the NDE scale, there is no difference regarding the ‘separation from body’ item. Nevertheless, ‘OBEs occurred in 35% of NDE cases, with most individuals reporting a sense of witnessing their critical condition, half of whom hovered over the scene (with at least 9% claiming a *corroborated* perception). This contrasts with the DMT state, where (again, likely underestimated) 53% reported ‘disembodiment,’ specifically referring to a total loss of body awareness without any similar detachment or autoscopic phenomena. Thus, there are significant content discrepancies despite comparable prevalence. Similarly, attempts to reintegrate into a body different from their own were distinctly unique to the NDE.

Interestingly, the recognition of proximity to death is not listed as an item on the NDE scale. Moreover, an ‘awareness of dying’ is noted in only 27% of cases, placing it in the minority within the thematic analysis of NDEs. In contrast, the prevalence of a ‘sense of dying’ in the context of Dimethyltryptamine (DMT) varies from 17 to 22%, showing a noteworthy similarity. This indicates that DMT might encompass a significant aspect of NDEs, potentially influencing the experiential content through suggestion.

The occurrence of entering ‘other worlds’ was significantly higher on the NDES for DMT participants, supported by the thematic analysis, which revealed that 100% of participants experienced this, compared to the NDE descriptions of iconic imagery organised into scenes at only 42%. This likely reflects that many NDEs exhibit only shallow, initial features, in contrast to the high-dose DMT experiences characterised by ‘breakthrough’ intent. The appearance of ‘natural scenery’ (e.g., lush vistas), however, was quite similar between the two states (11, 12%), with the notion of it being an idealised mirror image of Earth (Shushan, 2018) common to both—although this often included fractal or hyperdimensional elements in DMT. A few everyday scenarios, such as queues or offices, were also notably absent from the DMT experiences. Hell-like environments were present in 6% of NDEs, featuring imagery such as rivers of damned souls, which were not seen in DMT experiences. Descriptions of ‘heaven’, on the other hand, appeared twice in the NDEs, while the concept of entering the realm of spirits was also powerfully expressed once by a DMT participant (*LG*).

The theme of being ‘between spaces’ (in limbo or waiting) was more prominent in the DMT reports, with a prevalence of 11% compared to 3% in the NDES. When characterised as a period of darkness, the DMT experiencer seemed to exist in a state of suspended animation, in contrast to the NDEs, where individuals progressed through this state towards the light. Although a blackness that emanated light was only observed once in the NDEs, it was absent from the DMT experiences; however, this phenomenon did occur in two instances involving changa, a DMT admixture, as documented in a separate report by Michael et al. (2024).

The occurrence of a ‘life review’ was significantly higher for NDEs on the NDE scale, happening only in one DMT participant on two occasions (6%) compared to 24% of NDEs noted in the thematic analysis. This conceptual and feeling-oriented DMT report contrasted with the explicit judgment made by oneself or accompanying entities, where one’s life was physically represented in the NDEs (e.g., like a film). However, such representation is observed in other changa accounts (Michael et al., 2024)—although the content was presented in a more DMT-like form of ‘code’ (again, this content is comparable to one idiosyncratic NDE mentioned here, where life was embodied in a sort of ‘brain-like’ structure).

A sense of profound love, sometimes unconditional, was surprisingly uncommon amongst the NDEs (9%), whereas this feeling was experienced by over a quarter of DMT reports (28%), typically as expressed by benevolent other beings. Similarly, in terms of prevalence, the ‘lucid-dream-like’ process where the experiencer could wilfully alter elements of their unfolding experience was rare in NDEs (9%) and significantly more common in DMT interviews (28%).

No DMT report has ever suggested a ‘point of no return,’ while such a threshold is noted in 9% of NDE narratives. Similarly, no sign of ‘returning’ to life appears in the NDES (despite its common occurrence), yet it is described by 56% of NDErs, compared to none of the DMT subjects whose experiences simply fade away. This again highlights the lack of distinct, functional messages from other entities in DMT about ‘sending back’ the experiencer, which seems to be essential only in the state when one approaches death.

#### Features not illustrated in Table 2

It is important to note that there are many other coded DMT themes detailing content not illustrated in Table 2. These themes are not represented, either because they are genuinely absent from the NDE narratives (reason 1) or because they may be present, but due to the NDE analysis (even when extended) being much less nuanced than the DMT analysis and the use of narratives rather than in-depth interviews (reason 2), the equivalent themes in the NDE experience may not have been originally coded. Supplementary Table S1 (*see* SM 3) outlines all these DMT themes not shown in Table 2, where the orange-highlighted themes in Supplementary Table S1 indicate the lack of coding (reason 2), and the remaining ones represent those truly absent from the NDE (reason 1). Thus, these highlighted themes in Supplementary Table S1 (SM 3) should not be fundamentally considered absent from the NDE repertoire, and many of them are sometimes evident across the NDE narratives provided herein. A narrative write-up of all these themes is available in the Supplementary material (*See* SM 10).

**Figure 3:**
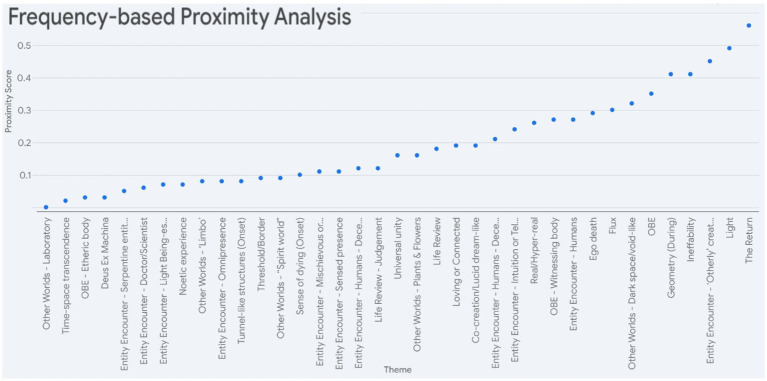
Frequency proximity plot based on the proportion of DMT interviews in which the theme emerged and the proportion of NDE narratives in which they appeared. The difference between the two (the proximity or distance) indicates the proximity score. This distance or proximity helps reveal which themes are closely shared between DMT and NDE (small distances) and which are distinct (large distances). The proximity score is on the y-axis, while the themes are on the x-axis. The themes are arranged in ascending order of proximity score, meaning the higher the point on the plot, the more dissimilar the prevalence of themes between DMT and NDE. This visualisation is comparable to that of Figure 3 by Martial et al. (2019), which graphically plotted similarity scores between various psychoactive substance experience reports and a corpus of NDE reports based on semantic analyses against a class of psychoactive substances. A tabular version of this, including proximity scores, is provided in Supplementary material (see SM 15). When there is a “//” in the theme name, the label before refers to its designation in the DMT analysis, while the one after corresponds to the name in the NDE analysis.

Figure 3 provides a graphic representation of most of the key themes shown in Table 2, making it easier to visualise the comparability between the two states, at least in terms of theme presence and prevalence.

### Thematic analysis of the DMT experience: “near-death experience, death, and birth” and qualitative comparison with NDE

The subsequent section constitutes the final component of the thematic and content analysis concerning the DMT experience derived from the field study on DMT utilisation. Table 3 delineates all levels of themes described, with the exception of the final subthemes pertaining to the ‘mystical experience’ and additional clarificatory notes, which are documented in Supplementary Table S2 within the Supplementary material (*refer to* SM 4). Additionally, a comprehensive graphical representation of the superordinate themes is available in the Supplementary material (refer to SM 5). The inaugural superordinate theme, designated as *Canonical NDE Themes,* encompasses at least one of its themes identified in a total of 34 out of 36 interviews, thereby accounting for 94% of all experiences documented. All emergent themes have been inductively coded based on the data collected. As previously stated at the beginning of the aforementioned *Analysis* section, the final phase of consolidating subthemes into this superordinate theme of canonical, namely more typical themes (hence distinguishing them from *Less Typical NDE Motifs, as elaborated below*), was directed by their prevalence on the most recent and standardised measure for evaluating NDEs—the NDE-Content Scale. In what follows, the DMT themes will be meticulously delineated, followed by a comparative analysis of the specific qualitative content in relation to the NDE narrative data. For the *Canonical themes,* this analysis is categorised into ‘Similar content’ and ‘Different content’. The excerpts utilised are frequently extensive, thereby rendering the analysis more idiographic, which is essential for this study’s objective of facilitating the comparison of not only phenomenology but also subjectively variable content.

**Table 3:** Thematic analysis of the DMT experience: an encounter with the self; a tabulation of categories, superordinate themes, and subthemes explored in this article.

Encounter with death	No. interviews /36 (%)
Near-death experience, death & birth (category)	
*Canonical NDE features (superordinate)*	*34 (94)*
Disembodiment (subtheme)	19 (53)
Translocation elsewhere	12 (33)
Tunnel-like structures (during)	10 (28)
Bright light(s)	9 (25)
Sense of dying (during)	8 (22)
Tunnel-like structures (at onset)	7 (19)
Sense of dying (at onset)	6 (17)
‘Limbo-land’ / The Void	4 (11)
Light Being-esque	3 (8)
Deceased family	2 (6)
Hyper-empathy	2 (6)
Life-review-like	2 (6)
*Less typical motifs*	*14 (39)*
Birth imagery or being born	5 (14)
Death imagery and skulls	5 (14)
Sounds (at onset)	3 (8)
Reduced fear of death (after-effect)	3 (8)
Etheric body	2 (6)
Being in prime	2 (6)
Partner lying dead	2 (6)
Dark and earthy space	1 (3)
‘Placebo-death experience’	1 (3)
Psyching the psychopomps	1 (3)
Scenes on screens	1 (3)
Repulsed by body	1 (3)
The mystical experience	
Typical mystical dimensions	29 (81)
Entheogenesis	3 (8)

*See Supplementary Table S2 for a complete list of all subthemes related to the Mystical Experience.*

#### Canonical NDE themes

##### Similar content

###### Bright light[s]

A vision of bright light (or lights), especially very intense and usually colourless, was reported by 9 out of 36 participants (25%). *LR* here describes:

“…this really bright light – it just got really bright, OMG. I’ve smoked DMT a few times, but not in a bright light like that. It was so bright that the colours were melding into whiteness. It was almost like someone was shining a torch in my eye but without the pain.”

*GR* describes it similarly, but most importantly, at the very beginning of his experience, in the context of darkness, he passes through it to enter the subsequent phases of his journey (which also mirrors perinatal regressive imagery; see *less typical NDE motifs* below):

*“*Just darkness, then a bit of light. Literally, as if I’m, you know, putting my hands in the dark space and just like ripping it a part… or you know coming out of the egg… Or the womb!… Just like *swoosh* through it.”

The feature of ‘light,’ which can be intense and appealing, appeared in 25 out of 34 NDE narratives (74%).

###### Sense of dying (*at onset* and *during*)

The very sense of dying, subtly distinct from the belief of actually being dead, was reported in only one case (*LG*). This experience was described by six participants (17%) *at the onset* (when it is typically articulated by classic NDErs) and by eight participants (22%) as persisting *during the unfolding* of the trip. In his second experience, RH shares its challenging nature (which is not the norm in near-death studies), accompanied by the fracturing of his sense of self; ego death is much less common across NDEs (see *Quantitative comparison* above).

“Dying at the beginning; pain, confusion… I seemed to be in what felt like death really, really quickly this time. It was quite horrifying. But…this time, it was only a few moments. It started with softly opening up patterns, like very beautifully coloured blankets, but then all of a sudden, it went into the death bit.

*Interviewer: So it was really like piercing through the veil?*

Yes, exactly like that… The dying bit is the worst bit of not knowing. That’s when I do not know who I am.”

In comparison, the ‘awareness of being dead’ was reported in 9 out of 34 NDE narratives (27%).

###### The void / ‘Limbo’

Once again, in conjunction with his sensation of impending demise and the concept of ego death, *RH (2)* elaborates on an encounter characterised by a void-like dimension, often referred to as “limbo-land,” as noted in four interviews (11%). In this context, he describes a daunting, unavoidable, and content-less scenario filled solely with distorted feelings of inversion. The theme of *translocation,* defined as the sensation of moving from one’s original position to a completely different space, is reported by 12 individuals (33%), often described as a “sucking” or “magnetisation.”

*“*Oh, it’s horrifying, terrifying, horrible… You know, stuck in a place that I just do not want to be in… a feeling of being sucked was in there as well…there was an inevitability about it which seemed really important. I knew there was no way out of it… Terrible powerlessness…I do not matter anymore… It’s always got a jagged, fragmented quality…its already broken… And that always happens… it’s not here and not there. It’s absolutely limbo-land… It’s just not a place I know… Nothing makes sense.”

In 13 out of 34 NDE narratives (38%), reported noted empty, inescapable darkness, with one case, representing 3%, described as a ‘waiting room.’

Despite the above—Dying, Light, and Void—significantly echoing the experiences described by classic NDErs, they cannot be easily conceptualised through the two dimensions of structure and content. The themes themselves offer little, if any, scope for variability and subjective manifestation and thus can only be said to reflect NDEs through their phenomenological structure rather than their qualitative content, which would otherwise make them more impressively similar to NDEs.

##### Different content

In addition to the aforementioned themes, the remaining themes within the category of NDEs analysed here were generally limited in content variation but showed clear differences in that content.

###### Disembodiment

The evidence for this phenomenon can be observed in the theme of disembodiment, as described overtly by 19 participants, which constitutes more than half of the sample (53%). This effect is manifested in numerous excerpts utilised in this analysis. Notable examples include expressions such as “completely dissolved” (*AN, AF, FF*), “merely pure thought, devoid of bodily sensation” (*LR*), and “merging of my body with the scene” (*MP*). This stands in stark contrast to the traditional account of ‘out-of-body experiences’ (OBEs) that occur at the onset of NDEs, evidenced in 12 narratives (35%) where individuals report a sense of their consciousness detaching from their physical form and relocating elsewhere. In all instances, this experience is accompanied by observations of one’s own body (‘autoscopy’). *The* DMT experience described by EM, categorised under *expressly NDE-like cases* outlined below and presented in its entirety in the Supplementary material (*Refer to* SM 6), serves as an especially illustrative example of this critical distinction.

Patient CG_fmp80 (from the NDE narratives) provides a notable example. During a cardiac arrest at home in 1990, he describes finding himself “outside my body…3–4 metres from the head” in the form of a “small cloud, a vapor.” He reports allegedly veridical perceptions (where 27% of cases report observing the scene and 9% include possibly corroboratable elements), which may be considered verifiable. He recalls seeing his son crying and shouting nearby and states, “through the walls of the house, I perceive my wife, who is outside (the house)…impatient with the arrival of the help.” He also claims to have witnessed a “spider in its web in a top corner of the room” and describes being “pushed back in the corner where the spider is” when he was loaded onto a stretcher. He realised that, upon initially seeing the spider, “this corner was hidden from me by a bay that separates the room into two, and I could not (have otherwise) seen it.” During a later arterial tear during a coronography examination in June 1995, he reports experiencing another noteworthy OBE:

“When I wake up, I see my four-legged teacher on top of me, performing cardiac resuscitation… There is the anaesthetist, my treating physician, a surgeon, the teacher, nurses and a lady who is not very active at the moment and speaks a lot. In a vulgar voice, she says what seems to me to be nonsense about the situation everyone’s going through. Some laugh, others are unmoved. My GP, who is also a friend, is rather serious. In fact, they just lost me!… for quite some time, I’ve been in my “cloud” and watching all this little world that is busy around my body… I perceive their feeling of failure, even panic. I feel calm and peaceful. I see through the bodies, my own body open in two, exposed there in a blinding white and raw light.”

###### Tunnel-like structures (*at onset* and *during*)

Seven volunteers reported experiences related to tunnels, or more accurately, structures or imagery that evoke the concept of a tunnel, *at the onset* (19%)—and 10 *during* the experience (28%). These tunnels, which initiated the trip, tended to remain a feature throughout (notably, this is not something typically seen in actual near-death experiences). For example, *LR’s* idea of ‘portals’ was mentioned multiple times during his DMT journey:

“This person…walked in front of me, then he opened, he had his hand [on] this portal, this bit in my mind, he opened it up…just opened this portal, and I was flying through these dimensions…the guy came back the second time…he grabbed one of these points and kind of pulled it. Then, it opened this portal.”

*AF* describes approaching a tunnel in quintessential NDE fashion—though once more, in the context of “female entities,” who, as she notes, were “inviting me…(to) this sensual game of shapes and colours… flirting with me. Their bodies were changing shape” (manifestly absent in NDEs):

“There was a tunnel, yellow, pink and blue, and there were curves [in it], and at the end of the tunnel, there was the usual geometrical shape I always see… I was moving towards the geometric multi-dimensional cube, and the entities were telling me to enjoy and to play with them going towards the cube.”

Notably, the attributes of a ‘geometric hypercube’ and the guidance offered by whimsical feminine entities associated with the tunnel phenomenon were acknowledged by at least one additional individual, identified as *DD.*

“So the cuboid, splintered, fractal…tunnel-type thing… She started off as 2D, then there was definitely the face, and…was drawing me through it… So she is beckoning me backwards through this cuboid tunnel-type vortex thing [laughter]!”

Regarding the NDE narratives, a ‘tunnel’ (and, in one case, a corridor) leading to light was reported in 9 (27%) cases. Similarly, a ‘dark tunnel’ leading towards light was identified in 3 (9%) cases. The NDE of *FR79* exemplified the light at the end of a classic-looking tunnel at the onset of her experience:

“Like all ‘experiencers’ who have been through this kind of vision, I see the big light this time… a circular light giving a tunnel feel because of the darker, circular centre too. The whole thing seemed alive and very powerful but not blinding. There was no heat. Without suction or projection, I crossed the tunnel at full speed. Speed is not the right word because there was no movement. The impression looked more like a dissolution of my person and an equally sudden hatching. A lightning crossing of sorts.”

###### Light being-esque entities

An encounter with an entity resembling the so-called ‘being of light’ was reported by 3 DMT participants (8%); however, there were significant deviations from the classical description. This is most evident in *RH*’s second trip (his first was more typical; see *Expressly NDE-like cases* below).

“Extraordinarily beautiful. I do not know if I cried, but the intensity of it was just amazing,… Fascinating how I knew I was separate, I knew this wasn’t me, and I wasn’t a part of it – and yet…we knew each other!… this was a familiar thing somehow… Without any doubt…extraordinary intelligence, and I do not mean sophisticated technology. I just mean beyond anything I can imagine, really… Just a profound sense of love, the most profound sense of love… He knows…he knows… Wonder, shock, profundity… I’m not curious. I’m just with it, a real gentleness that it had… the speed seems to be something like that, not more than that [gesturing gracefully]…if you told me it was made of 1,000 beings I’d believe you,…even though it only appears to be one thing, it’s almost like its *consciousnesses*… like a [hive mind]… It’s so massively intelligent I’m struggling to find words for it, or it’s like a conglomerate.”

The following explanation highlights the stark deviation in content from classic NDEs:

“…almost tentacled…The closest I can get to it is either like my imagining of what synapses look like, synaptic nerves… a bit octopussy? But that would just make it very-… an entity which certainly had quite a lot of things reaching out all over the place.”

In this way, the being illustrated shares the shared attributes of beauty, benevolence, familiarity, hyper-intelligence, profundity, and grace, thereby emanating a sense of sacredness (*refer* to SM 1, ‘The Mystical Experience’). However, it is evidently of a distinctly alien nature compared to traditional ‘beings of light’.

Amongst the various narratives of NDEs, 5 (15%) reported encounters with beings of radiant light. *Patient 73* describes observing “supernatural beings” dressed in “shiny white gowns,” which he perceived as reminiscent of doctors. Conversely, BJ_fmp319 recounts *an experience in which* he waited in “a queue… somewhat like that of a supermarket” with several individuals (presumably other deceased individuals) positioned before an ethereal “counter… that appears to emanate a resplendent light,” leading to a remarkable encounter.

“a gigantic, white, totally white guy: whiteness itself, resplendent with almost white light alone, a being of light (no, whiteness). He is so surrounded and drowned in white that his face has no colour. He is very imposing (like Barry White!), huge, strong, he wears a long white beard, long white hair, ageless… He does not seem aggressive or unsympathetic… he rather gives a reassuring and benevolent impression… Obviously, he’s directing or deciding something… He knows me from my record. I can see an expression of kindness on his…almost paternal face. In a tone without appeal…I hear his voice inside me… “Sorry, it’s not your day!”

Consequently, this being exhibits all the characteristics of an anthropomorphic light entity yet lacks any qualities typical of science fiction. It performs the role of guiding the experiencer back in a notably clerical manner. Nevertheless, one DMT participant, referred to as *AV,* briefly depicts a vision of preternatural light as though it were an omnipresent consciousness, reminiscent of common NDEs. However, it is once again described in an unconventional way as a mandala. Notably, the Tibetan Buddhist *text, the* Bardo Thodol, describes entities in the *Union* phase of *Dharmata* after death as appearing as luminous mandalas (Rinpoche, 1992).

“I was drawn to the centre of this mandala… The yellow and the white colour was very strong… I never had a DMT journey without the entities. There were no entities there. Woah!… It was like being *inside* the entity… the rays, the yellow, white, like inside the sun… Like pure light… It was a white core… It was spinning.”

###### Deceased family

The encounter with the deceased was reported by only 2 DMT participants (6%), where both instances notably involved known individuals, specifically family members. However, *SP’*s example was extremely fleeting and ambiguous, while *JM*’s was unmistakable. Naturally, *JM’s* presentation was also unconventional for NDE accounts, referencing framed “magic mirrors” and, more significantly, the humour within a broader context of elf-like (mercurial and mischievous) entities that exhibit a “dismissal of death.” He further emphasises the unusual emergence of this deeply personal content, which resembles NDEs more closely and feels out of place compared to his earlier DMT trips.

“My mum popped up, which was weird. She’s dead… I’ve never had such a direct link to something personal before, so that was interesting. But it is done again in a comical way; there’s a lot of comic stuff in there! A lot of distraction… She was in a frame, a bit like a magic mirror, like Snow White or whatever… And she had no hair…because she had cancer… And she was quite happy, again everyone was in a good mood, for whatever reason! It was quite jovial. There were maybe hints that it could get dark, but there was a kind of impression, *Oh, do not worry about that [the mother]; that’s not your concern.*”

Amongst the narratives of NDEs, 8 (24%) included experiences involving the deceased. *Patient 26* first described the following, consistent with distressing NDEs, where elements such as monstrous entities are readily observable within the DMT universe:

“The whole background was black… I also saw shapes, human-looking but without heads… The whole atmosphere was very scary… I was on a kind of canoe, which followed the current of a very black river. I was on my way to a bridge, where the headless shapes stood. They were pulling other people from the canoes that passed under them. They ripped their nails off and tortured them.”

Suddenly, an ancestor of his appeared, and while also reflecting the *Deus Ex Machina* motif present in the current DMT analysis *(AN, RH)*, the ancestor functioned as in classic NDEs to send the patient back from a treacherous place to a beatific one:

“I saw behind me my deceased grandfather…illuminated by a kind of light which I could not see the origin of. He said, ‘There is nothing here for you, kid!’… I found myself at the top of a hill, overlooking…forests of fir trees and flowers. I felt an indescribable wind filled with happiness. The sky was full of beautiful colours, which I had never seen before… There was no suffering.”

###### Life review-like

The reanimation of experiences or emotions from an individual’s personal history, akin to a ‘life review’, was reported in two DMT interviews (6%), both attributed to a single participant, referred to as SP. During his initial session, he provides the following account:

*“*I was seeing faces of loved ones mainly and feelings for people. But it was very worldly, not…weird crazy things… there was a feeling of universal – it was lots of love, and it was like the absence of fear…a pure loving experience… this feeling of being aware of my own consciousness…it was about me, and I guess everything that connects through me, or I’m aware of… More like a narrative, like *Life*, *Voom*, all your experiences! So it was really fast… it felt alive and very real. Was it like dying? I guess!… Like a round circle of all the ‘stuff’…it was bowl-like.”

Eight NDE narratives (24%) involved reviewing or reliving ‘life events.’ While the feeling of re-experiencing one’s life, as described above, is replicable across authentic NDEs, even in this DMT account, the content differs from that of the NDE narratives—where entire life scenes are vividly relived with greater tangibility, often accompanied by a guiding presence or at least a sense of evaluation.

For example, patient *BJ_fmp319* first reports hovering over his own surgery, witnessing his open cranium and the medical devices around him until he is “caught in a lightning whirlwind of images” that he had forgotten yet remain sharply vivid, where he “can precisely choose to stop” at a scene as if it were “in the cinema… a movie summary,” concluding with the painful separation from his wife and tearful daughter, which he felt was “related to my deep sense of guilt, and that I am not very proud of my conduct.”

*Patient73*, before the sunlit grace, had supernatural-looking, doctor-like people build and then dismantle his life, which may reflect the peculiar tones of DMT imagery:

“They showed me passages from my life, from my childhood to my present age, projecting the scenes in front of me on a kind of table. It felt a bit like a view from above as if I were looking at a model. For example, I saw the company where I worked, its roofs, the trucks coming in and out, all in every detail. But they showed me a lot more negative than positive scenes. Each time, they pointed out my mistakes and my bad decisions to me and suddenly destroyed the model. They seemed to mean, “Did you see him [i.e. myself]? Well, you will not see him again!” It was unbearable, like a judgement, but with the punishment of having my life swept away with every mistake I had made… I felt the consequences of my bad decisions very strongly, and I felt worse and worse as the scenes went on. Then they tried to forgive me and make me come back into my body…

After acknowledging his death and experiencing a sudden absence of pain, *JYB16* reports the following: an intelligent ‘brain-like structure’ encoding his own, or life in general, was encountered (which may again mirror the stranger of DMT motifs).

“A universe that has no walls and where there is no question of gravity…dark metallic grey tones, as if to infinity. A wonderful sensation occurs when a kind of *Over-brain* comes over my head, like a hood of exceptional intelligence. This intelligence is universal or global… I find before me – my life. I look at this thing in 3D; that is my life, and that does not ‘unfold’; time is ‘integrated’ into it, and it is not linear. Everything in this life is visible, and this “global” intelligence allows us to read it, to conceive it… my intelligence allows me to look at my experience and draw conclusions… The real truth is there, unavoidable. The “last judgement” is done to oneself…: 1] I was a good man. 2] My life was useless. I did not do what I had to do…

In front of my life… it was…enlightened by herself [the intelligence], by her “existence.” Its oblong shape resembles that of a brain seen from above with creased waves. All I had to do was change my perspective to see another part of this life… And it seemed possible to choose a moment or character to integrate myself into it… But I had to first take stock… Only one image comes back to me sometimes. I was a little child in shorts and “illuminated” like a pure being. I was appalled that I had finally done nothing with this life. At least, it was not what I had come to earth for. Sometimes, I have the idea that I was summoned for this observation and only for him. So as to understand the signs that will be sent to me later.”

Another point of distinction between DMT and NDE states, particularly regarding life review, is the emphasis in the aforementioned NDE excerpts on regret for not using one’s life wisely, feelings of guilt, or evaluations made by accompanying beings. This reflects an existential burden related to the values of free will, personal responsibility, and the performance of positive actions—where the judgment is primarily *self*-derived or facilitated by ultimately forgiving entities, aligning with a framework of humanistic, compassionate non-judgment. This process is particularly well illustrated in the second NDE extract mentioned above and resonates with Zaleski’s (1987) noted progression from medieval near-death narratives to contemporary accounts, where a viscerally judgmental and hellish experience transforms into a rehabilitative and educational one, reflecting societal structural changes over time. This can be contrasted with many DMT experiences, which are fundamentally ‘playful’, as illustrated by the term ‘ludibund’ (from the Latin ‘ludere’ meaning ‘to play’) (St John, 2018)—especially regarding the sentiments conveyed in the *cosmic game* communication by DMT entities (Michael et al., 2021), notably the reports of *AF* and *SH*. While *SH’s* experience is not a life *review* per se, it still evokes an evaluation of meaning, values, and actions. In contrast to the above NDE example, it depicts a flirtatious female entity playfully inviting her to embrace the message that the universe is meant to be ‘yours to do as you wish’, enjoyed with light-heartedness, free from objective rules or scrutiny. This experience is included in the Supplementary material (*see* SM 9).

This suggests a dissonance between a moral and an amoral cosmology concerning the states, where the former, regarding NDEs, was enshrined in the experience that inspired Moody to write *Life After Life. In* this work, George Richie was asked by a being of light during his review, “What have you done with your life?” which he later interpreted as, “How much unconditional love have you given to others?” (Ritchie and Sherrill, 1978). However, somewhat countering this, the DMT trip can sometimes carry a sense of mission or exhortations to love (and even to educate) oneself and others (Michael et al., 2021; Davis et al., 2020). Finally, an excerpt from the *Epic of Gilgamesh,* potentially a documentary NDE (Shushan, 2009), is strikingly reminiscent of the encounters of *SH* and *AF and* is also included in SM 9.

###### Hyper-empathy

A form of ‘hyper-empathic’ experience, in which one feels the states of those known in life and the nature of their relationships, was also deeply intertwined with *SP’s* two life reviews (again, present only in his experiences). This is evident in his description above, particularly the final phrase, but is further explained here:

*“*I was going through the connections between, I do not know, my world and the people inside it, and it was beautiful… this feeling of wholeness, oneness… I went into the experience basically saying – giving myself to the universe… it was like a mirror of love” [*SP, 2*].

“It was this feeling like we were a collective organism… this thing about all humanity, human consciousness… it felt like I was literally experiencing loads of other people’s consciousness…this feeling of literally just it all coming through, like not my own sadness, [but] other stuff, and just letting it run through you… I remember looking at you and this idea of basically being able to look at myself, like I was looking at myself through you!” [*SP, 1*].

While previous literature has emphasised this special aspect of the life review, it was not as clearly evident throughout the NDE narrative data. However, it is still somewhat invoked by the feelings of empathy expressed by the above NDErs regarding how their behaviour adversely affected their loved ones.

### Less typical NDE motifs

Some themes under the final superordinate theme of *less typical NDE motifs* have already been described in the analyses above, with 14 out of 36 (39%) participants expressing at least one of these motifs. These motifs may be significantly related to the concepts of death (or birth) but are, by definition, not identifiable through new standardised measures of the NDE. This contrasts with the first superordinate theme (*canonical NDE themes*), whose features were grouped based on their presence in this scale. When comparing each motif in terms of content to the raw narratives from the qualitative analysis of the NDE (Cassol et al., 2018), only a few comparisons are discussed below. These include ‘birth imagery,’ the ‘etheric body,’ and ‘sounds,’ as they are the only three that are partially reflected in the NDE analysis. Regarding experiential content, they appear distinct from the accounts of the NDE analysis, except for the ‘etheric body.’ The quotations illustrating each of these DMT experiences and NDE narratives are included in the Supplementary material (*see* SM 13).

#### Birth imagery (or being born)

Some perinatal imagery or feelings of being *in utero* or rebirth—not in the generic sense (e.g., shamanic) as a literal or metaphorical transition from darkness to light—were evident in 5 out of 36 DMT instances. We have already mentioned *EM*’s state of being “between birth and death,” as well as *GR*’s emerging light from the dark, which resembles “a womb.” Two other instances also reference the womb, such as *ZD’*s statement: “I felt like I got sucked down into something… almost like being in the womb of something else,” but further qualifies this by saying, “like dropping into this round-bottomed and narrow-necked vase,” thus also associating it with a tunnel-like feature—which aligns with the perinatal regressive theories of NDEs (e.g., Grof, 1985).

The only reference to a phenomenon of this kind in the NDE narratives involves the patient *labelled* BJ_fmp319. However, within the context of a life review, this narrative mainly focuses on the experiencer’s actual birth, which is clearly different from the more symbolic depictions found in the DMT subjects.

#### Death/skeletal imagery

The same number of interviews—five in total—incorporated some explicit references to symbols or concepts that invoke the idea of death. These included *JM*’s dalliance with trickster-like figures, alluding to skull imagery in a comedic atmosphere while creating an intense sense of mystery.

#### Sounds (*at onset*)

We previously noted *JB*’s “rushing, breaking through, noise” initiating the DMT experience, expressed by people in total, including *GR* stating a “buzzing and ringing in the ears.” Compared to NDE narratives, this sound was reported by authentic NDErs only once and was linked to movement through a tunnel, similar to *JB’s experience.* While this suggests that the sound feature is quite rare in NDEs, it is also uncommon in the current DMT analysis; thus, in this regard, the two experiences may be considered consistent. However, the sounds in the NDE were described as “machine noises” and were actually related to hospital equipment.

#### Etheric body

Once again, *LG*’s simulacrum of his physical body in an “energetic form” has been noted above, mentioned by only two individuals in total, the other being *MP’s* description of his state as “out of this body…but in an etheric body.” The NDE narratives present three instances of an etheric body, which is also quite rare compared to the current DMT interviews. Very reminiscent of *LG’*s experience of being able to see his semi-transparent body from a third-person perspective after observing the scene of his own body’s “death,” NDE experiencer *FR79* appears to describe her translucent body from a detached viewpoint in addition to her original body.

#### Partner lying dead

A subset of participants also took part in an experiment in which two of them experienced the event together. *MS* reports having a vision of her partner appearing dead, recorded in two instances out of the 11 cases of partnered experiences. She further illustrates a kaleidoscopic display of skulls, again emphasising the theme of death imagery.

While it is very common to see the deceased in NDEs and uncommon to see the living, it has never been observed that the living—both those in your surroundings and those sharing the same experience *as the* deceased—are present. Given this, these partnered DMT reports are somewhat similar to cases of ‘shared’ or ‘empathic death experiences’, where a healthy person close to someone who is dying reports participating in the dying person’s experience (Shared Crossing Research Initiative (SCRI), 2021), or where multiple individuals near death come back to recount witnessing others in their experience (who ultimately die).

#### ‘Psyching the psychopomps’

This final theme is referred to as ‘Psyching the Psychopomps’ (psychopomps are supernatural agents tasked with guiding the souls of the dead to the afterlife) because a participant, *LG,* suggested during a conversation with the interviewer a more literal or transcendental interpretation of the experience. Specifically, DMT may allow the experiencer to enter the realm of the dead; however, upon arrival, the entities present there are either confused or aware of some sort of mistake (i.e., they expect those who have died, not individuals undergoing a drug-induced near-death-like experience).

Another resonant experience related to this one is reported by *MP* during a journey that occurred not during the current field study of DMT use but in a separate laboratory study of I.V. DMT while undergoing fMRI neuroimaging. He describes the emergence of benevolent guides, acting as ferrymen, who conveyed (via ticker tape) that he is immortal. They exhibited a panicked state out of concern for *MP*’s safety, fearing that, to have entered this space, he must be dying. He ensured that he would act as the reassuring presence for them, ironically, assuring them that his life was not in danger (*MP, personal communication, October 6, 2021*). However, this may be due to suggestion, where, despite the beings’ anxiety about the unfamiliar fMRI machine and hospital environment in which they found him, it may instead reflect *MP*’s own priming of potentially being in a vulnerable state surrounded by researchers concerned for his welfare. Nevertheless, the present authors were also contacted by another DMT experiencer, who described her experience in which “the praying mantis appeared, spoke telepathically with her in a way that she could hear saying, “Is she dead? No, she’s just on DMT,’ before proceeding to “perform some operations on her body to retune things” (Luke, *personal communication*, April 26, 2021).

Similar to both these experiences and their interpretations is the quintessential NDE theme of ‘the return,’ where NDErs often feel compelled to return, as if the experiencer is ‘not supposed to be here or to be dead.’ The classic message communicated upon return is a variation of ‘It is not your time/Go back,’ as noted in 7 out of 34 NDE cases (see Table 2). In this context, *LG* and *MP* are not ‘sent back’ *in the* traditional sense—a theme noticeably absent from DMT trips, as established in the earlier section on *content comparison* (although see Davis et al., 2020, where 4% received a reprimand, sometimes involving being sent back)—yet there remains a sense that something is amiss, evident in the demeanour and intentions of the entities. Similarly, the experiencer is not ‘truly dead.’ This misunderstanding may again be compared to the return being attributed to a ‘clerical error,’ often reported in Indian and other Eastern NDEs (Pasricha and Stevenson, 1986). Naturally, the distinction is that the DMT experiencer has intentionally directed their emergence into this space through an exogenous drug, as opposed to the spontaneous state of nearly dying.

### Expressly NDE-like cases

In referencing the Supplementary material, several *Canonical NDE themes* and their content are outlined based on a small selection of participants whose overall experiences seemed to align more closely with NDEs compared to others (*see* SM 6). Large portions of the complete interviews for two participants, *LG* and *EM*, are included, along with a follow-up communication from *LG* to the author, PM, which provides valuable insights into his experience. This consistency with NDEs suggests a higher prevalence of NDE-typical themes; at times, their content may exhibit more NDE-like qualities or the sequence of events may reflect the typical syndrome (or a combination of both). Therefore, the nuances of the qualitative content remain, almost always, stereotypically psychedelic *or* DMT-like, *particularly* for LG and EM. A brief summary of the themes specific to these two participants, covering both the *Canonical NDE themes* and some *less typical motifs* along with additional NDE-related content, includes the following: *LG*’s DMT experience featured a sense of dying, disembodiment, translocation elsewhere, an etheric body (associated with levitation and autoscopy), a tunnel-like structure, reassuring entities, and grief over his ‘death.’ *EM’*s experience involved a sense of dying, a sense of familiarity, disembodiment, tunnel-like structures, benign and protective entities, a natural landscape, and a light with no source.

The content, however (*see* SM 6 for full reports), regarding the themes mentioned in both trips significantly differed from actual NDEs in several ways: *LG*’s hallucination of a cardiac arrest scene (which was not objective), a holographic and vertical icicle shape (which moved, unlike him as he passed through it), and alien-like faces of entities (who seemed confused about his presence in the ‘spirit world’ after using a drug). In terms of sequence, the feeling of moving from the emergency scene to a tunnel—a representation of a sort of preternatural world where the entity encounter took place—occurred essentially simultaneously. This contrasts with deeper NDEs, where the tunnel serves as a distinct transition before a ‘breakthrough’ into the ‘otherworldly’ stages. As for *EM,* she claimed her sensation of dying stemmed solely from feeling poisoned by the DMT. She explicitly stated that her disembodiment was not like an OBE. The tunnels resembled barber-shop spirals, moving in a way similar to *LG’*s, which also persisted throughout the trip. The entities appeared as dancing, body-suited harlequins, and the garden was intricately decorated with mosque-like motifs. She also reported creating a three-dimensional hexagon (which she claimed was mathematically impossible) during her vision—such complex (even ordinary) geometric shapes are atypical of NDEs but are part of DMT lore. The sequence of her experiences was somewhat more consistent with NDEs, progressing from disembodiment to tunnels and the encounter, eventually reaching a garden of light—though, like *LG,* the tunnels were always present, again blurring the common NDE motif of the tunnel breakthrough to elsewhere.

### The mystical experience

A brief continuation of the thematic analysis, specifically focusing on the classical mystical components of the DMT experience, can be found in the Supplementary material (*See* SM 1). This is included in the SM because these themes do not directly contribute to the discussion of DMT-NDE parity, as the core mystical state, arguably by definition, is ‘without’ content. Accordingly, similar to the previous themes of Bright light(s) or the Void being structural elements with minimal content variability, it is not easy to distinguish between the DMT and NDE experiences, as it is understood that such mystical themes are identical for both. This is supported by the finding that responses to the mysticism scale and to the NDES resulted in a significant convergence of the two supposedly separate mystical and NDE constructs, with only three features preferentially loading onto the NDE factor (Greyson, 2014). In this context, the mystical components could be said to drive, or even overemphasise, much of the claimed similarity between the two types of experiences.

## Discussion

### Summary of main results

The *results* of the abstract succinctly summarise the major findings. A total of 94% of participants reported experiencing at least one of the ten ‘canonical NDE themes,’ although at least two DMT participants indicated a relatively higher number. ‘Less typical NDE motifs’ were also noted, including birth imagery, death/skeletal imagery, sounds (at onset), the etheric body, a partner lying dead, and psyching the psychopomps. Other classical NDE features, such as light at the end of the tunnel, out-of-body experiences (OBEs), and dramatised returns, were entirely absent, with deceased individuals and life reviews being virtually non-existent in DMT, while many DMT features were lacking in NDE. DMT clearly shares a more fundamental phenomenological structure with NDEs but shows differences in prevalence; for instance, DMT is considerably more generative of breakthroughs to typically more fantastical other worlds, encounters with beings of non-human/non-animal nature, and experiences of heightened ego dissolution/unitive states, while the sense of dying was comparable. The small thematic cluster of ‘dying,’ the ‘light,’ and the ‘void’ was relatively content-free and thus showed no differences.

### Summary of similarities and differences

Considering the triangulation of approaches to the question of the DMT-NDE comparison—specifically neurobiological, quantitative, and qualitative—this study mainly focuses on the latter two methods. Initially, the results from the NDE scale indicated that all DMT experiences in this study are considered to ‘qualify’ as near-death experiences. Furthermore, based on the frequency analysis of themes, it is clear that all DMT reports exhibit numerous individual characteristics of near-death experiences. This highlights that DMT can replicate most of the components associated with the structural phenomenology of near-death experiences (e.g., Timmermann et al., 2018).

However, quantitative methodologies, especially thematic analysis, have shown that certain features are significantly less inducible by DMT and primarily characteristic of near-death experiences. These features include encounters with deceased individuals, life reviews, and the threshold of no return. Such features could be considered emblematic of the near-death experience. Notably, the threshold feature scored significantly higher amongst near-death experiencers, according to Timmermann et al. (2018) prior to the conservative Bonferroni correction and was completely absent in the thematic analysis of DMT.

Conversely, several aspects are indeed more readily induced by DMT, suggesting they may define this substance more clearly. These include encounters with other entities, whether human or non-human and the experiences of entering alternate realms or the dissolution of the ego, which is central to classical mystical experiences. Consequently, these specific features distinctly characterise DMT and elements associated with near-death experiences, implying that they may serve as primary contributors to the overlap between DMT and near-death experiences.

Moreover, approximately one-fifth to one-quarter of individuals who have experienced DMT and near-death experiences reported feelings associated with dying in the thematic analysis—a finding that may be unexpectedly low for near-death experiences or relatively high for DMT. Finally, phenomena such as preternatural light, the tunnel effect, and disembodiment were reported more frequently, though not significantly, in near-death experiences compared to DMT, correlating with the most common initial stages of near-death experiences.

Regarding the primary focus of the current study—the qualitative content analyses, as well as the theme frequency analyses—the final conclusions indicate that nearly all content of the DMT reports at this subtle level did not reflect typical NDEs; instead, it was characteristically ‘DMT-like’. This concept is almost perfectly illustrated by the quote at the beginning of this article, which recounts Watts’ own DMT experience, showcasing the structural component of the tunnel phenomenon (common in NDEs) yet remodelled with the DMT-like finesse described in this article’s findings.

In short, content unique to DMT (and not NDEs) can be distilled into bizarre—though distinct from the bizarreness of REM dreams, which are hyper-associative and often nonsensical—kaleidoscopic, extraterrestrial, transcultural (i.e., not limited to one’s own culture), and non-biographical themes. The more artificial, sometimes infantile, and overall archetypal or atavistic (including mythological) qualities of DMT further define its content, as elaborated in Michael et al. (2021). In contrast, the NDE typically features a more predictable and personally relevant nature—such as life reviews, or when culture-specific content appears, it generally aligns with that of the experiencer. Regarding other modes of comparison between the two states, such as dynamics or intensity, the DMT experience is certainly more fluctuating, prolific, and overwhelming. This is evidenced by the greater amount of DMT content discussed in the section after Table 2 and in the table in SM 3, the absence of any indication of ‘flux’ (Michael et al., 2023a), such as rapid transitions or movement, and the lack of expressions of extreme ‘intensity’ (Michael et al., 2023a) in NDEs. In comparison, the NDE at least seems to possess a more stable, somewhat circumscribed nature; while it can be intense, it is not excessively so. The sheer abundance of DMT content may also account for the imagery related to death that inevitably arises, given that the archetype of death is deeply rooted in the collective psyche of human and mortal experience.

A minority of DMT experiences exhibited at least one feature or a small cluster of NDE-typical content—such as a sense of dying, the void, or the light. However, as discussed earlier, these cannot be dissected into the dimensions of structure *and* content. Thus, DMT may not uniquely reproduce these components. No DMT report in this study contained the fuller syndrome of content that characterises most NDEs, nor did any follow a repeatable sequence, which may be observed to some extent in certain NDEs. In this regard, at most, DMT might be viewed as an *occasional* and *partial* ‘NDE-ogen’ (reproducing an NDE with high fidelity). More accurately, DMT could be conceptualised simply as an ‘*NDE-mimetic’* due to significant differences in theme prevalence and, importantly, the vast differences in content. Given the currently preliminary, indirect evidence of its release near death, along with the infinitely more multifaceted biology occurring at that time—see *DMT’s potential role in the NDE* below*—*the term NDE-mimetic is akin to historically describing DMT as a psychotomimetic after its physiological implication in psychosis was unsupported, and its phenomenology only superficially mirrored psychosis.

However, as discussed in detail below, it is important to remember that some NDEs contain more idiosyncratic elements. Therefore, DMT may reproduce these types at a nuanced content level, and for currently unexplained reasons (e.g., genetic variation), it may also have biological implications. Finally, a minority of the DMT experiences identified here were described as NDE-like in both number and possibly in the sequence of NDE features, particularly *LG* and *EM—though their* content still retained a stereotypical DMT quality.

### Idiosyncratic NDEs

Despite the overarching conclusion of this article that DMT’s distinct structural features set it apart from NDEs, it would be a mistake not to acknowledge that certain elements of some NDEs may indeed be indistinguishable from DMT (or other psychedelics). Some cases show superficial similarities, such as fractal-geometric motifs—illustrated by the example of 30-year-old *GJ,* who experienced three NDEs within 5 weeks. It is important to note that this individual has congenital time–space and numerical-visual synesthesia, raising intriguing areas for research into the distinctive content of NDEs across neurodiversities or neurological conditions. Initially, his NDE following an anaphylactic shock from prawns involved geometric visuals that ceased after the loss and recovery of consciousness. Two weeks later, after septicaemia likely caused by a horsefly bite, he experienced an NDE featuring similar entoptic patterns for 3 h; and finally, during an NDE after a lightning strike that threw him 15 feet, he again encountered beautiful and terrifying entoptics for 30 min in a blinded state (Luke, in preparation).

The phenomenon of encountering more complex geometry, such as a ‘wheel’-like structure traditionally associated with the kappa-opioid agonist and dynorphin-mimicking *Salvanorin A,* has also been reported during NDEs: “I stared at a huge dark wheel with stars and other celestial bodies, which slowly revolved,” “I could clearly see a mandala. This wheel rotated very slowly, and I felt that this wheel could not be stopped,” “I rode with the Universe of God in the wheel of life, and I became a spoke in the wheel” (Smith, 2019). *Wilson,* during a car crash, also reported an NDE in which he observed an object that:

“resembled a giant water wheel lying on its side, and rotating… it was larger than…the world… I had seen the object before, and I will see it again when I die… I was about to be subject to…‘reincarnation’. This was why the wheel had come. I… [was] a discrepancy…to be fixed” (Smith, 2019).

Judson and Wiltshaw (1983) report on a man with CNS-implicated lymphoma who becomes comatose, later recounting an NDE in which he finds himself on a “rocky… sandy… high plateau” under a sky of “pale gold.” He states that his “shape is more or less that of a cube, but is in [the] process of transformation, perhaps towards a globe… a greater bliss and discovery of being are immanent.” Eventually, he is administered 0.4 mg I.V. naloxone (an opioid antagonist), and consequently, the atmosphere shifts to a negative and seemingly delirious state as he confuses the assisting doctors with “aliens” trying to “abduct me into their world.” The “cube” in a state of “transformation” resonates with the five cases of ‘hypercubes’ in the current DMT analysis, and his identification with it is similar to the report of participant *ST* (Michael et al., 2021). Other instances of geometric constructs in NDEs include Hou et al.’s (2013) report on the NDEs of three survivors of brain injury. One instance describes a structure of light appearing as “a form-changing creature, which could smoothly flow from one 3-dimensional geometrical structure into another.” As the experiencer “moved towards (it) by flying, (it) continued to move away as it flowed from one structure into another” until it approached and finally engulfed him. This transforming geometric light structure replicates the self-transforming entities or objects (19–22%), most of which are geometric (8–22%) in the current DMT study (Michael et al., 2021).

An NDE reported by participant *ST* (Michael, in preparation) closely mirrored the sci-fi motifs of the DMT sphere, featuring a light-filled space inhabited by entities that presented him with a symbolic script, which he interpreted as taking place within a spaceship. Similarly, another NDE involved crawling through an endless maze of “tunnels” found inside a “spacecraft” (Montemayor, 2020). This is indicative of the under-researched parallels between NDEs and UFO or alien abduction phenomena (Michael, 2024), and the idea of NDEs which appear more like DMT owing to their reminiscence of such phenomena suggest DMT’s greater simulation of UFO/abduction phenomena versus NDEs (Michael et al., 2021). Furthermore, a playwright contacted by the authors reported encountering an entity with “a thousand eyes” (Luke, *personal communication,* April 26, 2021), an entity that also confronted DMT participant *RH* (*see* SM 6). This depiction of many-eyed, serpentine beings associated with death clearly represents a cross-cultural archetype (Luke, 2008).

The 1974 NDE of Victor Solow, who suffered a heart attack that left him clinically dead for 23 min (Schlieter, 2018), involved “moving at high speed towards a net of great luminosity. The strands and knots… were vibrating with a tremendous cold energy.” This may serve as a new manifestation of the threshold representative of advancing technology. He explains, “The grid appeared as a barrier that would prevent further travel,” reflecting another modern NDE in which the subject encountered a “force-field” before being compelled to return due to “not having the right information” (Witting, 2010). Solow slowed down upon reaching the construct, suggesting a lucid-dream-like control, after which:

“[it] increased to a blinding intensity, which drained, absorbed and transformed me at the same time… The grid was like a transformer, an energy converter transporting me through form and into formlessness, beyond space and time… This new ‘I’…had no connection to ego. It was final, unchangeable, indivisible, indestructible, pure spirit… part of some infinite, harmonious and ordered whole.”

A grid of light is reminiscent of the DMT experiences of several participants (e.g., *GR, SH*), resembling ‘blueprint’-like or ‘axes’ designs. This imagery also connects to the high-dose LSD experiences of visionary artist Grey (2004), whose work *Net of Being* (and the similar *Oversoul*) depicts a ‘universal mind-lattice’ composed of intricate strands of flames, resonating with the Buddhist concept of Indra’s Net. He describes it as representing ‘all individuals as nodes in a network,’ which mirrors Solow’s idea of the ‘indivisible spirit’ reconciled with being part of an ‘infinite whole’ (further reflecting *ST*’s NDE above, which included feeling ‘one with the white light yet separable from it’).

Finally, the *Bardo Thodol* (as described in The Tibetan Book of Living and Dying, Rinpoche, 1992)*—*which may have been influenced, if not by ‘Deloks,’ i.e., Tibetan mediums entering NDE-like trances, then by comparable Tibetan practices (Prude, 2024)—outlines the four phases of the luminous bardo of *Dharmata*, in which “mind and its fundamental nature gradually become … manifest … through this dimension of light and energy … mind unfolds from its purest state, the Ground Luminosity, towards its manifestation as form … in the bardo of becoming” (which echoes the wheeled NDEs above suggesting reincarnation, as well as the light grid of Solow’s NDE facilitating the interchange between form and formlessness). This illustrates that the forms presented upon death are mind-dependent, where the action of psychedelics indeed serves to ‘manifest the mind.’ Crucially, the second of the Dharmata phases, the *Union* (or *The Deities*), is the process where “the luminosity manifests in the form of buddhas or deities … the forty-two peaceful and fifty-eight wrathful deities … taking on their own characteristic mandala pattern of five-fold clusters,” which also appears synesthetic as “the brilliant light they emanate is blinding … the sound is tremendous, like the roaring of a thousand thunderclaps.” This closely resembles DMT trips in which beings, often speculated to be complex emanations of one’s own psyche (Luke and Spowers, 2018), appear to coalesce into geometric mandala-like forms (and is very reminiscent of the McKennian ‘Self-transforming machine elf’)—where the mandala itself is considered, from a Jungian perspective, to symbolise the Self upon final individuation (Jung, 1969). Finally, Sogyal writes that “From yourself and from the deities, very fine shafts of light stream out, joining your hearts with theirs. Countless luminous spheres … increase and then roll up … into you”—which echoes another elf encounter on DMT wherein the “elves sat on top of me, shoving in all this light into … my solar plexus,” as well as an independent report in which they were “pouring a golden, viscous liquid … into the middle of my abdomen” (Luke, 2013).

Regarding the NDE narratives used in this study, one patient, in particular, reports two near-death experiences that could be considered especially idiosyncratic and particularly similar to DMT. Notably, one is one of only two narratives of an overtly distressing nature, where the experiencer encounters an initially malevolent entity and is taken to a hellish realm amid a river of lost souls (similarly reported by DMT participant *OD*). The content is possibly indistinguishable from DMT, emphasising the manifestation of the entity, which displays the appearance of a face and hand, serving as an archetypal trickster with its deceptive, luring behaviour (also reported by *DD*)—but who also benignly performs ‘psychic surgery’ (reported by *RV*) and may be characterised as a ‘being of light’ (e.g., *RH*) in its shining, albeit black, appearance, which, along with its ambiguous role, crystallises its DMT-like content.

Other observable features in this idiosyncratic NDE (as well as typical NDEs overall) and several present and previous DMT reports (Michael et al., 2021, 2023a) include a *Deus Ex Machina* motif (not thematised but expressed by *RH*), telepathic communication with entities, lucid-dream-like control, a sense of hyper-reality, clairvoyant-like perception, a black void, intense fear, an attractive white light, unconditional love, and paradoxical black light. Additionally, after-effect motifs involve a sense of revitalisation, ontological shock, and transformation, including relief from addiction, enhanced spirituality, and a lack of fear of death. Fewer elements align more strongly with typical NDEs, such as an autoscopic OBE, the entity being identified as ‘God’ or ‘Holy Spirit,’ a ‘cord of light,’ the patient being halted at a threshold for fear of ‘contamination of the light,’ and ultimately being returned to complete a mission.

The aetiology of the near-death episodes in this patient was a suicide attempt, which relates to the earlier mention of ‘contamination.’ The majority of the narrative transcript is available in the Supplementary material (*see* SM 7).

### Explanatory models for similarities and differences

The conclusions of this study can be distilled into a similar phenomenological structure (with some notable discrepancies in prevalence), but a significant difference exists in the qualitative content between DMT and NDEs. The qualitative methodology of the current report, which is sensitive to revealing this level of experience, is a key reason for these findings and for the distinction from the statistical similarity between DMT and NDEs concluded by the primary quantitative comparison of the two states (Timmermann et al., 2018). However, another partial explanation may be that the laboratory context of the latter could have exaggerated this similarity, especially considering the high number of NDEs compared to those experienced in clinical environments. It should also be noted that the laboratory study included only 13 subjects, whereas this report’s 36 interviews may enhance its generalisability. Additionally, the laboratory study administered DMT (though intravenously) in doses ranging from 7 to 20 mg, with only five participants receiving the 20 mg dose, while this report utilised doses ranging from 40 to 75 mg of DMT (though inhaled), with *all* participants achieving breakthrough states indicative of the NDE.

Varela and Shear (1999), and Petitmengin (2006) provided support for the explanations below regarding a generalised correspondence between neural processes and phenomenological structure, as well as psychological processes and qualitative content inspired by ‘neurophenomenology’. Qualitative content—which may be linked to first-person, subjective, high-level, or contextually contingent experiences—has a variable nature and is therefore influenced by more top-down processes such as psychological factors. In contrast, the gross phenomenological structure—which may be associated with third-person, “objective,” low-level, or universal experiences—is more invariant and may relate to more bottom-up processes such as neural ones. Varela’s reference to pre-reflective experience is associated with invariant structure, while reflective experience (which is interpretative, conceptual, or symbolic) is connected to variable content. This suggests that personal differences cannot be entirely reduced to brain activity; instead, they must be explored through first-person methodologies like the micro-phenomenology developed by Petitmengin. This method uncovers the ‘hidden’ aspects of lived experience, capturing the personally mediated uniqueness of detailed content rooted in autobiographical conditions.

Given this, the conveniently disentangled levels of experience and their drivers should not (and cannot) be entirely disintegrated. This is clearly demonstrated by the interplay between psychedelic experiences and computational neuroscience, as unique neural shifts facilitate the amplification of psychological influence. For instance, psychedelics are believed to relax prior beliefs and models of the world (Carhart-Harris et al., 2019); however, this perspective does not fully account for the evident shaping of experiential content by such prior beliefs (Lifshitz et al., 2019), which can be vividly observed in ritual psychedelic use, where a ‘socialisation of hallucination’ may occur (Dupuis, 2021). On one hand, reduced higher-order constraints on perception may prompt the brain to search its repertoire of predictions to converge on a ‘best guess’ about its cause, thereby constructing a perceptual model. Nevertheless, this reduction may also reinforce priors at the mid- to low-levels of the neural hierarchy, whose generative models may partly consist of deeply learnt priors from specific sociocultural contexts (Safron, 2020). Finally, top-down influences on sensory experience may also be suggested by the connectivity between the transmodal association pole and sensory modules, which correlates with the imaginative faculty (Timmermann et al., 2023), as well as by preliminary data from the DMT state suggesting the circulation of neural entropy between higher-order/anterior and lower-order/posterior regions under DMT, possibly linked to cognitive interpretation applied to sensory content (Reydellet et al., 2022).

Thus, the following offers more specific examples of how neural structures and psychological content intersect in relation to the findings of this report. Neural accounts, or ‘bottom-up’ approaches, may be best suited to explain the *similarities in the phenomenological structure* identified here between DMT and NDEs. However, it is not necessary to directly invoke DMT release, as such general features are likely attainable through various other physiological or neural mechanisms that converge downstream but may be triggered independently. For instance, *Salvia divinorum* works by agonising the kappa opioid receptor, ketamine functions through antagonism of the NMDA receptor, and classical psychedelics like DMT activate the 5HT-2A receptor. Nevertheless, all have been shown to significantly disrupt the Default Mode Network (DMN; *Salvia*: Doss et al., 2020, ketamine: Bonhomme et al., 2016). This more extensive endpoint, along with other related neural activity, may contribute to the shared phenomenology of the two experiences, such as immersion in novel world models and/or the dissolution of self-models, as seen in mystical states. Similarly, despite these different initial receptor mechanisms, the claustrum is also found to be inhibited by both *Salvia* (Stiefel et al., 2014) and psilocybin (Barrett et al., 2020), where its connectivity to the DMN and task-positive networks is altered.

When considering *differences in qualitative content*, psychological ‘top-down’ models may be most suitable. The contextual discrepancies between planned DMT trips that occur in the homes of experienced participants and spontaneous NDEs faced by individuals in sudden life-threatening situations, often leading to hospital environments, certainly involve expectation effects. Such belief-driven effects are naturally heightened by the DMT experience itself, and any potential implications of endogenous psychedelic agents, or ‘endo-psychedelics,’ in NDEs would further amplify these influences from ‘set and setting’ (Hartogsohn, 2016). Specific features that appear more frequently in NDEs compared to DMT accounts, such as encounters with deceased individuals, may also relate to these processes. Additionally, other studies document NDE-like experiences that lack classical NDE indicators yet still exhibit similar phenomenological features to traditional NDEs (Charland-Verville et al., 2014), which can be linked to higher fantasy proneness in NDE-like experiences (Martial et al., 2018). Notably, only a small percentage of individuals who have had NDEs seem to be consciously aware that they are dying (Cassol et al., 2018; Van Lommel, 2002). However, it is generally accepted that the level of *content* is more sensitive to expectation effects. Similarly, many DMT participants in this study believed they were dying; however, for the most part, the content differed from that of NDEs. Interestingly, nearly all previously described ‘NDE-like’ DMT cases involved intense sensations of dying at the onset. Naturally, varying conditions of NDEs would lead to different levels of conscious recognition of being near death, such as spontaneous heart attacks or accidents versus suicides or prolonged illnesses, wherein the less expected cases, conscious suggestion effects would be less likely to manifest.

Examining the neural level, however, might also explain the significant *structural differences, particularly in prevalence* (the presence of specific features in NDEs compared to their relative absence in DMT, but perhaps also the more notable presence of other components in DMT vs. NDEs), as well as the scant similarities in content, as emphasised throughout this article. The neural model may provide greater explanatory value because the near-death state includes far more complex and nuanced conditions, in stark contrast to the straightforward influx of exogenous DMT into the brain during personal use. This is discussed in detail in Michael’s (2022c) work, especially regarding the observations about the structural features of the tunnel, the OBE encounters with the deceased, ego death, and dying sensations, which may encompass the potentially more constrained content of NDEs. However, one crucial example is Li et al. (2015), who demonstrated that, in rodent models, a wide range of neurotransmitters significantly increased upon asphyxiation, which could translate to human near-death experiences and may offer unlimited explanations for the NDE phenomenon. Serotonin was initially elevated by 20 times compared to baseline, noradrenaline by 30 times, dopamine by 12 times, and glutamate by 3 times. Interestingly, these neurotransmitter systems are upregulated by classical monoaminergic psychedelics and ketamine, which exhibited the greatest semantic similarity with NDEs (Martial et al., 2019). Although salvia (which contains salvinorin A) was also semantically linked to NDEs, the endogenous transmitter it mimics, dynorphin, was not measured by Li et al. Nevertheless, it is implicated in emotion and hallucination and is elevated during cellular stress (Nichols, 2018). Notably, an increase in serotonin was observed 4 min post-asphyxia, where serotonin may have psychedelic properties (Schmid and Bohn, 2010) and could potentially act as a primary endogenous inducer of the NDE.

Considering *content* similarities and acknowledging that top-down processes often primarily drive content, DMT could theoretically contribute biologically to the few elements that are similar even at the content level (Dying, Void, and Light). However, as noted, these features may be better examined at the structural level due to their minimal content variance. Some NDEs certainly display content resembling that of DMT, as discussed *in the* section *on* Idiosyncratic NDEs, suggesting that sufficiently psychoactive concentrations of DMT could be biologically involved in these experiences (see the next section). Nevertheless, psychological factors during the DMT experience, such as the feeling of dying, might also explain these content similarities, with the aforementioned caveat still applying.

The theoretical foundations for considering endo-psychedelics, such as DMT (or convergent mechanisms), as active during near-death experiences exist. This activity may produce entropic brain states that reconcile the paradoxically rich awareness evident in disconnected low-wakefulness, based on studies by Scott and Carhart-Harris (2019) and Gosseries and Martial (2020). Speculatively, this could represent another dimension of neural activity shared between the DMT and NDE experiences, contributing to the extensive overlapping phenomenology and potential content. Similarly, empirical evidence suggests the generation of brain states characterised by specific oscillations, particularly coherent gamma activity, in rodent models as well as in human true death (based on studies such as Vicente et al., 2022 and Xu et al., 2023). These findings may indicate a shared neural dimension that accounts for these similarities due to analogous (though not identical) increases in gamma in the DMT state. For instance, increases in gamma have been associated with functional connectivity at both the transmodal association pole of the cortex and the limbic network (Timmermann et al., 2023), as well as with mystical experiences (Pallavicini et al., 2021). Further discussion can be found in the Supplementary material (*see* SM 11) and in the discussion by Michael (2022c).

### DMT’s potential role in the NDE

While still considering the complexities related to brain states at death—particularly regarding the potential role of endogenous DMT’s implication in NDEs—it was initially assumed that DMT, which was first identified in human peripheral tissues (Barker et al., 2012) and in the pineal gland of rodents (Barker et al., 2013), would be transported to the central nervous system (CNS) during extreme stress, such as near-death situations. However, monoamine oxidases (MAOs) in peripheral tissues would significantly hinder the achievement of higher concentrations. Dean et al. (2019) identified that although the pineal organ can produce DMT, it is not essential for its elevation. Furthermore, as highlighted by Nichols (2017, 2018), evidence for a CNS uptake mechanism for DMT is extremely limited. Regardless, the concentrations of endogenous DMT are orders of magnitude lower than those needed to produce psychoactive effects. If DMT is secreted by the pineal gland, an unfeasible proportion of its mass would need to consist of sufficient DMT. The enzymatic machinery necessary for DMT synthesis (INMT-AADC co-localisation) appears not to have been studied in human brain tissue, even though it has been identified in rodent brains (Dean et al., 2019). Nevertheless, Borjigin and Chavez (2022) suggest that this metabolic machinery is generally conserved across species. Nichols and Nichols (2020) emphasises the significant increase (X250) in serotonin levels from the baseline reported (Li et al., 2015), which, considering the approximate sixfold elevation in DMT (Dean et al., 2019) and the fact that serotonin has a much higher affinity for the 5HT-2A receptor, implies that DMT likely plays a limited role, if any, in NDEs and that serotonin itself may be a more suitable candidate for inducing these experiences (if it is indeed psychedelic, Schmid and Bohn, 2010). However, Dean (*personal communication,* December 17, 2019) counters these minimisations of DMT’s role, arguing that their microdialysis sampling technique may underestimate DMT quantities. He points out that the sampling rate was only at 15-min intervals, while higher concentrations could occur before or after this timeframe, potentially as frequent as 1-min intervals, as noted in Li et al. (2015). Additionally, their study focused solely on the occipital cortex, where DMT may be more widely utilised as a compound.

Additionally, Borjigin and Chavez (2022) notes that despite potential structural or functional damage to the brain during near-death states, this may enhance the capacity of DMT to produce its psychedelic effects, notwithstanding the previously mentioned critiques. For instance, any plausible hypoxic destabilisation of the DMN that might already be occurring could facilitate this, as DMN disintegration serves as a core neural mechanism behind DMT’s effects (Timmermann, 2019). However, this destabilisation may also induce psychedelic effects independently of endo-DMT, echoing the neural hypotheses previously discussed. This aligns with the present author’s speculation (Michael, 2022c) that any synergistic interaction between brain activity near death—such as region-specific paroxysms or the aforementioned high-frequency oscillations—and released DMT may be critical to NDE phenomenology. Nonetheless, this would not apply to certain conditions, such as specific coma-like states, given the reduced sensitivity to psychedelics in DoC patients due to the necessity of specific, intact neural systems for consciousness-enhancing complexity to increase through psychedelics (Gosseries and Martial, 2020).

Finally, regardless of the specific neural processes involved near death, the neuroprotective effects of DMT might account for its release; however, the simultaneous occurrence of profound experiences may also have an evolutionary basis, offering adaptive prosocial after-effects (e.g., Shushan, 2009; Lake, 2019), as further discussed in the Supplementary material (*see* SM 12) and Michael (2022c).

Briefly, it may not be physiologically feasible for DMT to play a role in the NDE. The situation presents a much more intricate picture than merely DMT release, or indeed, DMT might not be necessary given the downstream mechanisms that converge on similar phenomenology.

### Clinical application

The question of the clinical utility of DMT is addressed more comprehensively in separate studies (Michael et al., 2021, 2023a), but the degree of comparability between this experience and NDEs may have therapeutic implications. NDEs are known to bring about numerous beneficial changes in well-being and personality, such as reduced fear of death, a greater sense of spirituality, decreased psychopathology, and increased concern for others and the world (Van Lommel, 2002; Groth-Marnat and Summers, 1998), all of which appear to be uniquely connected to the subjective experience (Greyson, 1981, 1992, 2001). Thus, as Michael (2022a) emphasises, if psychedelic experiences during therapy were more similar to NDEs, such positive changes might become even more profound. Additionally, considering the clear parallels between DMT/psychedelics and NDEs, individuals who have experienced unsupported NDEs also require adequate preparation and integration of their experiences. This paper concludes that DMT is best understood as merely an NDE-mimetic, suggesting that substances providing better simulations should be explored further.

High-dose psilocybin, for instance, may reliably induce NDE content when the active psilocin, which is merely a single hydroxyl group away from DMT, surpasses the ‘breakthrough’ thresholds typical of DMT use (Michael, 2022b). Since any drug with high simulational fidelity to NDEs is characterised by themes of death and dying, and the primary effect of NDEs is the reduction of death anxiety (Bianco et al., 2024; Bianco, 2017), such a treatment may be most appropriately applied in terminally ill patient populations—where psilocybin has already been demonstrated to produce significant and sustained reductions in existential anxiety (Ross et al., 2016; Agin-Liebes et al., 2020). However, the potential presence of existential concern, the lack of ritual, and the over-medicalisation of the concept and treatment of death and suffering are important caveats to consider in the context of psychedelic end-of-life care (Varley, 2019; Dutta, 2012). This particularly NDE-like psychedelic should not be restricted to these populations, as some evidence suggests that different psychopathologies might share a common underlying factor of death anxiety, explaining the impressive transdiagnostic effectiveness of psychedelics (Moreton et al., 2020). 5-MeO-DMT also closely replicates the mystical features of NDEs (though these arguably lack specific content; Michael et al., 2023b), which participants have used to help integrate their own NDE—and changa, when combined with significant MAOI doses, might be even more NDE-like than *N,N-DMT* (Michael et al., 2024). Indeed, creating a mixture with the same combination of neurotransmitters (or mimicking agonists) that increase during rodent death, potentially alongside neural stimulation or entrainment that mirrors local discharges and gamma surges, contributing differently to various experiential themes, would be a fascinating endeavour to replicate the exact content of the NDE for both modelling and therapeutic purposes.

## Limitations

In terms of limitations related to the present study’s comparative analyses, particularly between NDEs and *NN-*DMT experiences, one example is the restriction of near-death experiencer narratives to French-speaking countries, primarily Belgium and France. This leads to a lack of content from cross-cultural sources, as DMT participants are predominantly British. Regarding the ‘idiosyncratic’ NDEs mentioned earlier and their potential to diminish the significant content differences highlighted between DMT experiences and NDEs, using NDEs from diverse cultures could have reduced these differences due to their more distinct characteristics compared to Western NDEs. For example, NDEs from a Chinese sample, most of whom were Buddhist, exhibited many qualitative traits reminiscent of psychedelic experiences, such as encountering a transforming geometric creature of light (Hou et al., 2013). An extreme case could involve NDEs from indigenous societies, which are typically shamanic and often utilise entheogenic substances, reflecting their culturally embedded psychedelic journeys (Shushan, 2018). Thus, since the DMT experiences in the present study are almost exclusively drawn from a European sample, it importantly facilitated a clearer comparison with the French NDE narratives, focusing the analysis on *DMT* versus *NDE* rather than complicating the study with more pronounced sociocultural differences. However, future studies should include a more socioculturally diverse population to explore how culture influences experiential content, which in turn affects the comparability between NDEs and psychedelics.

Similarly, the NDE narratives used were selectively derived from classical near-death experiences characterised by proximity to death under anoxic conditions. This contrasts with classical NDEs that arise from different aetiologies, which may present unique content and display varying similarities and differences compared to the DMT state. Furthermore, when compared to NDEs resulting from other physiological states without a mortal threat, anticipatory near-death (‘fear-death’) situations, or even NDE-like cases (e.g., syncope, epilepsy, hypnosis, and more), different findings may also surface. However, future research should explore this possibility. Moreover, certain NDE-like conditions may be better simulated by DMT, considering that DMT is one of several inducers of an NDE-like experience that occurs without the complex physiological cascade associated with actual death. Focusing deeply on one of the many possible models—in this case, DMT—as well as on classical, anoxic NDEs was a necessary pragmatic decision that facilitated a comparative study with fewer changing variables.

Additionally, the retrospective near-death narratives contained significantly less written content than the prospective DMT structured interviews. If richer NDE interview transcripts were compared, such as those in the study by Michael et al. (2023b, 2024), the more nuanced content could have influenced the comparison in unpredictable ways. This connects to another potential limitation, as the current study did not conduct a thematic analysis of the NDE but instead utilised a partially modified and extended version that already existed (Cassol et al., 2018), including its raw qualitative data. This may have resulted in discrepancies in the styles of thematisation, including a lack of direct relationships between themes; however, corresponding themes between the DMT and NDE analyses remained identifiable (see Table 2).

In addition, only one author (PM) formally conducted the thematic analysis of the DMT transcripts both here and in prior publications (Michael et al., 2021, 2023a). However, this analysis was thoroughly supervised by DL and OR and was found to be robustly faithful to the representations in the interview data. Importantly, the exceptionally high level of similarity in specific content amongst DMT participants—not only in the experiences of Michael et al. (2021, 2023a) but also across numerous other studies of DMT phenomenology, as discussed extensively in both reports—provides strong support for PM’s DMT analyses to reliably reflect the underlying content. Furthermore, it can be argued that since the analyses concentrated on content rather than employing techniques that emphasise interpretation, which can implicate the subjectivity of the researcher, there is less potential for lower inter-rater reliability.

## Data Availability

The raw data supporting the conclusions of this article will be made available by the authors without undue reservation.
