# Supplementary material for: An encounter with death: a comparative thematic and content analysis of naturalistic DMT experiences and the near-death experience
Source: Front Psychol. 2025 Mar 5;16:1532937. doi: 10.3389/fpsyg.2025.1532937 (PMC11920758; doi:10.3389/fpsyg.2025.1532937)
Supplement: Supplementary file 1 [file Supplementary_file_1.docx]

**Supplementary Materials**

**SM 1.**

## **Thematic Analysis of the DMT Experience – The Mystical Experience**

All participants articulated at least one theme subsumed by the category ‘The Mystical Experience’. These were not deductively predicated on the 7 dimensions of the classical mystical experience (as schematised by Barrett et al, 2015), but through inductive analyses the final themes did align with these and the names were kept consistent. The two themes of ‘paradoxicality’ and ‘entheogenesis’, however, are not dimensions of said schema of the experience.

*Ego Dissolution*

Eleven interviews of the 36 reflected participants’ experiences of ‘ego dissolution’. Across responses, they described a disintegration of their sense of self, going from a sense of individual persona to that of a perseveration of conscious awareness yet lacking subjective identity. *GR* expressed:

*“I was just going, my sense of self dissipated, I just did not exist anymore… It was like everything literally was just…an infinite universe of raw information”*

When asked if he encountered a presence with him, he responded *“But I mean myself wasn’t there in the first place, there was no me or myself” -* implying the necessity of the integrity of one’s own sense of self for the mediation of the sense of another’s.

Although the participants described their experience of ego dissolution within a narrative of “death,” such as *AN’s* description of “*it was like I’d died,”* this death of ego wasn’t associated with annihilation into unconscious oblivion but rather the ablation of the egoic structure with continued expanded awareness.

There appeared to be a discrepancy in responses as to whether ego dissolution was enjoyable. Whereas in *RH’s* case he elicits the fearfulness of such experience, “*Powerlessness, terrible powerlessness… The dying bit is the worst bit of not knowing,*” *JB* revelled in his selflessness shedding “*tears of beaut[y]”* and resolved his experience, “*still saying ‘I’ love everything”.*

*Unity*

Themes of unitivity transpired across nine interviews, where participants painted imagery of the boundaries to the individual self dissipating and a “*merging*” of self into their surroundings, or indeed, all that is. As recounted by *RS*:

*“The beings, the geometry, they’re all just interrelated, even myself as I experience it – I move my body and it moves as well. The boundaries of self, the bounds of self and other is very thin”*

In some responses, such descriptions of fusion were applied with respect to the entities encountered during the DMT experience. For example, “*It felt like [the entity] had his own autonomy but he also felt like a part of me, because at that point I felt like I was part of everything*” (*RS*), and *“I didn’t feel like there was a separate entity, other than the mass entity. I felt more like I was everything rather than that separation*” (*AN*).

For the most part, the experiences of unity reflected positive feelings, evoking a felt sense of “*belonging*”, “universal *connection”,* and *“love.”*

*Transcendence of Time & Space*

A perception of the escape from the structures of both space and time appeared as another fundamental theme across eight participants’ experiences. In some instances the passage of time was void entirely, leading to an impossibility of its quantification:

*“It was timeless within there… I didn’t really have any concept of self…let alone the concept of timeframe... And now I’m looking back it's easy to say, well it just felt like a few minutes” (FF)*

In other instances the flow of time’s arrow no longer seemed to move in a linear manner, where *DD* articulates an especially ineffable state of the novel complexification of his temporal experience - which was intimately tied to the appearance of a hyperdimensional cube.

*“...it felt like there were all sorts of possibilities that were coming in, converging… It was like all levels of this linear time we’re on now- …everything that made sense just totally skewed and all mixed together and time and everything all converged, like has this been 10 years or has it been 2 seconds!?*

*Multifaceted, all possibilities of, like after this experiment, before this experiment, during the experiment, 50 years down the line, but like all happening at the same time…instantaneously… those levels of perception that just completely fracture away and twist and turn…*

*And all the lines of time where converging in a heartbeat though, in a heartbeat, and you let that go… like I was spiralling into this cuboid that was drawing me…and it kind of splintered, and I don’t know if it was 2D or 3D…  I wasn’t thinking it at the time- but I'm thinking, yes it could have been different timelines”*

The experience of space during DMT also became ineffable and radically removed from ordinary perception, where *RH* below mirrors *DD* above in the paradoxical explosion not of time but of space.

*“There was something about there not being a direction. At first it alarmed me, because I was thinking ‘Concentrate deeper’, except there wasn’t any deeper, that was the thing. It was like everything was going back that way, down up. And not just that, every single part of it was fractalizing, there was nothing to hold onto, nothing I could grip, or do anything. Everything was just expanding from every point, no end to it”*

One participant, *SP,* alluded to the void-like nature of the space, when he responded after being asked if he found himself somewhere else, “*more like nowhere”.*

Finally*,* in referring to the idea that one *“understand[s] and feel[s] the experience of multidirectional time in a trip”, RS evokes the peculiar and profound concept that, though “it doesn’t make sense…it’s also like you never left as well. Because it’s like I came here, and you never really left, you’re still kind of there”*

*Noetic Experience*

Eight participants also reported states of insight during their DMT experiences encompassing a certain noetic quality. Many of the reports described the reality experienced as being “*more real*” or “*realer*” than everyday waking consciousness, suggesting a perceived capacity to connect with the base of being, or as *ML* poignantly frames it, “*the bigger picture. The whole purpose of it all. The whole puzzle of life”.*

This distinction between a dreamlike, everyday reality and the true nature of reality on DMT was, again, vividly encapsulated in *LR’s* report:

“*...like this is my essence and this is the true form of all things and everything that I am when I’m not paying attention to the true form of all things is kind of like, its like that’s the dream, that’s the distraction from the reality, and this is the reality that I’m in”*

For some, this noesis was associated with revelations of a specific metaphysical nature, such as *MP* disclosing the feeling of *“Knowing you’re immortal, but there’s no death, knowing there’s much more to reality than just the physical”.*

Interestingly, although the DMT offered a sense of connecting to greater fundamental truths, two of the participants questioned the value of entering this *“knowing”* against the backdrop of the normality, yet specialness, of quotidian life. Using the 1999 film “The Matrix” to tease apart this important tension, and to gesture toward a cautioning against the direct encounter with the ‘mysteries’, *RV,* finally, very evocatively described:

*“I almost feel not entirely right about taking people out of the Matrix unless they’re absolutely called to it, you know what I mean? Like should I give my wife the opportunity to see this? I don’t know, not unless she’s absolutely called to it, because it’s* so *disorientating. Perhaps it’s better just to live in the Matrix, and try to* find love *and* be love*,* find humility *and* be humility, *and* try and live, try and be everything you can*, without this extraordinary shamanic experience, which is so real you know…*

*…every attempt at being spiritual or religious…is some attempt to connect with this. However misguided the religion- there’s an attempt to try and wake us up to this extraordinary thing, and truth… we talk about spirituality, this kind of nebulous mystery, this unknowable thing that- and for most people it remains that, this kind of hunch you feel inside yourself, there’s an intuition of something beyond, but to just be (smacking noise) immersed in the absolute Is-ness of it…!”*

*Deeply Positive Mood*

Many participants, eleven precisely, expressly articulated having deeply positive experiences whilst on DMT.

With descriptions of feeling “*completely cocooned*”, “*womb*”-like (*AN*) an “*absence of fear*”, and a “pure loving experience” (*SP)* during their journeys, the DMT seemingly promoted a deep sense of safety in participants.

In the case of *FF*, this state of profound serenity allowed him to feel intense gratitude for the honor of being able to partake in a realm well beyond the normal state of consciousness.

*“…the second I calm down…I’m like ‘Oh it’s here’, and you feel safe to observe and it’s blissful, I’m blissed. I feel blessed that I’ve had this opportunity to go there again… I was just spectating this phenomenally alien environment I felt privileged to be seeing, I felt blissed by it all”*

Catharsis, that is, a process of freeing from oppressive emotional experience, also appeared to positively characterise many subjects’ reports, with *AF* affirming that *“it was very liberating”*, *SH* similarly stating *“such a release from everything…I couldn't stop laughing…for a really long time*” in the midst of an inexpressibly beautiful world, reiterating “*I feel amazing now…on top of the world! Like I’m made of this gold light, that's how it feels”*, and *JR* offering that after “*processing the energies that are difficult to release… it feels like you’re in alignment with everything”*

Saliently, all of *AN, SP* and *AF’s* experiences of positive mood and catharsis were dovetailed with sensations of pure being (see Ego dissolution, above), such as “this feeling of being aware of my own consciousness”, or statements that “I had that sensation of just existing”.

*Sense of Sacredness*

Two participants offered the idea of sacredness – a feeling of sacrosanctity which touched them deeply. *JB* formulated it as a “pretty mystical- pretty religious experience, I think you can describe it as. Melting into the universe… A Oneness, a wholeness, a universal connection”, while *AV* alludes to the sensorial beauty and quiescence of her safe containment:

*“I tried to find a place where I could just be in this place in silence… The sound of the water was coming down, the crisp sound of burning wood, was trying to keep me in this sacred place. And I was thinking, all this sacredness…in the middle of a ceremony… I was telling myself, ‘you are in a sacred space, just be’”*

*Ineffability*

Another theme most endorsed by the experiencers, in eleven instances, was the conviction of the inconceivability to parse the exact nature of their experience into language. *GR* vividly informs us that he could barely conceptualise it as the experience unfolded, asserting that “*I wasn’t aware of what I was seeing, it was something beyond my capacity to imagine, process, intellectualise, understand or anything like that.* So *beyond anything of that sort”*

*ST* articulates the following, in valiant attempts to formulate into words his experience with an object, seemingly not of the dimensionality one is typically used to, and its confounding manoeuvres:

*“[Because] you’re looking at patterns, your brain predicts what this thing’s supposed to do next, because of what it looks like it’s* been *doing. But this is showing you ‘Look mate, you cannot predict because this one has multidimensional directions it can go to that you can’t even fathom’. Like ‘Oh that's possible!?’… You think it’s just up and down, and it goes inside out and sideways at the same time, and you’re like ‘How the fuck, what!!?... did one line just-“ I can’t describe it…*

*…there seems to be a dance kind of thing. It was synchronous as well as chaotic if that makes any sense. The chaos seems to be organised, does that make sense? If you’re looking at what makes something up and it’s chaos, but on a large scale its beautiful and almost synchronous- that's the only word to describe that sort of thing… It’s just too many words”*

*Paradoxicality*

Not unlike the above, six participants gave a sense of parts of their DMT journeys being entirely impossible, yet cannot deny their experience of it – often capturing a reconciliation of what would normally be necessarily diametrically opposite. Especially akin to *ST’*s above description, *JM* also describes logical incongruence to the visuality of what he witnessed:

“A combination of 2 and 3-D… At all kind of angles, indiscriminate placement, everything made sense wherever it was, even though it wouldn’t make sense in a normal situation. It didn’t matter if things were close, far or up, down, it all made sense either way”

*RH’s* personal paradox, however, pivoted around his encounter with an entity with which he had an ambivalence regarding his separateness – or not – to it:

*“This is what I’m trying to get my head around…we were kind of* one *thing, yet I realise there was a kind of separation, because I could reach to it…*

*Interviewer: So later on when you suddenly realised you're yourself, but it’s a different self to the ego you have now, as you associated yourself with this other entity?*

*Tough one… I think I was pretty much me again… it kind of makes sense and it doesn’t make sense. Perhaps it isn’t quite me actually, I don’t know”*

*Entheogenesis*

Finally, in three interviews the concept of the experience leading to a personal identification with God – a feeling not simply being at one, but the very same as, this ultimate principle – was communicated. ‘Entheogen’, denoting the generation of the divine within, *SP*’s second experience involved “*a God-like feeling to it, in a sense,… I guess there wasn’t anything else, as the point was there not being* anything else *apart from me”.* In his first journey, he elaborates on a sensation of participating in the creative function of divinity, as well as the challenge of avoiding the ego inflation that one may be in danger of after such a transfiguration, which was echoed by *JB* by referring to “*a ‘Jesus’ sort of feel, an enlightened ego thing. You feel like, Wow, I’m so special… Then this kind of laughing realisation…and how absurd it sounds to yourself*:

*“It just felt like being, like, God, [or]* a *God… I was able to, sort of, to encompass that feeling of being everything…*

*It just felt so amazing, like Oh My God! Its like I’ve just like, like I’ve just farted out the universe or something…*

*…when I was taking LSD, [my partner and I] referred to it as the messiah complex, I assumed that’s what everyone gets… When you start realising this idea that we’re all potentially Gods and we all create our own…reality…*

*It reminded me of being slightly chosen in the sense that… it was a lovely experience of being a true king or leader or whatever, the most powerful ones who would be- Anyone can be that person… that feeling of being very special, but…without ego…*

*It just felt like there was some part of being intrinsically involved with the, sort of, flowing or development of consciousness…*

*So time distortions is like witnessing the creation of the universe, for me I guess I feel special that I was able to witness that… I guess it was like being born. Being born and quickly gaining consciousness, that’s what it felt like”*

**SM 2.**

## **NDE Scale applied to DMT Field study, Laboratory study participants and Classical NDEs**


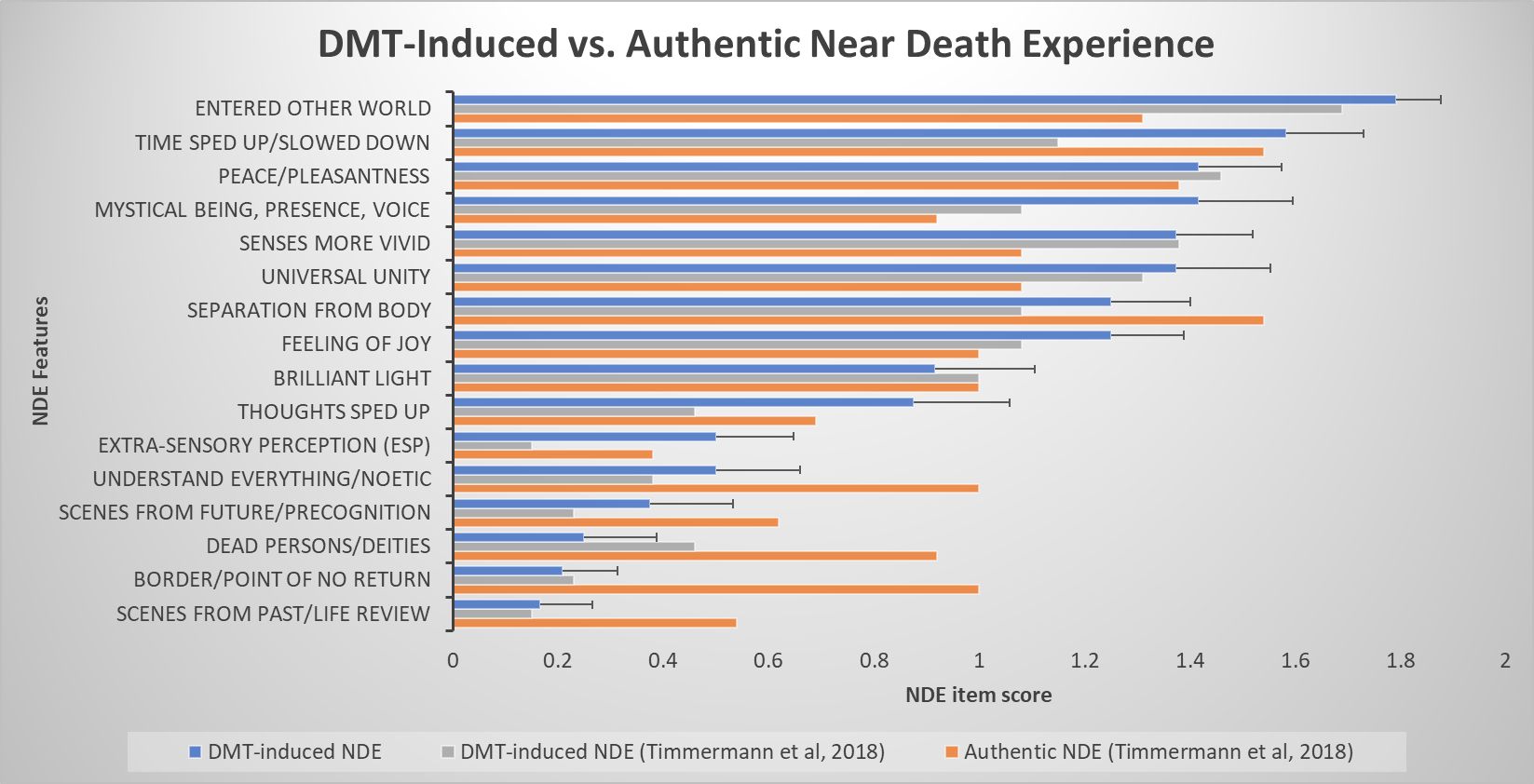


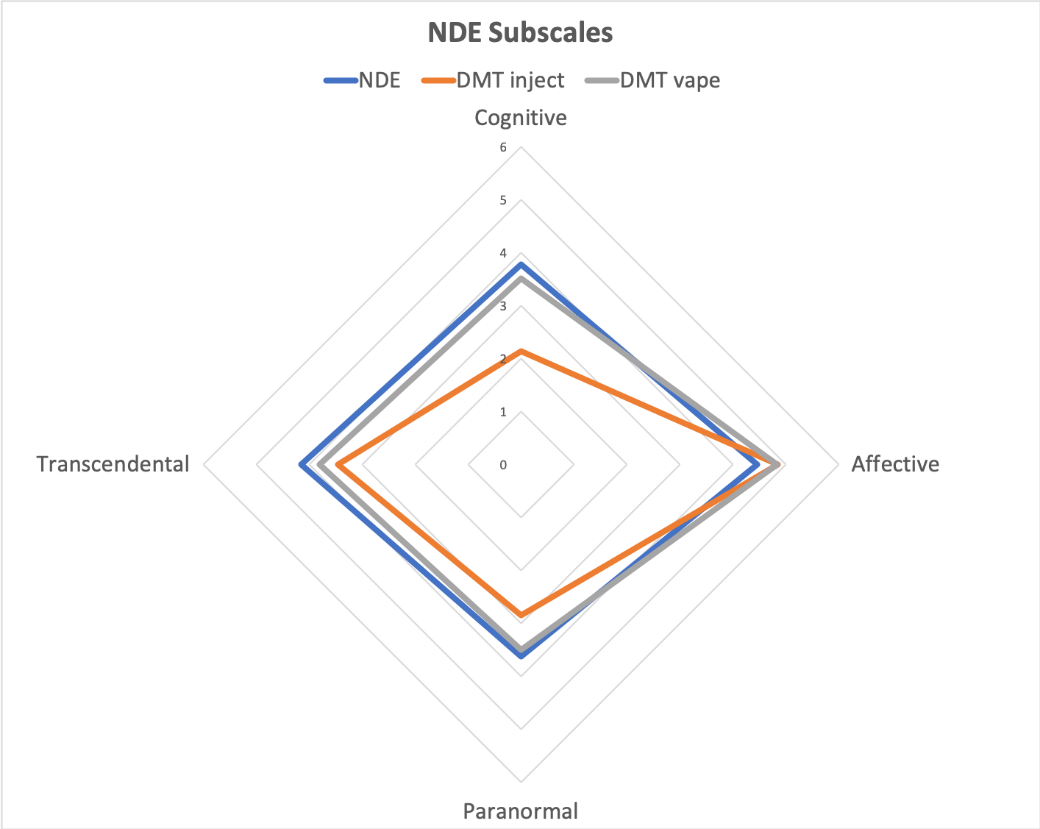


The 1st figure is adapted from ‘DMT models the Near-death Experience’, a laboratory DMT study comparing with authentic NDEs (Timmermann et al, 2018), where the results from the NDE Scale as applied to participants on the DMT field study of the present paper is superimposed. The lab. study found no significant differences between the two experiences on all scale items. However, a very conservative mode of correcting for multiple analyses, Bonferroni, was employed – and although statistical analyses could not be performed due to lacking the raw data from Timmermann et al (2018), the incorporation of the field study data does reveal some apparent discrepancies, at least on a numerical level, between both the two DMT studies and between the field study and the NDEs. Reasons for this may be numerous. For instance, the number of participants (13 lab. study Vs. 29 field study), use of significantly higher dose (7+14+20mg in the dose-finding lab. study Vs. 55mg vapourised average, equivalent to approx. 20mg I.V), mode of administration, affecting pharmacokinetic action (I.V. Vs. vapourised), and the setting in itself, in turn influencing set (participants’ homes Vs. laboratory environment).

Regarding differences between the field and lab. studies of DMT (again only numerically, but where substantial numerical difference may imply significant differences), The *ESP* item was rated more highly in the field study Vs. the lab., which may be owing to suggestion by being part of a study which as part of its measures included a parapsychological task, or may indeed be a genuine reflection of ESP increase, due to many participants correctly scoring on said task. Comparatively, meeting *dead persons* was much more likely in the lab. study Vs. the field, which may be related to being in a clinical-type setting. *Mystical being* was also markedly higher in field study, which may be partially contributed to by the highly experienced nature of the subjects with DMT, their belonging to a psychedelic subculture with niche interests in such phenomena as entities, or familiarity with the PI’s (*DL*) work on this topic. *Thoughts sped up*, and *time distortion* were also notably higher in the field study.

In relation to differences between the present paper’s field study and the authentic near-death experiences – in general, the field study data shows scores on the NDE scale (constructed to specifically measure the NDE phenomenon) to be higher Vs. the lab. on most items, also possessing a higher overall score (15.6 vs 13.3/32). This curiously may render the *DMT* experience to be more ‘NDE-like’ than even the NDE. This is very clearly mirrored in the 2nd figure, which shows scores from each subscale of the NDES, where the field study DMT results map much more closely to the NDEs (especially on the cognitive and paranormal domains). In terms of comparing specific items, entering *other worlds* and especially encountering *mystical beings* are notably higher in the field study versus the genuine NDEs (amidst the highest 4 items), as are *vivid senses* and *thoughts sped up*. This is seen to be mirrored in the prevalence comparison of the present analysis (see main paper, Table), where the breakthrough DMT experience is characteristically replete with entities in the context of a rich immersion in distinct otherly spaces. As such, breakthrough DMT trips virtually guarantees such experiences with these features, whereas the most common NDEs are in fact shallow (Charland-Verville et al., 2014) expressing the more superficial elements (Ring, 1980). In this way, a better conceptualization is that breakthrough DMT models ‘breakthrough’ (that is, deeper) NDEs. All this said, the higher scoring on these NDE scale items from the DMT study may create a greater discrepancy with the scoring by the genuine NDE subjects themselves, thus ultimately meaning DMT (on these items) are *more* dissimilar to NDEs.

Despite Timmermann et al (2018) finding the item scores to be the same between experience-types (though using the conservative Bonferroni) – the bottom cluster of features on the 1st figure, that is, the *life review*, *border of no return*, and especially *deceased/deities* (as well as precognition, noetic experience, and ESP) appear to be more likely in NDEs versus DMT (though only via numerical inspection), which is exaggerated when comparing only with the field study. This is salient, as these three exact elements may be, more than the others, those considered to be quintessential to the concept of dying or almost dying (i.e. a perception of personal proximity to death). As per the particularly low DMT field study rating for the *deceased* item, this may represent one reliable difference between the two states, where DMT could be viewed as less NDE-like on this factor. Bolstering this idea is the most reliable study on the frequency of features across NDEs (Charland-Verville et al., 2014), identifying that, among *peace, body separation, light,* and *time distortion*, meeting the *deceased* was one of the most common NDE features. This, again, is supported by the present results of the prevalence comparison (see main paper, Table).

13 of the total 16 items on the NDES have a max. ‘ranking’ difference of 2. That is, when ranking the items on highest-lowest scores, the vast majority of them were only two or fewer ranks different between the field and lab. study. Constituting the only two studies of primary data collection of DMT experiences (field and lab.), this demonstrates remarkably similar (psychometric) results as to the NDE-like nature of the DMT trip. Importantly, despite the field study by nature not employing a placebo control, while Timermann et al (2018) did employ one while also finding no differences on all items, this evident similarity further validates the field study results as not influenced by expectation effects.

The results shown in these two above figures generally suggest a corroboration of the psychedelic literature, where emphasis is placed on psychedelics’ capacity to evoke *mystical phenomena,* as well as *transportation to other place* and the *sensed presence* effect common to high-dose psychedelics and in particular DMT. If surveying the results above, from low-high ratings on the field study, and thus generally from a greater discrepancy with NDEs to a lesser one, the nature of the items become less NDE-typical and more mystical-typical. With this in mind, such mystical-type features may be acting to ‘drive’ much of the apparent comparability between DMT and NDEs – where, in order of ranking, all of *time distortion, peace/pleasantness*, (*vivid senses*, albeit not mystical), *universal unity & body separation* (again not strictly mystical, though related to detachment from the self), and *joy*; are amongst the highest rated.

This is versus the possibly more inherently ‘NDE-like’ features being amongst the lowest rated, including a *review of life, meeting the deceased* and the *border of no return.* Such lowest-scored items relative to NDEs vindicates Greyson's (2014) factor analysis of the Mysticism Scale and the NDE Scale, which yielded a specific “NDE” factor onto which precisely these three items preferentially loaded, i.e. which reliably differentiated mystical features from NDE ones.

Finally, scoring on the item *separation from body* may not be a reliable reflection of the prevalence of this feature differ, since the NDES does not adequately distinguish between the ‘OBE’ (which phenomenology in the literature suggests more classically occurs with NDEs) and 'body dissolution’/’unawareness of the body’ (which literature, as well as the present DMT analysis, indicates is more typical of DMT). Comparably, the item of *light* on the NDES is equally as common between states, despite the “light” being ubiquitously associated with the near-death phenomenon (both popularly and in the literature), and more importantly emerging from a dark tunnel in NDEs only. These are only 2 brief examples of insufficiencies of employing quantitative questionnaires as measures, whereas when incorporating qualitative data, the experiences which are otherwise oversimplified with a discreet number of predefined categories are opened up to reveal the nuances of the content and its differential manifestation between, in this case, two altered states of consciousness. As such, the main paper of the present analysis is entirely qualitative in methodology, to embrace this novel level of analysis, and thus enabling a deeper opportunity for comparison.

**SM 3.**

## **Table 3. Features from DMT Thematic Analysis not present in comparative analysis**

*The orange highlights signify the lack of coding and all the remaining themes represent those authentically absent from the NDE*

| **An Encounter with the Other** | No. Interviews /36 (%) |  |  |
| --- | --- | --- | --- |
| ***Encountering Other Beings*** |  |  |  |
| *Transformation of persons present* | *7 (19)* |  |  |
| *DMT personification* | *5 (14)* |  |  |
| **Role & Function** |  |  |  |
| *Helping or nurturing* | *19 (53)* |  |  |
| The Guide | 8 (14) |  |  |
| The Soother | 5 (14) |  |  |
| The Playmate | 5 (14) |  |  |
| The Guardian | 5 (14) |  |  |
| The Healer | 4 (11) |  |  |
| The Muse | 1 (3) |  |  |
| *Showing or communing* | *17 (47)* |  |  |
| The Presenter | 10 (28) |  |  |
| The Teacher | 9 (25) |  |  |
| The Focuser | 5 (14) |  |  |
| *Manipulating or controlling* | *6 (17)* |  |  |
| The Experimenter | 2 (6) |  |  |
| The Orchestrator | 2 (6) |  |  |
| The Consumer | 1 (3) |  |  |
| **Appearance & Features** |  |  |  |
| Other animals | 4 (11) |  |  |
| *Otherly Creatures – Non-human/Non-animal* | *26 (72)* |  |  |
| Humanoid | 9 (25) |  |  |
| Clown-like / Jester | 4 (11) |  |  |
| Octopoid | 4 (11) |  |  |
| Insectoid | 4 (11) |  |  |
| ‘The Grey- or Mantis’-like | 2 (6) |  |  |
| Therianthropic | 2 (6) |  |  |
| Baby | 2 (6) |  |  |
| 'Me' or Autoscopy | 2 (6) |  |  |
| ‘Navi’-esque | 1 (3) |  |  |
| Space invader-like | 1 (3) |  |  |
| Synapse-like | 1 (3) |  |  |
| Faerie-like | 1 (3) |  |  |
| Indigenous spirit | 1 (3) |  |  |
| Disembodied eyes | 1 (3) |  |  |
| Stick creature | 1 (3) |  |  |
| Bottle-like | 1 (3) |  |  |
| *Sentient structures* | *9 (25)* |  |  |
| Sentient geometry | 3 (8) |  |  |
| Building / Structure (Sentient) | 3 (8) |  |  |
| Computer symbols/presence | 2 (6) |  |  |
| Aztec patterns | 1 (3) |  |  |
| Boxes | 1 (3) |  |  |
| Candy cane / Pipes | 1 (3) |  |  |
| *Specific features* | *9 (25)* |  |  |
| Voice *only* | 1 (3) |  |  |
| Mobius strip | 1 (3) |  |  |
| Multi-cultural | 1 (3) |  |  |
| Planet-sized | 1 (3) |  |  |
| Winged | 1 (3) |  |  |
| Chained | 1 (3) |  |  |
| Pods | 1 (3) |  |  |
| *Visual quality* | *19 (53)* |  |  |
| Self-transforming | 8 (22) |  |  |
| Geometric | 8 (22) |  |  |
| Cartoon-like / 2D | 6 (17) |  |  |
| Colourful | 5 (14) |  |  |
| Mechanical | 3 (8) |  |  |
| Hyperdimensional | 3 (8) |  |  |
| High-Definition | 1 (3) |  |  |
| Holographic | 1 (3) |  |  |
| Metallic | 1 (3) |  |  |
| Organic-mechanic | 1 (3) |  |  |
| **Demeanour & Nature** |  |  |  |
| *Charming and Inviting* | *20 (56)* |  |  |
| Benign / Friendly | 9 (25) |  |  |
| Jovial / Happy | 4 (11) |  |  |
| Motherly | 3 (8) |  |  |
| Graceful | 1 (3) |  |  |
| Sensual | 1 (3) |  |  |
| *Other dispositions* | *12 (33)* |  |  |
| Curious | 4 (11) |  |  |
| Childish | 3 (8) |  |  |
| Urging | 2 (6) |  |  |
| Panicking | 1 (3) |  |  |
| Secretive | 1 (3) |  |  |
| Confused | 1 (3) |  |  |
| Stubborn | 1 (3) |  |  |
| Aware of those present | 1 (3) |  |  |
| Unaware of those present | 1 (3) |  |  |
| *Nature* | *17 (47)* |  |  |
| One with or of the Beings | 10 (28) |  |  |
| Familiar | 10 (28) |  |  |
| Beautiful or Extraordinary | 4 (11) |  |  |
| Powerful | 2 (6) |  |  |
| *Expecting subject* | *6 (17)* |  |  |
| Did not expect | 3 (8) |  |  |
| Expected | 3 (8) |  |  |
| *Gender* | *17 (47)* |  |  |
| Feminine | 14 (39) |  |  |
| Masculine | 7 (19) |  |  |
| None | 1 (3) |  |  |
| **Communication & Messages** |  |  |  |
| *Communication mode* | *14 (39)* |  |  |
| Dance & Gesticulation | 3 (8) |  |  |
| Visual communication | 2 (6) |  |  |
| Contactability post-trip | 1 (3) |  |  |
| Potentially communicative | 1 (3) |  |  |
| *Messages received* | 13 (36) |  |  |
| 'The Cosmic Game’ or ‘Cosmic Giggle’ | 5 (14) |  |  |
| Love for Others and Self | 5 (14) |  |  |
| Letting Go | 3 (8) |  |  |
| Warning | 2 (6) |  |  |
| Insight into the World | 1 (3) |  |  |
|  |  |  |  |
| ***Exploring Other Worlds*** |  |  |  |
| **Scene** |  |  |  |
| *Human worlds* | *6 (17)* |  |  |
| Street | 3 (8) |  |  |
| Re-enacted surroundings | *2 (6)* |  |  |
| Building / Structure (Non-sentient) | 1 (3) |  |  |
| *Natural worlds* | *10 (28)* |  |  |
| Outer-space | 6 (17) |  |  |
| Vulva | 1 (3) |  |  |
| *Artificial worlds* | *6 (17)* |  |  |
| Mechanism | 2 (6) |  |  |
| Icicle / Waterfall | 1 (3) |  |  |
| *Children’s worlds* | *3 (8)* |  |  |
| Playpen / Nursery | 2 (6) |  |  |
| Circus-like / Children's book | 1 (3) |  |  |
| *Nebulous worlds* | *13 (36)* |  |  |
| Lattice / Gridwork | 9 (25) |  |  |
| 'Tron-like' or 'Blueprint of Universe' | 2 (6) |  |  |
| Light space | 1 (3) |  |  |
| Ether / ‘The fabric’ | 1 (3) |  |  |
| **Contents** |  |  |  |
| *Organic objects* | *16 (44)* |  |  |
| Cellular or Subcellular | 4 (11) |  |  |
| 'Intra-Body Experience' | 1 (3) |  |  |
| The Elements | 1 (3) |  |  |
| *Technological objects* | *8 (22)* |  |  |
| Mechanics & Devices | 4 (11) |  |  |
| Satellites & Spacecraft | 2 (8) |  |  |
| Medicine | 2 (8) |  |  |
| Car | 1 (3) |  |  |
| *Infantile objects* | *3 (8)* |  |  |
| *Geometric objects* | *16 (44)* |  |  |
| Spherical shapes | 7 (19) |  |  |
| Hyperdimensional structures | 6 (17) |  |  |
| Other polyhedrons | 3 (8) |  |  |
| Islamic-like sacred geometry | 2 (6) |  |  |
| Flower of life | 1 (3) |  |  |
| Mandala | 1 (3) |  |  |
| Maze | 1 (3) |  |  |
| *Symbolic objects* | *6 (17)* |  |  |
| Symbols / Signs | 5 (14) |  |  |
| Ancient language / Hieroglyphs | 3 (8) |  |  |
| Matrix code | 2 (6) |  |  |
| *Miscellaneous objects* | *14 (39)* |  |  |
| Serpentine or Cyclic | 7 (19) |  |  |
| Small barrel / tube | 2 (6) |  |  |
| Sweets | 2 (6) |  |  |
| Ladder | 1 (3) |  |  |
| ‘Magic mirror’ | 1 (3) |  |  |
| Bible-like book | 1 (3) |  |  |
| Chalice | 1 (3) |  |  |
| Antique bathtub | 1 (3) |  |  |
| ‘Spikey, jangley’ thing | 1 (3) |  |  |
| **Quality** |  |  |  |
| *Transforming or Exploding* | *7 (19)* |  |  |
| *Synthetic textures* | *15 (42)* |  |  |
| Cartoon-like or Animation | 5 (14) |  |  |
| Organic-Mechanic | 5 (14) |  |  |
| High-Definition | 4 (11) |  |  |
| Holographic or Digital | 3 (8) |  |  |
| Retro-game or Old | 2 (6) |  |  |
| *Generic textures* | *11 (31)* |  |  |
| Very colourful | 5 (14) |  |  |
| Fluid / Organic | 2 (6) |  |  |
| Painted | 2 (6) |  |  |
| Blurry | 2 (6) |  |  |
| 'Razzmatazz' / Garish | 1 (3) |  |  |
| Wavey | 1 (3) |  |  |
| Ornate | 1 (3) |  |  |
| Jewelled | 1 (3) |  |  |
| Dream-like | 1 (3) |  |  |
| ‘Organised Chaos’ | 1 (3) |  |  |
| **Encounter with the Self** |  |  |  |
|  | | | No. Interviews /36 (%) |
| ***Onset*** | | |  |
| *Sensory* | | | *14 (39)* |
| Submergence | | | 9 (25) |
| *Emotion & Body* | | | *18 (40)* |
| Laboured breathing | | | 7 (19) |
| Terror or Panic | | | 6 (17) |
| 'The rush' | | | 5 (14) |
| Anxiety or Fear (Onset) | | | 4 (11) |
| Pain / Torture (Onset) | | | 2 (6) |
| Trapped / Powerless | | | 1 (3) |
| Body expanding / melting | | | 1 (3) |
| *Space-time shifts* | | | *10 (28)* |
| Reality breaking down (Open eye; Onset) | | | 4 (11) |
| Immediately elsewhere | | | 4 (11) |
|  | | |  |
| ***Bodily*** | | |  |
| *Pleasurable* | | | *10 (28)* |
| Ecstasy | | | 4 (11) |
| Pain relief | | | 2 (6) |
| Fusion (with partner) | | | 2 (6) |
| Rooted | | | 1 (3) |
| Stretching urge | | | 1 (3) |
| Warmth | | | 1 (3) |
| Post-orgasmic state | | | 1 (3) |
| *Neutral / Both* | | | *8 (22)* |
| Religious hand-signs | | | 3 (8) |
| Vibrating or (Subjective) Convulsion | | | 3 (8) |
| ‘Phytanthropy’ | | | 1 (3) |
| Falling sensation | | | 1 (3) |
| *Uncomfortable* | | | *9 (25)* |
| Heaviness | | | 1 (3) |
| Paralysis (Subjective) | | | 1 (3) |
| Sensitivity | | | 1 (3) |
|  | | |  |
| ***Emotional*** | | |  |
| *Positive* | | | *34 (94)* |
| Peace / Pleasantness | | | 16 (44) |
| Profundity & Beauty | | | 14 (39) |
| Familiarity | | | 11 (31) |
| Gratefulness | | | 8 (22) |
| Humour & Hilarity | | | 7 (19) |
| Healing | | | 5 (14) |
| Release or Relief | | | 4 (11) |
| Humility | | | 3 (8) |
| Gentleness | | | 2 (6) |
| Womb-like | | | 1 (3) |
| *Neither / Both* | | | *21 (58)* |
| Extreme intensity, or Overwhelm | | | 21 (58) |
| Letting go / Detachment | | | 3 (8) |
| Wash of emotion | | | 2 (6) |
| Ambivalence | | | 1 (3) |
| Infantile regression | | | 1 (3) |
| Disappointment | | | 1 (3) |
| *Challenging* | | | *7 (19)* |
| Anxiety or Fear (During) | | | 3 (8) |
| Confusion | | | 2 (6) |
| Fear of letting go | | | 1 (3) |
| Grief (from own death) | | | 1 (3) |
| Guilt | | | 1 (3) |
| Pain / Torture (During) | | | 1 (3) |
| Personal struggles | | | 1 (3) |
| Traumatic re-experience | | | 1 (3) |
|  | | |  |
| ***Sensorial*** | | |  |
| *Open-eye* | | | *11 (31)* |
| Other open-eye visuals | | | 7 (19) |
| Reality breaking down or Pixilation (During) | | | 3 (8) |
| Clairvoyant-like | | | 2 (6) |
| Energy flow | | | 1 (3) |
| Skulls | | | 1 (3) |
| Depth-perception | | | 1 (3) |
| *Visual* | | | *27 (75)* |
| Colourfulness | | | 13 (36) |
| Fractals | | | 11 (31) |
| Hyperdimensionality | | | 8 (22) |
| *Cross-modal & Other* | | | *14 (39)* |
| Synaesthesia | | | 10 (28) |
| Sound (During) | | | 4 (11) |
| Sensitivity | | | 3 (8) |
| Audio slowing | | | 1 (3) |
| Tactile | | | 1 (3) |
|  | | |  |
| ***Psychological*** | | |  |
| *Memory & Language* | | | *31 (86)* |
| Temporary memory loss (within experience) | | | 15 (42) |
| Partial recall loss (after experience) | | | 12 (33) |
| Looping & Intrusive thought | | | 4 (11) |
| ‘Schizophrenia’-like | | | 1 (3) |
| *Awareness & Sense of Self* | | | *18 (50)* |
| Aware of surroundings | | | 9 (25) |
| Unaware of surroundings | | | 8 (22) |
| *Time distortions* | | | *13 (36)* |
| Time contraction | | | 4 (11) |
| Other distortions | | | 3 (8) |

**SM 4.**

## **Table 5: Table of all super-ordinate, mid-level and subthemes**

| **Encounter with Death** |  |  |
| --- | --- | --- |
|  | Clarificatory notes | No. Interviews /36 (%) |
| ***Near-Death Experience, Death & Birth*** |  |  |
| *Typical NDE features* |  | *34 (94)* |
| Disembodiment |  | 19 (53) |
| Translocation elsewhere | e.g. Sucked, Magnetised | 12 (33) |
| Tunnel-like structures (During) |  | 10 (28) |
| Bright light(s) |  | 9 (25) |
| Sense of dying (During) |  | 8 (22) |
| Tunnel-like structures (At onset) |  | 7 (19) |
| Sense of dying (At onset) |  | 6 (17) |
| 'Limbo-land' / The Void | Between worlds, Waiting room-like | 4 (11) |
| Light Being-esque |  | 3 (8) |
| Deceased family |  | 2 (6) |
| Hyper-empathy | Assuming the perspectives of others | 2 (6) |
| Life-review-like |  | 2 (6) |
| *Less typical motifs* |  | *14 (39)* |
| Birth imagery or Being born |  | 5 (14) |
| Death imagery & Skulls |  | 5 (14) |
| Sounds (At onset) | e.g. Ringing, Rushing | 3 (8) |
| Reduced fear of death (after-effect) |  | 3 (8) |
| Etheric body |  | 2 (6) |
| Being in prime |  | 2 (6) |
| Partner lying dead | Participant pairs who experienced the DMT lying beside each other, in which one partner envisioned the other as seemingly dead | 2 (6) |
| Dark & earthy space |  | 1 (3) |
| ‘Placebo-death experience’ |  | 1 (3) |
| Psyching the Psychopomps |  | 1 (3) |
| Scenes on screens |  | 1 (3) |
| Repulsed by body |  | 1 (3) |
|  |  |  |
| ***The Mystical Experience*** |  |  |
| *Typical mystical dimensions* |  | *29 (81)* |
| Ego death | c.f. Temporary memory loss | 11 (31) |
| Ineffability | c.f. Difficulty expressing | 11 (31) |
| Deep positive mood | Sometimes including Love; c.f. Pleasantness; or Ecstasy | 11 (31) |
| Unitive / Oneness | c.f. Connection | 9 (25) |
| Noetic | c.f. Revelation | 8 (22) |
| Time & Space transcendence | c.f. *Time distortion; or* Translocation | 8 (22) |
| Paradoxic resolution |  | 6 (17) |
| Sacredness | c.f. Profundity/Beauty | 2 (6) |
| *Entheogenesis* | *The profound feeling of identifying with the Divine, such as being God or Creating the universe* | *3 (8)* |

**SM 5.**

## **Graphic of all super-ordinate and mid-level themes (top-left) – including those of Encounter with the Other & Self (Michael et al., 2021, 2023**)


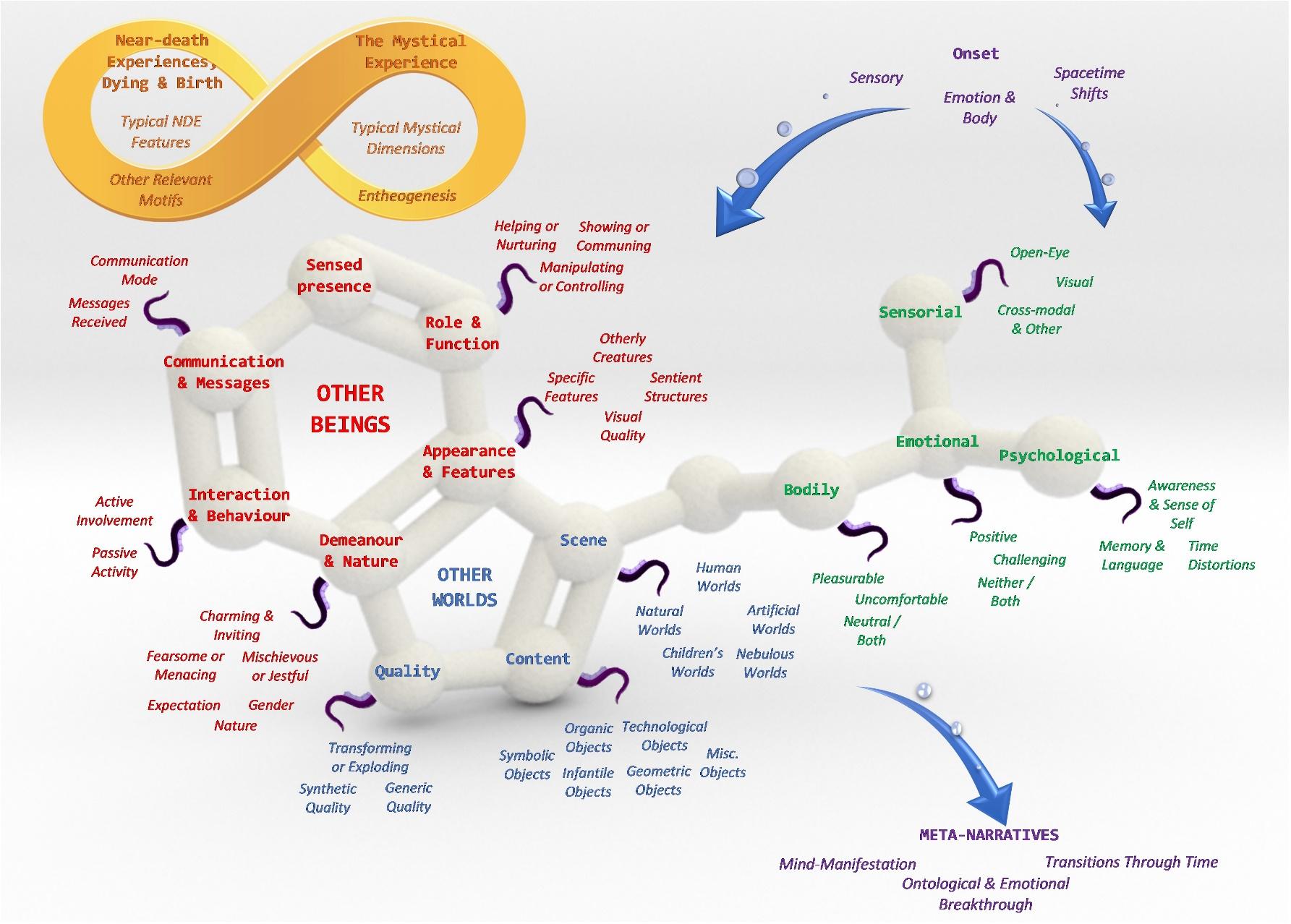


**SM 6.**

## **DMT participants reporting NDE-like experiences**

***LG***

*LG,* curiously, reports that he felt (purely subjectively) that he was suffering a cardiac arrest, including the ensuing attempts at resuscitation, mirroring a scene expected of a genuine near-death experiencer. Following this, perhaps as a result, a number of NDE themes are recounted:

**Sense of dying (both *at onset*, and *during*)**

Also first touched on here is the singular example of *Grief,* not for another, but for himself given his conviction of his death, and empathising with the grief of his romantic partner.

“So I died… I felt as though I left earth behind. And that she [partner] was gonna have to deal with that… it was like, normal life, but that I’d had a fucking heart attack… I was like ‘Fuck! This is it, she’s gonna be devastated’. But then…I was going ‘No, No…this is the nature of reality…and it’s not always cushty, you don’t know what’s the round the corner, so saver whatever is going on at any point’. But then I was like, so I’m dead, I *am* dead now…

I felt as though you guys…you were standing around, you were looking down at me, trying to resuscitate me. You were like ‘Fuck, fuck, no he’s gonna die’, and I was like ‘Shit, this is it, this is it!’. Then *boom*, gone, I was just into this other plane”

Here, as well, is the sense of *Translocation,* via an initial sinking and then sudden zooming, elsewhere.

“The first thing was…being on my back, my vision was as though i was sinking… and I could see you guys…trying to revive me. And then ‘No, fuck, this is it, there's nothing you can do’… I was trying to fight it, breathe slowly…but it was sinking more and more, and then 'this is it now, you’re going to have to embrace it''. And then it just switched, and I was like *vrrrooommhh* into this ethereal aura of whatever icicle thing…

It was like it was going in 2 directions, one was this physiological shit that was going on, and one was this plummeting down of 'this is your sprit going somewhere else', your essence or soul or whatever you wanna call it, *choooh*, and they were fragmenting off in different directions. And as soon as my body died, it just went *vroom* straight into this other…plane”

The preservation of ego (see Michael et al, *in submission*), versus its dissolution, is alluded to here – which is congruent with the majority of NDE reports.

“I was still me, my thought processes still felt the same… I was in the icicle, and slowly I was like 'I am dead, but my ego was still here, so death is like, so it’s not that big a deal!’... the comprehension that everything is ever-changing, and your energy is still going somewhere else”

**Etheric body**

*LG* additionally described witnessing himself as a form of ‘etheric body’ – which while certainly observable in NDE accounts, does not seem to be especially common. This is reported by 2 individuals, but is technically subsumed under ‘*Less typical NDE motifs*’ (see below):

“I had my body, but it wasn’t my physical form; it was like an energetic form, it was the same, the same age and everything, but I was semi-transparent

*Interviewer: A kind of spirit body?*

Yeah! And I was just there like What the fuck is this, I couldn’t even pay attention to the surroundings that much because I was so distressed by the fact that I was dead (laughter)”

Peculiarly, he mentions an autoscopy, not of his own (actual) body, but of this new form of ‘body’, accompanied by a duality of his own mind. The pain of his unique grief is also re-expressed:

“It was weird, I could kind of see myself, and I was myself at the same time. It was almost like two perspectives going, my internal dialogue and also viewing my face, like the shellshock, the magnitude of what’s just happened… There was almost like a feeling of, I guess it was grief that set in, grief, but not for me, but for her…

I was like, ‘No everything is so fucking amazing between us’, I love her, like, fucking more than anything, it was like how could I do this, how could I leave her to deal with the pain of my loss, and what it would be like to be the other way around!?… it’s just the sorrow of not being able to see that person and kiss them. I was just fucking devastated…

I’m in this [other plane] for eternity… I didn’t mind I was dead, it was the fact I'd never get to see her again…

it was like I was experiencing this internal dialogue form [one] point of view, like looking outward; but then there was like a parallel perspective going on, and it was looking at my face…watching my facial expressions. But I could experience my thought processes from both perspectives almost. One was kind of like the first [part of] the experience of going through death and acceptance, then the other was an observation point of view of ‘this is what it looks like to experience that emotion’”

**Tunnel-like structures (*at onset,* and *during*)**

Importantly, *LG* announces this particular experience to be “not like the ‘tunnelly ones’, traveling towards it”, clearly alluding to the commonly-known trope of moving through a tunnel, but instead feeling an artificial, “vertical column” to be moving around himself. Also identifiable here is the feeling of levitation, and messages communicated by alien-like, and reassuring entities (see Michael et al, *in submission;* Michael et al, 2021):

“But then I was like, so I’m dead, I am dead now. Then I was looking around in this plane. It was blue, like waterfalls coming down around me…

Then *boom*, gone, I was…with these waterfall cascading things, very digital in feel…

I was trapped in this…vertical column and I was in it, like an icicle, like I was in the middle of an icicle. I was looking around and it was kind of almost digitally *Matrix*-esque, like blue swirling around me going round to the ground… there were some forms, like faces, like coming out of this *Matrix*-esque mesh and looking towards me… [saying] ‘No, you got to accept what’s happened here now…This is the nature of how energy works – its gonna revert back on itself at some point’…

these kind of feminine forms…kind of *Avatar*-esque, like cartoony, blue, braided hair, kind of very high definition though…sharp… I've had this with ayahuasca, [entities] turning up like this, giving you some sort of expression… welcoming, almost slightly seductive, not in a sexual way, like ‘it’s all good’”

In a subsequent communication to the author (PM) by *LG,* the participant provided an elaboration on his experience: He reported an “acceleration” within the tunnel; he additionally referred to the vertical, holographic/digital ‘icicle’ and ‘waterfall’-like tunnel’ as a “cone”; he experienced auditory-visual synaesthesia as he spoke, where the words would transform into “Shapibo symbols” or “Egyptian hieroglyphs” onto the walls of the tunnel; and his etheric body appeared to have “extra dimensionality”. The beings he encountered were “feline, and revolving”, and gave him the understanding that the place he was in was “a place of sensual pleasure”; they demonstrated an initial shock to his appearance in their space, followed by a recognition, communicating that “You are not meant to be here, this is the place you come when you die”, where they attempted to “calm me down”, conveying that “You have to accept this [i.e. that you are dead]”. There was a sensation of having “been here before”.

As such, these extra features not only highlight even further the classically DMT-like nature of the content, but entail features usually absent from the NDE including symbols/glyphs or a hyperdimensional body. The use of ‘cone’, as well as ‘icicle’ or ‘waterfall’, to describe the tunnel of light is interesting, which echoes descriptions used by clients of hypnotherapists regressing to ‘intermission’ states (purportedly between lives), including “lying on a table under a ‘dome’ surrounded by bright light”, or “group souls” to which persons return to at death being “surrounded by ‘bright cones’ above and all around us”, funneling energy “as a waterfall in a spreading circle” (Shushan, 2022; p 146, p 151). The startled entities’ message that he was, erroneously, in the realm of the dead concretizes the theme (see original manuscript) of ‘psyching the psychopomp’, where the participant stresses this features’ role in his conviction of being in said realm. To extend this important theme further, another anonymous presenter at the 2022 Tyringham Initiative conference, Broughton Hall, additionally experienced during not only one DMT experience entities which he overheard to say “There's a human! He's not dead yet, leave him alone! Be careful, if we fiddle with them something might happen. He'll get here eventually”.

***EM***

**Sense of dying (*At onset,* and *During*)**

The DMT experience of *EM* also begins with the sensation of dying. But, of note, for her at least it is one generated only by virtue of the inhalation of the substance itself. Also evident throughout the excerpts below is the sense of familiarity, present even in her first uses of DMT, such as the familiarity associated with death – which is a common feeling in true NDEs:

“So I was faced with this familiar feeling of like, when you smoke DMT, it feels like…a very familiar sensation; it’s like between birth and death, that always keeps occurring

*Interviewer: Was that your prior, usual experience?*

Yeah but…I’m firmly convinced that [the feeling of dying] only comes because of the feeling of intoxication that I get from actually smoking. Because you feel like you’re actually putting something into your body, and literally it’s a feeling of intoxication

*Interviewer: Intoxication to the point of potentially dying? …a poising perhaps?*

Exactly! Proper intoxication”

**Disembodiment**

Crucially, *EM* here is emphatic that though she lost all awareness of her body, it was “not like an OBE” as in classical near-death experiences. One consistency though with NDEs, akin to *LG* above, is her eventual allusion to a preservation of her selfhood as distinct from her surroundings:

“I was a very compact point of consciousness… my body was more like a ‘ball’ or something, but I definitely wasn’t aware of like a human body or something…

So it was definitely not like an OBE, definitely not. I cant necessarily say if I was returning [to] or ever exited my body, I don’t think I've ever done that. I think at some point my conscious voluntary thoughts were stopped and I entered this new world, but I never at any point felt like I had left my body…

I was still that contained point of consciousness, so never at any point I felt like I moved from here, I was exactly where you guys left me at all times… mentally I was definitely somewhere else. But I don’t feel like I left my body…

I was an induvial presence, I was separate from [the entities]”

**Tunnel-like structures (*At onset,* and *During*)**

Again, similar to *LG,* her tunnel structures appeared in the opening stage of her experience, but were ever-present throughout, and moved themselves around – compared to NDEs wherein the tunnel feature is simply a transitioning phase (with more developed NDEs) to a wider experience, and the experient moving through. Their nature as transforming, fluxing, colourful, and paradoxical (see Michael et al, 2021) is also stereotypically psychedelic (though their sensed reality and familiarity is NDE consistent):

“So there's this massive, massive building… there's like all these different shapes and tunnels… you can go through different rooms and…every single room is basically like a Pandora box. But it’s not a room per se… It’s just like full of different shapes and tunnels and morphing rainbow stuff everywhere. So every single thing has the ability of turning into a tunnel. So you’re just going through all this stuff, then at some point I realised the room I was in was kind of morphing and I was into (?) one of those tunnels and the tunnels were splitting, and it was fun!... you’re trapped in this never-ending tunnel that moves around but you’re still fine…because you…just go with the flow…

like ventilation pipes, they’re very messy, they don’t seem to make much sense where everything is… So you know those spirals, when it’s wider here and it goes narrower, and it was spirally – you know, the barber shops, like that everywhere… It was all colours, like lollipops…

when the actual tunnels appeared – [they were] moving around…twisting in the other direction… but there are different layers of the tunnel, and all my friends [entities] were running around in between them and coming through them… everything was moving all the time! Yeah, a lot of movement, very dynamic process… But in a very organic way…

It was like the tunnel was moving me…I definitely didn’t put in any effort… So I was both consciously travelling through them and they were moving around…

they were pretty real, they were pretty damn real structures… There was definitely travel at first through tunnels. Very familiar tunnels… in DMT, all the time, the onset feeling is filled with familiarity”

**Bright light(s) (and Entities and Other worlds)**

When describing her “building”, which was sentient and transforming also, the lighting described is also reminiscent of NDEs. And her otherworldly, intricate garden scenery is a natural landscape exceptionally common throughout near-death reports (incidentally, all such elements are in *RH’s* 1^st^ trip, Michael et al, 2021):

“It definitely seemed like…a construction that has a mind of its own in a way, because it was moving around and everything was changing inside. It was very well-lit…there was a lot of natural light coming in – but there were no windows, which was absolutely crazy…

It had gardens as well…it has rivers and everything, it was just like one of those palaces form ancient Babylonian gardens or something, it was beautiful. And every single bit of the wall, you know, like mosques have all these patterns and all these beautiful glass like stuff inside, that everywhere, on every single little bit”

She also illustrates in her experience benign, protecting entities of humanoid form – congruent with NDEs – yet again, their visual manifestation of featureless “dancing harlequins” is classic of DMT (see Michael et al, 2021), and absent in NDEs:

“It felt very communal…it was so beautiful, and these people were so nice, all these aliens around… All those, there must have been around 50 entities and they all seemed to be my friends, it was so nice to have all this community of harlequins…

So you know those costumes that people put on when they don’t have their face, that’s just like a colour… A body suit… they’re just going around like a lot of spinny things, they were watering certain bits… And I felt so taken care of it was absolutely amazing, and there were shit-tonnes of them!... like, fixing bits and pieces, but it was like a dance”

**Other NDE-like cases to lesser degrees**

Several other interviews are also redolent, though not quite as strongly, of the near-death experience literature. *RH (2)*’s themes of dying at first, its terrifying nature; entry to an inescapable void; and ultimate encounter with a being of light have all already been discussed in the above section on *Qualitative comparison. RH (1)*, not dissimilarly, contained within it themes of initially dying, its disturbing nature; the void i.e. the “terror” of being “trapped” in a “waiting room” that “doesn’t make sense”, which is, notably, progressed through upon his mantra, “Die to it”; confronting fearsome, disembodied eyes i.e. “Something conscious, which was red” with a “Crazy number of eyes” and was “really scary”; a light-being-esque “feminine presence”, looking “metallic, rose, gold, silver”, “shining”, and “bigger than a planet”, and communicating the message “‘We thank you’”; “light space, nothing except light space. Really comfortable, really beautiful – it felt like home”; and finally a “more than…5D… garden, of extraordinary beauty”. Progressively less evocative of NDE tropes includes *AN’s* descriptions of bodily and ego dissolution (not a sense of dying, *per se*); entry to a dark, earthy space i.e. “submerged… in the depths” in a “churning of deep purples and blues…crazy mud colours” with “rooted…dark…feelings”; from which a “ladder from above and below” arose, made of “cogs…joined”, and “met in the middle”, finally “twisting into” a “golden light”. Thus, *AN’s* experience echoes the rebirth motif or ascension from a primitive state into one of beautiful light (e.g. see Michael et al, *in submission,* on Alexander, 2012). *TC’s* trip entailed a transcending of the ego; being “sucked” into a “contracting, expanding… tunnel-like thing” or “void”; and then “flying through” light and natural landscapes i.e. “bright…scenery, like mountains”, “like a cave, beautifully illuminated”. *AV’s* account of a sense of dying; her being encompassed by a sentient sun-like mandala was discussed above (*Qualitative comparison*); which communicated a “telepathic message” in “a voice, telling me to ‘surrender, this is a sacred space, you’re being held, you are safe’”, again evoking the sense of sacredness. Lastly, *JB* states an ego-death and “melting into the universe”; a “rushing, breaking-through, noise…like a *fhoo*”; while “going through… a red… geometric… tunnel”; and ultimately a union with a “Oneness, an Everything entity, a Universal entity of just, you know, God or whatever!”

Perhaps of interest, is that the majority of these, including *LG* and *EM,* involve the sense of dying at the very onset, or if not, the reporting of an ego-death – which may in itself partially account for the ensuing NDE-like tropes, if creating a suggestion for death-pertaining motifs. Saliently, in terms of the qualitative content of, at least these particular discussed themes, of these partially NDE-like DMT trips, it appears consistent with authentic NDEs. Except for, for instance, beings of light witnessed as ‘synapses’ (*RH 2*) or ‘mandalas’ (*AV*), the intimidating eye-adorned being (*RH* 1, however, see Luke, 2008 on the cross-culturally and trans-temporally recurrent portrayal of many-eyed demonic entities associated with death, specifically), cog-composed ladders (*AN*), and perhaps too, throbbing (*TC*) and geometric (*JB*) tunnels, and hyperdimensionality (*RH 1*) – all of which being more expected in DMT versus NDEs. In addition, while *AV* and *JB’s* experiences were highly mystical in nature (see *Supplementary Material,* ‘Mystical Experience’), many others, namely the most NDE-like *LG* and *EM,* lacked such mystical dimensions, where NDEs (see *Quantitative,* and *Content Comparison,* main paper) tend to show preservation of ego and independence from the environment. Though, naturally, mystical DMT trips will mirror mystical NDEs.

**SM 7.**

## **Narrative of Idiosyncratic NDE (Cassol et al, 2018)**

*Patient DTO7*

It was during one of these [suicide] attempts that I experienced what I call a ‘DIVINE ENCOUNTER EXPERIENCE’ in which GOD manifested himself to me and my life changed completely and instantly… On many occasions I had fallen to my knees and with my arms outstretched to heaven asked God to come and find me in order to free me from my suffering… I swallowed what remained of the pill bottle and drank half a bottle of cognac… All of a sudden I woke up on a stretcher in intensive care…

I was terribly thirsty and I sat in my bed and asked the nurse for a drink. She couldn't hear me, she wouldn't answer me! I spoke louder to no effect, and then she turned around and spoke to another nurse behind me, something unrelated to me. I thought she was ignoring me. Exasperated, I decided to get up to have a drink and got out of bed. I was standing next to the stretcher and looking at the stretcher, someone was lying in it: ME! I knew I was out of my body but didn't want to believe it and I repeated my request to drink while standing right next to the nurse and I SHOUTED “DRINK". Still no answer! Then, like a thick black veil, like a light going out, this "blackness" fell on the room. At that point the nurse turned around and looked at the cardiogram and said to someone "we're losing him!" These were the last words I had time to hear because the ‘blackness’ totally enveloped me, cutting me off from everything. I couldn't see anything, I couldn't hear anything, and I was afraid as one might be afraid in total darkness in an unknown place. Then I cried out in fear, "I want light!" Then a MINUSCULE dot appeared in the distance, as when looking at a star in the sky. From this point came a ray of light, thin and opening more and more in front of me, a white light, "IMMACULATE, PURE AND BRILLIANT WITH AN ENERGY IN WHICH I FELT AN UNCONDITIONAL AND BOUNDLESS LOVE". This light reached me and enveloped me just enough so that I could ‘MOVE FORWARD’ into this light. I did not walk forward but felt as if I was drawn into that light, and only my will moved me forward… I continued to advance in this light AND I KNEW THAT IN DOING SO I WAS LEAVING MY LIFE BEHIND HEADING TOWARDS WHAT MIGHT BE CALLED ‘HEAVEN’." Then one of these people raised their arm and hand in a stop sign and said, "WHERE ARE YOU GOING?" I answered: "I am going to heaven! I cannot live on earth, I am unable to do so, my suffering is so great that I can’t bear it anymore!" The person answered: "STOP, YOU CANNOT ADVANCE INTO THIS LIGHT, YOU WILL DEFILE EVERYTHING, YOU KILLED YOURSELF!" Then this person spoke to the others again and said, "YOU WILL RETURN TO YOUR BODY AND GO COMPLETE YOUR ‘MISSION’. I said, "I can't do it alone, I can't do it.” The person said to me: “WE WILL HELP YOU.” Then I felt ‘pushed’ by force in this light towards my body. I sat up straight in my bed and the nurse was looking at me, and I said, "I HAVE SEEN GOD!" Then I fell back into a deep sleep and awoke the next morning. The next day I asked the nurse if she had heard me asking her for water to drink? She replied: "YOU NEVER SAID A WORD, YOU WERE IN THE COMA AND WE ALMOST LOST YOU. YOU GOT UP AND SAID: ‘I’VE SEEN GOD’"…

[Later, in Summer of 1986, he reports a depressive episode and not eating for several days, and upon going to sleep reports the following]

I awoke and it seemed to me that the shelves in the bookcase next to my bed were ‘sinking’ into the ground. There was something weird and I knew I was AWAKE. Then I felt an unpleasant sensation as if my back were pressed up against something and my body half-penetrated this physical obstacle which turned out to be the ceiling of my room. It wasn't the bookcase that was sinking into the ground, it was I who was rising up to the ceiling. And now I was half-way into the ceiling and I found that VERY unpleasant. I wanted to turn around to face upwards as one would move one’s physical body, but it didn't work. I got angry and I ORDERED that I wanted to turn around and I turned to face the ceiling, which was VERY uncomfortable. I had understood that the movement of the body was done by means of THE WILL; so I ORDERED to be standing next to my bed, which was done. I saw the room in the same way as usual and everything was REAL, NOT AS IN A DREAM WHERE EVERYTHING IS INTANGIBLE, A LITTLE BLURRY, ILLOGICAL. HERE EVERYTHING WAS NORMAL AND I UNDERSTOOD THAT I WAS OUT OF MY BODY… A cord of light connected me to my body on the bed and was connected at the navel. Then I approached a wall and as close as possible, I looked and saw that the wall was ‘LIVING’; I could see the atoms moving in the wall. I then passed my hand through the wall to find out if I could get out of the room and my hand sunk into the wall but it caused me a VERY UNPLEASANT feeling and, excuse the expression, but it was as if I had put my hand in a pile of hot (or warm) excrement... Then I walked around the room and wherever I went, this cord spread and I could see it drawing a line in the room in my wake. There was a book on the table and I stepped forward to ‘READ’ what was on the cover BUT I WAS UNABLE; IT WAS LIKE I COULD NO LONGER READ. Letters and text are a ‘MATERIAL’ concept, and I understood that on ‘the other side’, we abandoned this concept.

Then I turned around and looked into the corner of the room. I saw a tiny point of light appear but BLACK (if you can conceive the idea of a BLACK light), and as in the hospital…this point grew to become a small mass, a mixture of a slightly viscous-looking material and a gaseous cloud. Then in this mass, an imprecise shape, a face took shape and the more I watched this thing form the more a ‘human’ face appeared until this face that appeared to me was MY OWN. This face ‘SHONE’ with a BLACK light from which rays were escaping, but I ‘FELT’ a ‘negative’ energy emanate from it. Then this face says to me: "COME WITH ME, WE CAN CRY TOGETHER FOR ETERNITY. WE WILL BE ABLE TO RELIEVE OUR SUFFERING THUS (I must say here that no words were exchanged and that I express here in ‘WORDS’ what was communicated only by thought)… I felt a negative energy emanating from this entity and I mistrusted it, as I felt that it wanted to ‘deceive’ me. It wanted to lure me to relieve itself of its own suffering (what does one most want when one is sad? A person to ‘share’ this sadness). I felt that if I said ‘yes’ to this entity and agreed to go with it, it was in death that I would be deciding to go, and I had a choice to make: DECIDE TO LIVE OR TO DIE.

Then I saw a kind of river where all kinds of other entities were ‘swimming’ or ‘floating’, ALL SUFFERING, CRYING, IN A SORT OF “LOWER ASTRAL" [REALM] WHERE ALL THE MOST BESTIAL, FRENZIED OR UNFORMED THOUGHTS MATERIALIZED AND FORMED THIS RIVER IN WHICH THESE LOST ‘SOULS’ ‘BATHED’.IDEAS HAD FORMS; SOME WERE LIKE LONG, SHAPELESS SNAKES THAT PENETRATE YOUR BODY, OTHERS WHINED LIKE AN EMBRYO OF ‘SOMETHING’ NOT YET BORN AND WANDERED AIMLESSLY, ALONE WITH THEIR SUFFERINGS WHICH THEY CEASELESSLY SOUGHT TO RELIEVE BY SEIZING THE ATTENTION OF OTHER ENTITIES. MY GOD, THAT WAS INTOLERABLE, UNBEARABLE; AND I UNDERSTOOD THAT SUICIDE IS NOT A ‘SOLUTION’ BECAUSE WHEN ONE COMMITS SUICIDE ONE REMAINS IN THE SAME STATE ONE IS IN AT THE TIME OF ONE’S DEATH AND THAT ONE IS, FOR AN INDETERMINATE TIME BY GOD ONLY KNOWN, CONDEMNED TO ‘WANDER’ IN THIS LOWER ASTRAL [REALM] WITH THESE OTHER ENTITIES AND THAT OUR SUFFERING BECOMES THUS PERHAPS ETERNAL (GOD WILLING). SUICIDE IS A ‘TRAP’ BECAUSE IT DOES NOT SOLVE ANYTHING AND IT EVEN MAKES THINGS WORSE BECAUSE THIS STATE BECOMES PERPETUAL. SUICIDE IS THE GREATEST SIN AGAINST GOD BECAUSE IT IS TO DENY LIFE ITSELF AND TO DENY GOD HIMSELF! I understood all this and I believe that this lower astral [realm] could be what we call ‘HELL’! AND IN MY SOUL AND CONSCIENCE I COULD NOT ACCEPT THIS AND DEEP DOWN IN MY SOUL I DESIRED TO ‘LIVE’, SO I SAY "NO" TO THIS ENTITY AND THAT SUFFICED TO REPEL IT. IT ‘DEMATERIALIZED’ AND DISAPPEARED INTO ITS VISCOUS BLACK CLOUD. Then a light coming from above penetrated the top of my head and descended into me. As it descended, my whole (astral) body relaxed completely and when that light reached the level of my heart, I felt a kind of explosion of PURE LOVE, UNCONDITIONAL, A CONCENTRATED LOVE, and I felt bathed in this love. Then this huge hand coming from above penetrated the top of my head and this hand made its way among my ideas, my emotions, my feelings, packing things down and pushing others aside, looking for something ‘SPECIFIC’. I felt invaded by something totally unknown, and I was totally powerless, so I was scared and I felt somewhat ‘violated’ in my inner self, not physically, but in my soul and I asked, "WHO ARE YOU?" and a voice answered me: "I AM THE HOLY SPIRIT, I AM A FORCE OF LOVE, I CONTROL ALL YOUR ESSENTIAL FUNCTIONS, YOU HAVE NOTHING TO FEAR. " I felt that this force was ‘rummaging’ around my soul and I asked, "WHAT ARE YOU DOING?" The voice replied: "I AM GOING TO TAKE AWAY YOUR SUFFERING, YOU WILL NOT NEED IT ANY LONGER.” Then I felt this hand grab something in me and throw it away.

The light disappeared and I stood there, stunned, and after what had just happened I became afraid not knowing what might still happen to me. The desire to return to my body became urgent and I gave in a little to panic. I felt like I was "running" and "throwing" myself into my body, not caring what might happen. Just before I re-entered my body, I was somewhere in between my body and outside, I felt at that moment a kind of click at the level of my kidneys and a crack at the nape of my neck and an invisible hand guided me into my body. …

Then little by little my thoughts came back to me; I was ‘filled’ with an energy, of such intense strength that I could have run miles to spend it, but physically my strength prevented me from doing so and I had to hold onto the walls to go and sit in a chair. All my limbs trembled and inwardly I was so supercharged with this immense energy that I sat for a long time waiting for that energy to dissipate. I KNEW SOMETHING HAD JUST HAPPENED BEYOND MY COMPREHENSION AND I MUST SAY THAT I NEVER AGAIN EXPERIENCED THE URGE TO CONSUME ANY DRUG WHATSOEVER AFTER THAT. WHAT ‘GOD'S HAND’ HAD TAKEN AWAY FROM MY SOUL, MY ‘SUFFERING’ HAD FOREVER LEFT ME. IT CHANGED MY WHOLE LIFE AND SOON AFTER I MET MY SOULMATE, THIS WOMAN WHOM I LOVE AND WHO IS STILL WITH ME TODAY, THE MOST FORTUITOUS MEETING OF WHOM CAN ONLY HAVE BEEN GOD'S PLAN. For 15 years now, I have not felt the urge to consume and I have understood that the ‘MISSION’ entrusted to me was to HELP THE PEOPLE THAT LIVE AS I DID IN THE HELL OF DRUGS AND TO SHARE MY EXPERIENCE TO INFORM ON THE REALITY OF SUICIDE, PEOPLE WHO BELIEVE THEY HAVE FOUND A SOLUTION TO THEIR SUFFERING IN THIS WAY, AND ALSO TO COMMUNICATE MY EXPERIENCE TO TELL PEOPLE THAT GOD EXISTS AND THAT DEATH IS THE END OF NOTHING, THAT SOMETHING EXISTS BEYOND DEATH, THAT WE ALL HAVE A VERY IMPORTANT ‘MISSION’ TO FULFILL IN THIS LIFE.

**SM 8.**

## **Indicative Questions of Semi-Structured Interview (with initial prompts)**

- Please can you describe your DMT experience as best and fully as you can, and as chronologically as you can. Please use all your own words, and try to break down your experience into its detailed elements, while avoiding using terms and concepts from other people or popular culture

- What is the first thing you remember?

- How did your body feel during the experience?

How did your awareness of your body change?

- Did you see any geometric, fractal or entoptic patterns?

Where they colourful, or moving? What was their dimensionality?

- Where did you ‘go’ in your experience?

What did the scene look like?

- Did you have any encounters with other beings in your experience?

What did they look like? What was their approach toward you? Did they communicate with you? Did they interact with you?

- Did you receive any information, or have any particular insights or understanding?

- How was your sense of time affected during the experience?

How long did the experience seem to last? (Versus how long it *did* last)

- How pleasurable was the experience?

What other emotions did you have?

- What was your sense of self like?

In relation to the world around you?

- On a scale from 1-10 where 10 is the most intense and 1 is normal intensity of experience,

how intense did your experience get?

**SM 9.**

## **Canonical NDE Themes – Different Content – Life Review-like**

*SH’s verbatim quote*

“It just felt so playful. And I was thinking, What’s the message!? She was like ‘Don’t be so stupid, there is no message’. And I just couldn't stop laughing! She was just like ‘It’s just a fucking game, just play it! It’s an infinite game and you can make up the rules!… it’s here to have fun, you can’t win it, you can’t lose it, you’re just in it... And she was playing with me… I had these questions in the back of my mind about my life. And it was just going ‘It doesn’t matter!! Just be here now!’ And it felt really like it was Shakti just putting on this performance for me like, ‘Isn’t it fucking amazing, like existence, just Woooo!’. It was beautiful and…the whole of life felt so silly. And in a nice way, not like Oh it’s silly and pointless, it was like ‘It’s silly, and that's it!!! Like, Enjoy it, Wahooo!’ Like life’s just a fair-ride, a rollercoaster, something to enjoy… Like everything was everything; all is one, it’s all just the same stuff, putting on a show, and we make these differentiations but they’re not really there”

That the message to DMT participants SH & AF was also given by flirtatious, feminine guides makes these encounters of striking comparability to the Epic of Gilgamesh, which has actually been speculated as the first NDE documentation (Shushan, 2022). Specifically, the words, as quoted by Zaleski (1987), of the barmaid Siduri, who was “at once temptress and wise woman” may be interpreted to contain both such elements of hedonistic abandon as well as commands of responsibility, where she counselled Gilgamesh, after he “tunnels through the underground realm where the sun travels at night”, to relinquish his obsessive quest for the antidote to death:

“Thou, Gilgamesh, let full be they belly,

Make thou merry by day and by night.

Of each day make though a feast of rejoicing,

Day and night dance thou and play!…

Pay head to the little one that holds onto thy hand,

Let thy spouse delight in thy bosom!

For this is the task of [mankind]!”

**SM 10.**

## **Features not illustrated in Table 2**

For example, as regards the ‘roles’ and ‘demeanours’ of the entities, as coded in Michael et al’s (9) prior DMT analysis, some entities of the NDE narratives still may have included soothing, healing, or orchestrating beings, who may be benign, beautiful, powerful or familiar. Other themes may have been less prevalent (or absent). In regard to ‘emotional’ feelings or ‘bodily’ sensations (10), due to their being repeated themes across different categories in Cassol et al (27), generating frequencies and thus comparison with the DMT reports was impossible. These themes, however, were spanning absence of body, thirst, extreme cold; positive emotions of happiness and serenity, well-being, confidence, release, astonishment, amazement, absence of pain, curiosity; and challenging ones of presence of pain, fear, unbearable sadness, exasperation; and finally indifference. All such themes (except thirst, cold, pain, and indifference) were present in the DMT reports (to degrees observable in Table 2 above) – where, notably, the comparative prevalence of positive to negative emotions was not significantly dissimilar between the DMT and NDE states, with approximately 4 DMT participants, and – approximately – 3 NDE narratives suggesting significantly distressing episodes.

In respect to the DMT themes out of all those unhighlighted in Table 2, and thus coded across the NDE narratives yet legitimately not present therein, many will be paid special attention to here. For example (from themes in Michael et al (9)), all entities characterizable as clown-like, alien-resembling, mythological, sentient structures/objects; entities (or indeed other worlds) with qualities like transforming, geometric/fractal, hyperdimensional, holographic or organic-mechanic; or which communicated visually, imparting specific messages (surrounding oneself or the world) were entirely absent from the NDEs. Additionally, all descriptions of the otherworldly space, like regular human worlds, outer-space, artificial scenes, infantile scenarios, or grid-like spaces were equally missing. Similarly, explicitly organic, technological, geometric, or symbolic/glyphic items were not apparent. In terms of themes at onset of the DMT trip, the sensorial, and psychological experiences occurring (in Michael et al (10)) – all onset features (submergence, the rush, anxiety, pain, reality break-down etc) were not in the NDE narratives, yet these may be symptoms of the initial effects of drug administration. Equally, it is of note that virtually all NDErs don’t elaborate on such initial building of their experience and instead seem to ‘awaken’ (from unconsciousness to consciousness) to a scene. For similar reasons, no open-eye experiences (energy, break-down) are reported in the NDE, yet that synaesthesia or (non-musical) sounds are also not reported is of note. Finally, cognitive disturbances and contraction of time were also not reflected in the NDEs.

**SM 11.**

## **Endogenous psychedelics’ (entropy) & high frequency oscillations’ (gamma) potential role in NDEs**

There are also significant theoretical foundations for considering the possibility of endo-psychedelics, like DMT, to be physiologically elevated in near-death states. Recently, Scott & Carhart-Harris, and Gossieries & Martial (89,90) have suggested the administration of psychedelic compounds to patients with disorders of consciousness (DoC, ranging from deep comatose to unresponsive wakefulness syndrome (UWS) to minimally conscious states). This is owing to entropy and other complexity measures of consciousness being known to be reduced in such states (91), and recent evidence of psychedelic substances to elevate such entropy/complexity (92,93). In short, a naturally-evolved endogenous version of this may be transpiring during NDEs, accounting for the apparently paradoxical awareness reported by NDErs during ostensibly unconscious states. If such DoC patients do not become behaviourally responsive upon psychedelic treatment, they may still report experience if they recover later, which is analogous to retrospective reports of NDEs from those surviving near-death conditions – and if only a fraction of DoC patients report such experiences, this may be a similar to proportion survivors with NDEs, and the content may be comparable to NDEs themselves. Alternatively, if they do not behaviourally respond, brain complexity measures (such as Lempel-Ziv Complexity; LZC) may still be increased and theoretically, though without reporting as such (unless with later waking), they may still be having internal experience – but one of disconnected consciousness (where complexity may support enriched consciousness, but brain networks are still sufficiently damaged to prevent engagement with external world (94)). Given psychedelics increase of entropy/complexity, e.g. as measured by Perturbational Complexity Index (PCI), and their subjective enhancement of conscious *content,* that is, richness of experience, over arousal/wakefulness, that is, level of consciousness *per se* (89)*,* it may result that only such disconnected states of consciousness, akin to the REM state, occurs. This said, increased range of conscious content may also correspond to increased *level* of consciousness (92,95–98). Tellingly, tests of psychedelics’ increase of LZC/PCI and the behavioural response in sedated animals, sedated humans and asleep humans demonstrating a possible promotion of REM (99-101) – as well as 5-HT2A *ant*agonists showing increases in slow-wave non-REM comparable to the UWS brain activity (102,103) – acts as substantive support for the idea that if endo-psychedelics may be released near-death, they may indeed be largely responsible for engendering NDE, including those during anaesthesia.

The question of the neurochemistry of the near-death experience is clearly more than just a question of endogenous DMT. Briefly, not only does this consist of the mass neurotransmitters exocytosis described in the main text’s description of rodent experimental death (e.g. 86), but also high-frequency, gamma range cortical activity in humans (104,105), which is of global coherence and synchrony in many rat models (106). Critically, Vicente et al (137), in a single dying human being, showed increases in absolute gamma power after global neural suppression, and relative gamma after cardiac arrest, including cross-frequency coupling between gamma and alpha & theta. Very similarly, Xu et al (138), in 2/4 patients post-life support withdrawal, demonstrated the same, alongside increases in interhemispheric functional & directed connectivity in gamma, and in multiple frequencies around the temporoparietal junction (centrally implicated in OBEs, dreaming and consciousness itself). Such evidence can presage a so-called death of the ‘dying brain paradox’ of NDEs (wherein not only conscious, but elaborate and lucid experiences are possible during a time of ostensible neural impairment), and a movement instead to a *hyper-functional* ‘dying brain hypothesis’.

**SM 12.**

## **DMT’s potential role in the NDE**

The question also remains that if indeed DMT (or other endo-psychedelics, or indeed any neural processes at all) are involved in the NDE – although DMT release has evident neurogenic, neuroprotective and anti-inflammatory effects which would have been favourably selected for as a physiological survival stratagem – why should the release of any chemical with such properties also prove to have such profoundly baroque, psychedelic and mystical, or even experiential effects at all? Some partial response to this may be that the *experience* of the NDE *per se* confers adaptive prosocial after-effects, due to which it may have been replicated via inductive methods by shamanic societies (111) – and similarly, the shared cosmology resulting from some memetic transfer of such a return-from-death narrative may have historically lead to coherence between the community (112). Thus the NDE may have conferred genetic propagation advantages, eventually coming to constitute an ‘inherited predisposition’ (112) – where, though ‘experiences’ of those not returning from death cannot be known, by definition, near-death experiencers are survivors, and so the NDE (alongside its physiological elements) may confer survival benefits (113, or 121 regarding instances of spontaneous disease remission). The positive group influence of NDEs is akin to suggestions that the entheogenic psychedelic experience amongst indigenous groups is employed to secure belief transmission across members for similar binding purposes (114). Equally, the plethora of evidence for the advantageous sequelae of the psychedelic *experience* - like therapeutic outcomes, as mediated by the mystical experience (115–117) or insight (118), or in other domains like personality (119,120) - is also supportive of the evolutionary adaptivity of a psychedelic-like experience near death. Finally, specific features of the NDE such as the life review may also be a conserved adaptation due to possibly representing the brain’s attempt to search the repertoire of past experiences to avoid the threat of death (in predictive processing terms, to select across cortical priors to explain the cause for the novel sensation of dying and finally generate action for survival). The dissociation and analgesia such substances can produce are evidentially valuable psychological advantages for the protection of the experiencing self in near-death conditions.

**SM 13.**

## **Less Typical Motifs**

**Birth Imagery**

*BJ_fmp319*

“…my life passes through me again, and I travel the whirlwind in the opposite direction from just now until *before* [italics added] my birth. But I can no longer stop scrolling, although I am for a moment curious to see my birth and especially to try to capture my sensations of that moment”

**Death/Skeletal Imagery**

*JM*

“Not a lot of menace this time, but a lot of hints at death but in a comical way… Definitely hints at death, which is quite specific, but in a mocking way…

A constant feeling of being pointed to *look over there*, but you know you shouldn’t, like a trick… And as I said the death thing, hints at the skulls, but they’re being laughed at and dismissed. This is the most specific message…

Some of it was jovial, with little bit of menace… Again about ‘the secrets’, *Have you seen the thing*?... yeah, a definite point of dismissal of death, so that’s a communication”

**Etheric Body**

*FR79*

“I found myself split into two parts. My body was resting in bed and from the top of a ‘cloud’ I could see myself. My double on the bedroom ceiling was witnessing an extraordinary scene in sharpness and authenticity… And me, a little higher [than others witnessed near a river in another world], lying in the gallows bed – and sorry, again, me a little higher, on a translucent and solid cloud”

**Partner Lying Dead**

*MS*

“She [partner] was lying there, her dress, everything. She’s got all this plant stuff and animal life on her. She was dead in there, she was dead… She’s got a dress on, and something on with leaves, and just flowers and plants. She was levitating, and they were holding here up, all those plants on her clothes were holding her up…

There were skulls, so many skulls coming up every now and then… the bloody maze…the vividness and colours, I was just getting skulls… the skulls were the first bit…[around them] was just fractals of moving energy”

**Psyching the Psychopomps**

*LG*

“[The entities] were coming out of the walls, swirling out, and melting back into it

*Interviewer: So you had an interaction with them?...*

Yeah, no communication, but glances

*Interviewer: But recognition, they were aware of you, you were aware of them*

Definitely

*Interviewer: And they were specifically coming to check you out?*

They were paying attention. I wouldn’t say they were particularly interested, but they were like, “hmmm”

*Interviewer: ‘There's another one’? [impersonating the entities]*

Yeah, basically! [laughter]

*Interviewer: ‘Is he dead?’, ‘No, it’s just DMT’*

‘No, he’s going back, just a flying visit’

*Interviewer: …Yeah! They must get that a lot”*

**SM 14.**

## **Exemplar raw interview transcript**

*Terrance McKenna 1*

Terrance: How many times did I breathe in?

David: 4 I think

Only?

Maybe 5

Because I was seeing… I was coming back again and asking and I don’t know, 9 or 10 times then I realised that its too much, and I need to stop and calm down cause I’m definitely not in this reality anymore and I’m gone and this is not happened. That’s why I needed to ask how many times in fact

It may have been 5

It was 4

Yeah I think it was double it happened for me, at least 10 times and I was asking again and you were giving to me. And then it was like very strange fight I was trying to break this reality, to come out of my body and go there. And this was…and then again I was coming back here on the couch asking you for another smoke and now… and in the end, yeah

You were there already

Yeah yeah… you thought you were still here

And I was in… um… first, no I wasn’t in that lab… first of all I was in a dark room, room I think, it was very dark I couldn’t see the walls or the end, and everywhere was something like… an organic green thing flowing everywhere…and it was everywhere like seaweed almost, like when you are looking at seaweed under a microscope, I was able to see what’s happening inside the plant, and how they were carry (?) something and trying to show me what they were carrying inside. And I didn’t pay attention. I was still after that fight at the start of the trip. Then the message was OK if you don’t wanna listen or don’t pay attention, we have another method… then I was in a kind of lab laying on my back and they put me something basically in my face, so I couldn’t see around it only a bit, I could see them over the edge over the thing, it was…

Who were they? What did they look like?

Very human, but not… they had something on their face like doctors had

Like a doctor’s mask?

Yeah a medical mask you know. But their heads were too long and their hair was, it was long and moving by itself, like snakes… and when I was trying to look at them, they keeping telling me stuff, but they were not speaking, but trying to put me to see that…they were trying to show me. It was a kind of pill, a medicine? Even it had not, some symbols in some way, those symbols were telling me this was a medicine. But I never saw that kind of symbols before but in the trip I knew that they represent medicine. They were on the pill. And it was moving, and trying to convince me to pay attention when I was looking inside the pill somehow, I don’t know how, I could see a lot of people moving around, like I was transported into a street, but… I came back very fast because it was too much difference – a lab then looking on the street at people walking around, then I was trying to look at the pill from another way somehow. I was only looking…

Could you sketch the symbols, do you think?

Yeah… It was…another one…looking like a tree. Something changed, it didn’t look like a tree, but I cannot remember what

That’s ok, carry on telling me the story

Yeah, I saw the people, I came back then I was in the lab looking at the lab trying to figure out what they wanted me to see in that pill. And again I was trying to look how they were looking and what they were doing, and I was almost sure I was inside of a lab. A lot of lights, it were very white – very white light. Around them I could see only light, but it was also some tools, medical…not medical… what’s the furniture?…not medical. I think it was furniture…but not sure. It was something near them. I think they took that tablet, not a tablet because it was watery, on that watery liquid, it was the image of the pill… I was very curious to see what is around where I am, and they put me back in the face, and again I think then I changed my position on my belly cause I needed more darkness to see clear, and… again it appeared green seaweed that was at the beginning, which I saw at the beginning. Somehow they were trying to tell me that the pill was made of that green thing but I couldn’t recognise what that is, it was like looking at material plant under the microscope, something like this. But it was moving, it was alive, and something important was carrying in it and moving it somewhere. And then they still show me a pill still appear with symbols. I saw more symbols than these ones…it had something very distinct that I knew it was not a regular pill, but I cannot remember how it looks exactly. And in total that pill and around that pill were floating 4 or 5 symbols, different symbols – only something like this, this one for sure, and this one I think also had kind of snakes or hair – and they were floating around a pill, green with white pill [this was green, and the symbols floating around]. I couldn’t understand more than those things represent medicine

Do you know what the medicine was for?

No no no… but (long pause)… when the images were starting to fade and weren’t so vivid anymore, I saw…the green thing changed to red one, like blood vessels. But that’s why I was asking for more darkness I wanted to see more clearly. It wasn’t that very vivid green, it was red exactly like blood, but the same patterns with same things carrying in it, like I don’t know, like nutrients in a plant or something…and it was starting (?) to turn bloody. I couldn’t see more, I needed to stay couple more minutes

Where were you at this point? Was that the whole trip? So i'll go over what you’ve got so far…

I feel that before the lab part, I’m sure that I was in another place but I cannot remember. I could remember it right after I was back here but it was more, but I can remember before the lab it was something else…And when I was in the trip I was thinking I need to tell you about that

So, shall we go back to the beginning? So you smoked the pipe, you think you’re smoking it 10 times

Yeah it was repeating. But it wasn’t every time the same thing, every time it was different. I didn’t see the exact same event more times. It was a different action every time. You were doing something else every time. So only 4 times?

And remember when I asked you the first intensity?

I think so but I couldn’t respond. I hear you, but it was so strange.

What was happening?

I was fighting with myself with my body to break through. I was too focused to do this, sorry, that I didn’t respond… I’m not sure I was able. Because first I was trying to respond. Oh no! I heard you and I didn’t knew who you are, why is someone disturbing my trip!? And then I remember, Oh I am in this study… And I was thinking to answer, nah, I was there and I needed to break through

And then I asked you again, then you were able to say 9

Oh yeah?

Yeah, well a minute later, you then said 9

I don’t remember.

That’s fair enough

Oh! Was it one minute, no!?

Well the first minute you didn’t answer, then at 2 minutes you said 9

And then I said 9!? First time?

First 4 times you said 9. Remember?

After when I came back?

So no, 1^st^ minute you said nothing, then 2^nd^ you said 9 then minute 3 you said 9 then minute 4, 9 etc…

I cannot remember… wow! Really!? Wow… Woah, no! really?... I was very busy in that time. A lot of strange things was happening and trying to figure out… I cannot remember when I answered. I can remember the first time, but how it was for me, you asked me the intensity, I couldn’t answer the first time, and then hours later you ask me and I said 9, and then that’s it I was back in there for 100%, I didn’t hear any more I didn’t answer

What’s the next number you remember after that?

I think I said 8

You said 8

Yeah it was at 8 I remember that

That was about 6 minutes in, and what was happening?

Oh yeah, they started showing me the pill

Ok, so the 1^st^ 5 minutes you were seeing…

At 8 I calmed down and I stayed to see the pill and try to figure out what is it. But yeah

So first 5 minutes it was this green thing

Yeah green thing in black environment, couldn’t see the end…and it was everywhere, around me, on me

What was it like?

Like a seaweed. But very long, I could see the end, I don’t know where they were starting or ending. Basically I was floating around them. It was, I think they were somehow floating in that room. I was inside that, like, wow, how to explain…

Were you a part of it?

No I was only a visitor, an observer… they were like plants, but somehow they were communicating with me. I guess they were trying to say look inside, and try to see what they were doing for me. But I don’t know why I was so agitated so, and that’s why that’s why they sent me first to another place I cant remember, and then they send me in the lab. And then when I was on the table, bed, that lab, I heard you and I said 8…

And was the seaweed still there in the lab?

No, the seaweed disappeared completely for some time, until it appeared afterwards like to show me the pill was made by that kind of plant, seaweed, or …

And then you thought it was coming down, next thing you said was 6, remember that?

Yeah, yeah. And the image wasn’t clear anymore so wasn’t so vivid the colours were fading and, yeah that moment starting to turn bloody. And I was trying to see more but it was going already rapidly

And then the next one was a 4

Yeah at 4 I couldn’t see the bloody seaweed anymore. I saw only fractals, patterns

Geometric? How did they look?

They weren’t so… usually I can see a room full of them everywhere, but again this was in a black room, and they appear only a few. I don’t know they pop up like this, and they were growing growing, and another one appeared, growing growing. But there were 5 to 10 maximum… and then moving but also the light inside them usually is very bright… I think because it was at the end

Colourful?

Yeah yeah

And then you had 4 for little while then you said 3 and we asked you to do the visualisation

I was in a hurry…

You weren’t ready for 3. But you had a go at visualisation, what did you get?

Oh yeah… wait a second. There was something else at the end. It was like a tissue, a human tissue in fact. An open wound? But it was at the end, but still repeating the same image. I was open eyes and closing, and image was repeating, and appearing like a stressful add, pop up [?] But basically, that bloody seaweed transformed itself in that human tissue

Is this before the visualisation, before the lab, or after?

After! I think it was right after 6. Right after, I saw that bloody seaweed and then, yeah I was trying to get more darkness, and yeah, transforming in this… and I was getting frustrated cause I couldn’t see anymore. Very interesting, a human tissue. I think it was same place, like I was in the same lab somehow. I couldn’t see around, but I was sure in that moment that in that place around that tissue was were those entities or doctors…but I could see them, but I feel their presence. Like they were showing to me that

So this was after the geometry

Before, before. The geometry patterns they were fractal, the last things I saw

And remember we asked you to visualise, you thought you were on a 3 but probably a 4. You tried to close your eyes, and said I think I’m still a 4. Remember what you visualised?

Yeah it was, I think the last moment when I could see that human tissue flesh of meat…because I was seeing like it was magnified under a microscope, but then it was, I was very far away from it, like there was someone pulling my hand and taking me from there, something like this, then I was at 3 or 4 in fact

So that was your visualisation

Yeah I said 4 cause I wanted to try to come back and see a bit more

Then you went back into a 4 and what was happening then?

I was trying to see if I could see more, but I was taken away from there and put into that room with geometric room

It was like an actual place, and something took you?

Yeah! I felt that someone took me, I couldn’t see someone, but like I was dragged from there. And put me in a darkroom, and said Why I am here, why dark, dmt is not so dark? Then the geometric patterns started to pop up, Ok this is usual, dmt started to…

So then though you went to 3 then 4 again, then 3, 3 then down to 2…

At 2 I said 2 cause geometric patterns already starting to disappear, difficult to notice them.

But still feeling a bit…

My body, I had a very…that sense of floating, levitating my body. Not yet on the coach, 1 inch over the couch

How was your body right at the beginning?

Oh yeah I wanted to ask, how much did I move?

You were moving a little bit to begin with, then about minute 3 you turned over… you took your glasses off

That’s it? I was very concerned I scared you, cause for me it was very different… You (gestures to PM) at one moment you were holding my feet because I was moving too much

Definitely within your trip

I was sure, then I was somewhere at 9, and that’s why I am back at reality, and then I’m back in the trip, because I had moments when I was seeing myself here on the sofa, and moving like I had a seizure or something

No that didn’t happen

No!? I had seizures for like 10 minutes in my trip! I was so concerned I scared you

You just rolled over into the sofa…

No! That was much later I turned over!

Really? That was like 3 or 4 minutes in. You were still saying 9… but you weren’t moving that much

Wow! So I was shaking violently in my trip.

There were moments you trembling a little bit

In that moment I was doing this (gestures thrashing)

No no, you weren’t moving that much

There were certainly some trembles going on

A little but very brief

I think that was…it was very intense for me. Also, because I was concerned about you and, that you were worried about me. And I was trying to tell you… in my trip at one moment I was trying to come back in this reality cause I’m good, I don’t know why I have these strange movements, and not to worry, and let me alone to have my trip

You were mumbling something when you turned into the sofa

Saying some stuff… I heard

Something about being shown something, seeing some imagery, I heard that

Yeah, I’d a sense you said ‘They’re trying to show me something’

Oh yeah, that moment was… I had the seizure, and it was before the green thing. I had this 10 minutes at least of strange seizures, and moving uncontrollably, and thinking only how to send you a message from my body that I’m good and I’ll figure out how to… and wow… I forget it fast

Its like a dream when you come out

I’m trying to make connections but a lot of parts are missing already

So did the seizure- it felt like 10 minutes, before the seaweed

Yeah, yeah. This is how I was going in the trip. This was my breakthrough basically, my fight to go from this reality to that one. It wasn’t… I didn’t knew I was going to break through. This is how I feel that my body, my consciousness wants, not wants, but cannot break through, I think that’s why I was asking for more dmt in my trip than I had, than 9 to 10, smokes…

Honestly I’ve witnesses it was only 4 times

Laughing. Wow… and no seizures? Amazing…

Can I ask you a little bit more about the medical guys… moving like snakes you said?

Yeah like I said, so much light behind of them, so their faces were in the shade somehow, but I could see very clearly, very strange hair, from their heads, and very similar to very thin and long snakes, kind of snakes, cause I was very close, it didn’t look like snakes, only the movements – and also that snakes I think they were one one of the symbols, or two of the symbols. And they had these masks, long heads and faces…

Did you see anything on the face?

Yeah I’m trying to remember the eyes. They didn’t have the nose I think

The mask?

No, they had a flat face somehow, and the nose wasn’t… maybe they had some holes under that mask… and their eyes were much bigger than normal. Not like alien eyes, but somehow wasn’t human eyes, like more animal eyes.

Could you maybe sketch them?

Hm… it was like a lamb eye

Like, dark?

Yeah. And it was feeling only kindness. Yeah, I wasn’t scared it was like alien eye, big one. No it was like an animal, lamb. If I can find something similar, an eye similar… like a puppy or lamb eye yeah

What was their demeanour to you, also kind?

Oh yeah! A bit too insistent that, because it was my fault I didn’t pay attention, I was too, after that seizure – try to understand me, its hard to get calm. But yeah they were very good, very calm, trying to calm me down, to show me that. They were a bit too insistent, but not in a bad way, not to force me, or…I wasn’t tied to that bed or something. I couldn’t see my hands but I’m sure I wasn’t tied. I couldn’t move, but I didn’t feel anything that was keeping me there in fact, only I had my body paralysed somehow.

In the lab already?

They were staying, looking very curious like me, *at me (corrects himself),* showing me that pill, at the same time they were studying somehow at me

And they had tools?

Yeah tools, furniture. I cannot remember where they were. I couldn’t see very well cause of the light first of all. And they didn’t want…to let me to look around basically.

They kept you paralysed?

Yeah they were trying to keep me focused on that liquid laptop or something I don’t know tablet, show me that pill, a [?] inside the pill, but inside I saw only a street of people walking around. I was out in the street now, and I came back

Could you say where it was?

No, it was a very busy street. First I thought I could come back and go again in the street. I wanted to see if I was still in the pill or what happened. Cause it was like going through a wall to another dimension. I’m like this is not my place, I need to go back. And I was back in the lab, OK. Back here, with all these things around me, I need to see inside the pill, then I was seeing the green things again not the street anymore

So you had transitions, the breakthrough, the seizure

I don’t know if the seizure was before or after the break through in my opinion, I don’t know

Presuming the breakthrough happened first

When did I have a seizure?

You were still trembling near the end

I think it was prior to when you turned into the couch… you were like that (trembling), and do you think what you think was the seizure was near the beginning?

Yeah, kind of how you’d described, you were trying to get a breakthrough…

Yeah now I think how I saw those 10 times when I smoked, and… it was after the breakthrough, I wasn’t here, it wasn’t this reality… and you said I wasn’t moving at all, I’m sure

[Pascal] wasn’t holding your legs down or anything

Anyway, you helped in my trip

Glad to have been of assistance

Anything else about the beings, how many were there?

3 I think. Very long hands. But I told you they didn’t try to touch me or keep me there with their hands, no they were doing something, preparing something, like for a surgery, like they were doctors preparing their tools, but I couldn’t see properly

For your surgery?

I don’t know. One of them was there with the liquid tablet to show the pill

Liquid tablet?

Yeah like an iPad but it was liquid… yeah it wasn’t solid.

They’ve got those coming out soon

Apple 11

From time to time, the other two turned the table, and see they were preparing something like tools…to show me another liquid tablet with something else, I don’t know

If you had one word to describe them, what was the thing they’re most like?

Somehow if I need to compare with something on this planet… somehow it was octopus, I don’t know why. Even then, I saw how they look, some human but not really. Ok human, with what? And next thing they’re like octopus, from [?] another planet. They had many similarities, oh yeah like that long head here [?] very long, and the head was big, the scalp here was big, maybe that’s why similar with octopus, they had big head… and yeah octopuses, I don’t know any more similar. Its far from octopus, but that’s the closest

Anything else?

Yeah in that fight when had those seizures

You think the medicine might have been for the seizure?

No, it was something else. I think inside fight with me first of all. And I’m sure I cannot remember a lot, because I took 3 tokes, like McKenna said, then I close my eyes, I saw a lot of patterns, very vivid

So patterns at breakthrough

Oh yeah, but very quick, then the seizures start. Then I thought I missed somehow the breakthrough, and I was fighting with me, because I was trying to break through but I was already there maybe. I had that kind of seizure maybe, uncontrollable movements… then kind of black out for one second. It sent me also on the sofa, but very calm, asking me for another smoke…

Your 10^th^ toke!...

(Laughing) Exactly this was the feeling… I was on the sofa [?] Again the patterns after, every time it was a place I cannot remember, but I’ve the feeling I had the rest of the trip, then somehow I’ve the feeling I had a fight with me, my ego, somehow. This is how I feel now I didn’t know how to interpret this. I think this seizure was in fact my [attempt to breakthrough]…

Interesting, before you were just about to start, about to give yourself up to it, and started shaking

But this is usual for me. I’m very exciting

You were shaking much more then than once you smoked the dmt, not very much afterward

In my trip I was shaking like this, my head my hands my feet, you couldn’t barely keep me on the sofa

In a parallel universe maybe?

With psychedelics, I know everything will be good. Doesn’t matter if this seizure happened in that moment I wasn’t scared

You were worried about what we were thinking

Yeah I was worried about you… (laughter) it was crazy. And I was almost sure that these 10 pipes influenced these seizures

Can I just ask you, how did your body feel in your first experience?

After the first pipe?... so like you said I was shaking

That was before you smoked

Yeah, before I took first smoke [?] I was focusing to hold the smoke. With that smoke it came the feeling of relaxation and I didn’t have any worry, and also because of fibromyalgia, I have almost continuous pain in my joints, and it disappeared after 3 or 4 seconds, it completely disappeared. And taking psychedelics, this is the first symptom that they are working, I don’t feel pain

Did you feel in your body still, you were in your body, you were aware of your body the whole time?

After the 8^th^ pipe?

But it felt different?

Yeah after the 2^nd^ [toke], completely different

How?

I was levitating already. I knew it was not possible, feeling like levitating already. Also, I had it when I came back for several minutes. But it was more intense when I hold the second time I was feeling like… and I think with the 3^rd^ hit

Did you get a rush?

No no… it was strange usually I have that rush but not this time no. I was waiting for the rush

You had a wrestle instead. Any sounds?

No…

OK. If you remember anything else you can tell me

I’m already…I’m not sure what I told you

It’s a bit hodgepodge. But we’ve the number and times, we can order it…

How long did it last?

Til 1 and a bit?, about 25 minutes. First 10 minutes, no answer then 9 9 9 9, then 8, 6, 4, 4. At 10 you were at 4, then thought you were at 3, then back to 4.

So 6 minutes I was at 8 already.

The time felt?

It was one of the shorter trips I had on dmt

Actually or how it felt?

How I feel it. Cause my longest trip on dmt was more than 27 years

Oh the experience? Wow you should’ve told us before. So get a lot of time dilation

So my entire life, I lived again my entire life, and then I was, I think I was for a couple of seconds I opened my eyes. But after this experience I’m not sure. But in my trip I lived again 27 years until the moment that I was in my room taking dmt from the pipe, my wife holding the dmt, and she was holding it for me, and when I was trying to take the *first* hit, I was… so I lived my life until the moment when I took the first hit, and I was expecting that rush… nothing…

Because you’d already been tripping for 27 years

I took again, and again nothing, the second hit. And I wanted to tell my wife the dmt doesn’t work, its not good, then when I opened my mouth to tell her. The room exploded into thousand million pixels then I was in another trip for sever hours

But the actual time?

Yeah it was around 10 minutes… but this time it felt like one of the shorter trips I had.

You said the first bit felt like an hour

Yeah but still remarkably shorter

It's only like an hour and a bit

I think I never had a trip experience to last less than several… days

Wow, ok. So how long did that trip feel like?

This one? 2 or 3 hours…? I was sure [?] when I was tripping, I will break a record with this trip, because it was already one hour, but it kept going, yeah… wow

**SM 15.**

## **Tabularised proximity analysis**

| Theme | DMT Frequency / 36 (%) | NDE Frequency / 34 (%) | Proximity score (DMT % - NDE %) |
| --- | --- | --- | --- |
| Light | 9 (25) | 25 (74) | 0.49 |
|  |  |  |  |
| Tunnel-like structures (Onset) // Tunnels at onset | 7 (19) | 9 (27) | 0.08 |
|  |  |  |  |
| Time-space transcendence // Time loss | 8 (22) | 8 (24) | 0.02 |
|  |  |  |  |
| ***Entity Encounter*** | 34 (94) | 15 (44) | 0.5 |
| *Sensed presence* | 6 (17) | 2 (6) | 0.11 |
| *Omnipresence //* Universal Intelligence | 5 (14) | 2 (6) | 0.08 |
|  |  |  |  |
| *‘Otherly’ creatures //* ‘Imaginary’ entities | 26 (72) | 9 (27) | 0.45 |
| *Light Being-esque // Being of Light* | 3 (8) | 5 (15) | 0.07 |
| *Serpentine entities* | 3 (8) | 1 (3) | 0.05 |
| *Mischievous or Jestful // Trickster* | 5 (14) | 1 (3) | 0.11 |
| *Doctor/Scientist* | 1 (3) | 3 (9) | 0.06 |
| **Humans** | 6 (17) | 15 (44) | 0.27 |
| *Deceased relatives* | 2 (6) | 8 (24) | 0.18 |
| *Communication: Returning to life* | 0 | 7 (21) | 0.21 |
| *Communication: Message*    *of Mission* | 0 | 4 (12) | 0.12 |
| *Intuition or Telepathy* | 13 (36) | 4 (12) | 0.24 |
|  |  |  |  |
| Ego death | 11 (32) | 1 (3) | 0.29 |
| Universal unity | 9 (25) | 3 (9) | 0.16 |
| Noetic experience // Omniscience | 8 (22) | 5 (15) | 0.07 |
|  |  |  |  |
| ***Other Worlds*** | 36 (100) | 14 (42) | 0.58 |
| *Plants & Flowers // Natural scene* | 10 (28) | 4 (12) | 0.16 |
| *“Spirit world” // “Heaven” or Hell* | 1 (3) | 4 (12) | 0.09 |
| *Laboratory // Operating clinic* | 1 (3) | 1 (3) | 0 |
| *Dark space/void-like* | 2 (6) | 13 (38) | 0.32 |
| *‘Limbo’ // Waiting room* | 4 (11) | 1 (3) | 0.08 |
|  |  |  |  |
| OBE | 0 | 12 (35) | 0.35 |
| *Witnessing body* | 0 | 9 (27) | 0.27 |
| Etheric body | 2 (6) | 3 (9) | 0.03 |
|  |  |  |  |
| Sense of dying (Onset) // Awareness of death | 6 (17) | 9 (27) | 0.1 |
|  |  |  |  |
| Real/Hyper-real | *18 (53)* | 9 (27) |  |
|  |  |  | 0.26 |
|  |  |  |  |
| Life Review | 2 (6) | 8 (24) | 0.18 |
| *Judgement* | 0 | 4 (12) | 0.12 |
|  |  |  |  |
| Ineffability | *19 (53)* | 4 (12) | 0.41 |
|  |  |  |  |
| Threshold/Border | 0 | 3 (9) | 0.09 |
|  |  |  |  |
| Loving or Connected | *10 (28)* | 3 (9) | 0.19 |
|  |  |  |  |
| Co-creation/Lucid dream-like | 10 (28) | 3 (9) | 0.19 |
|  |  |  |  |
| Deus Ex Machina | 3 (9) | 2 (6) | 0.03 |
|  |  |  |  |
| Geometry (During) // Colourful patterns | 16 (44) | 1 (3) | 0.41 |
